# Fast Microwave-Assisted Ullmann-Type Coupling for the Synthesis of Multisubstituted Diaryl Ethers

**DOI:** 10.1021/acsomega.6c07618

**Published:** 2026-08-25

**Authors:** Dario Gentili, Gabriele Lupidi, Francesca Stella, Jo’ Del Gobbo, Miriam Caviglia, Luca Barigelli, Maura Pellei, Carlo Santini, H. V. Rasika Dias, Enrico Marcantoni, Cristina Cimarelli, Serena Gabrielli

**Affiliations:** † 18959 Università degli Studi di Camerino, School of Science and Technology - Chemistry Division, Via Madonna delle Carceri, Camerino 62032, Italy; ‡ Department of Chemistry and Biochemistry, The University of Texas at Arlington, Box 19065, Arlington, Texas 76019-0065, United States

## Abstract

A mild and rapid microwave-assisted Ullmann-type C–O coupling protocol catalyzed by copper­(II) complexes was developed and optimized for the efficient synthesis of a wide variety of diaryl ethers. The synthetic methodology is general and tolerant toward both electron-rich and electron-poor phenols and aryl halides. Furthermore, this protocol provides a practical and rapid methodology for preparing diaryl ethers with potential biological and pharmacological relevance and represents a rapid and operationally efficient alternative to conventional thermal methods, thereby widening the use of copper catalysis in organic synthesis.

## Graphical abstract

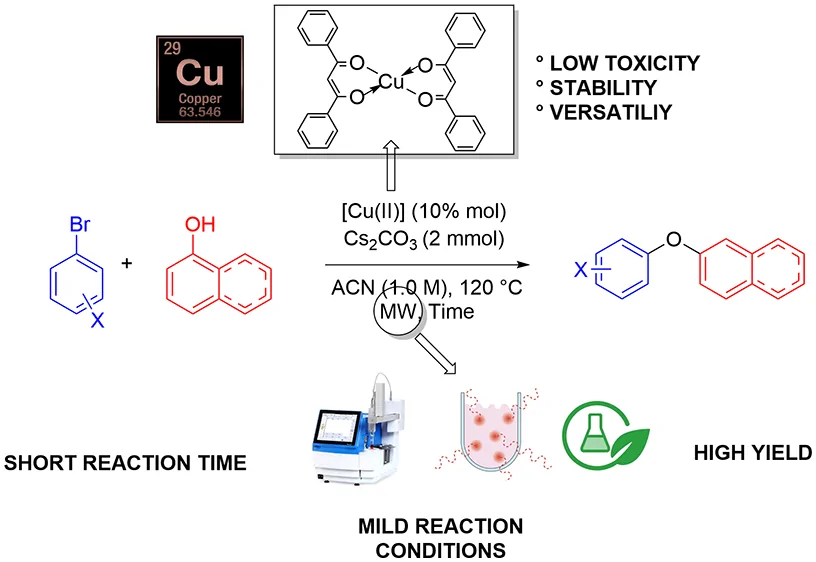

## Introduction

Since its discovery in 1901, the copper-catalyzed Ullmann reaction has been extensively studied by the scientific community.^1^ This sustained interest has led to the development of several modified versions of the reaction, including the Ullmann–Goldberg,^2^ Ullmann–Hurtley,^3^ and Ullmann–Ma^4,5^ reactions. The Ullmann reaction has garnered significant attention due to its broad applicability in forming new aryl C–C and C-heteroatom bonds, such as C–N, C–O, C–S, C–P, and C–Se.^6–8^ As a result, it has proven to be an especially valuable protocol for synthesizing heterocycles, medicinal compounds, agrochemicals, natural products, and materials for advanced applications.^9,10^

Recently, a total synthesis of tyclopyrazoflor, a well-known insecticide, was reported, in which an Ullmann reaction was employed between a pyrazole derivative and 3-bromopyridine in the presence of catalytic amounts of CuCl and DMEDA as a ligand, achieving the intermediate in a very good yield.^11^

Although copper-catalyzed Ullmann coupling between an aryl halide and phenols/alcohols^12^ is an alternative for palladium-catalyzed diaryl ether/alkyl aryl ether synthesis,^13,14^ harsh reaction conditions, the usual need for a stoichiometric amount of copper or copper salts, catalysis under refluxing conditions, long reaction times, and low to moderate yields have generally limited its use, especially on a large scale. Notable advances in the use of ultrasound^15^ or alternative bases^16^ required for the transformation have been reported; however, these have not widened the application of the reaction.

Since 2003, several protocols have been developed based on this copper-catalyzed coupling, exploiting different combinations of copper sources, cocatalysts, ligands, and solvents that allow the study of the reactivity of both bromo- and iodo-benzenes with even better results. Although there were improvements in yields and generality, these procedures were affected by long reaction times, high temperatures, and the use of toxic solvents in some cases.^17–21^

Only in the past few years have considerable efforts been taken to improve the efficiency of Ullmann-type coupling reactions using catalysts based on copper­(I) and copper­(II) complexes with heteroatom-containing chelate ligands, which enabled the coupling reactions to proceed smoothly under relatively mild conditions in the presence of catalytic amounts of copper complexes.^5,7,8,10,22–31^ To date, hundreds of bidentate ligands, including amino acids,^21,31^ 1,10-phenanthroline,^32^ neocuproine,^33^ diamines,^34,35^ diimines,^18^ phosphines,^36^ β-keto esters,^37^ aryl boronic acids,^38^ bipyridine,^39^ 1,1,1-tris­(hydroxymethyl)­ethane,^40^ diol-based ligands,^41^ carboxylic acids,^42^ β-diketones,^43^ etc., have been developed to promote copper-catalyzed Ullmann-type coupling reactions under relatively mild conditions, and in this framework, β-diketonate ligands are of particular interest. In fact, they exhibit an exceptionally weak ligand field and predominantly ionic interactions with the metal ion^44^ and at the same time, the ligand framework is π-acidic and has been described as pseudoaromatic, highlighting its strong binding efficacy with coordinated metals.^6,7,45–49^ It is often noted that even modestly sterically hindered ligands yield improvements over the parent acetylacetone, as recently reported.^47,50–55^

Also, ferrite nanoparticles or ferromagnetic carbon nanotube-supported copper complexes were studied as catalysts, which present the advantage of recoverability and reusability^56,57^ at the expense of some difficulty in the preparation of the catalyst and the use of toxic solvents for long reaction times and high temperatures.

In recent years, microwave-assisted copper-catalyzed cross-coupling for the generation of carbon–carbon and carbon-heteroatom bonds has attracted widespread attention, largely due to milder reaction conditions^57–60^ together with important operational advantages such as shorter reaction times, simpler procedures, higher yields, improved product purity, and better control of the reaction.^61–67^ Other published Ullmann-type reactions use more powerful microwaves^68^ and Cu(0)- and Cu­(I)-based catalysts,^69,70^ but all have a very poor number of examples. Instead, we discuss here a Cu­(II)-complex-based protocol, supported by numerous examples that demonstrate the generality of the method.

Our investigation began with the synthesis of diaryl ethers, a class of compounds widely found in polymeric scaffolds in the life sciences and in natural products, such as isodityrosine and its derivatives. Compounds such as Galeon and Riccardin B or the cyclic peptide K 13 also exhibit significant biological activities (Scheme 1) as antifungal, anti-inflammatory, antiviral, antibacterial, anticancer, antitumor, and analgesic properties.^71–73^ Additionally, diaryl ethers are present in many herbicides, as they can inhibit, for example, the activity of protoporphyrinogen oxidase (PPO) in weeds.^17,18,71,74,75^

**Scheme 1:**
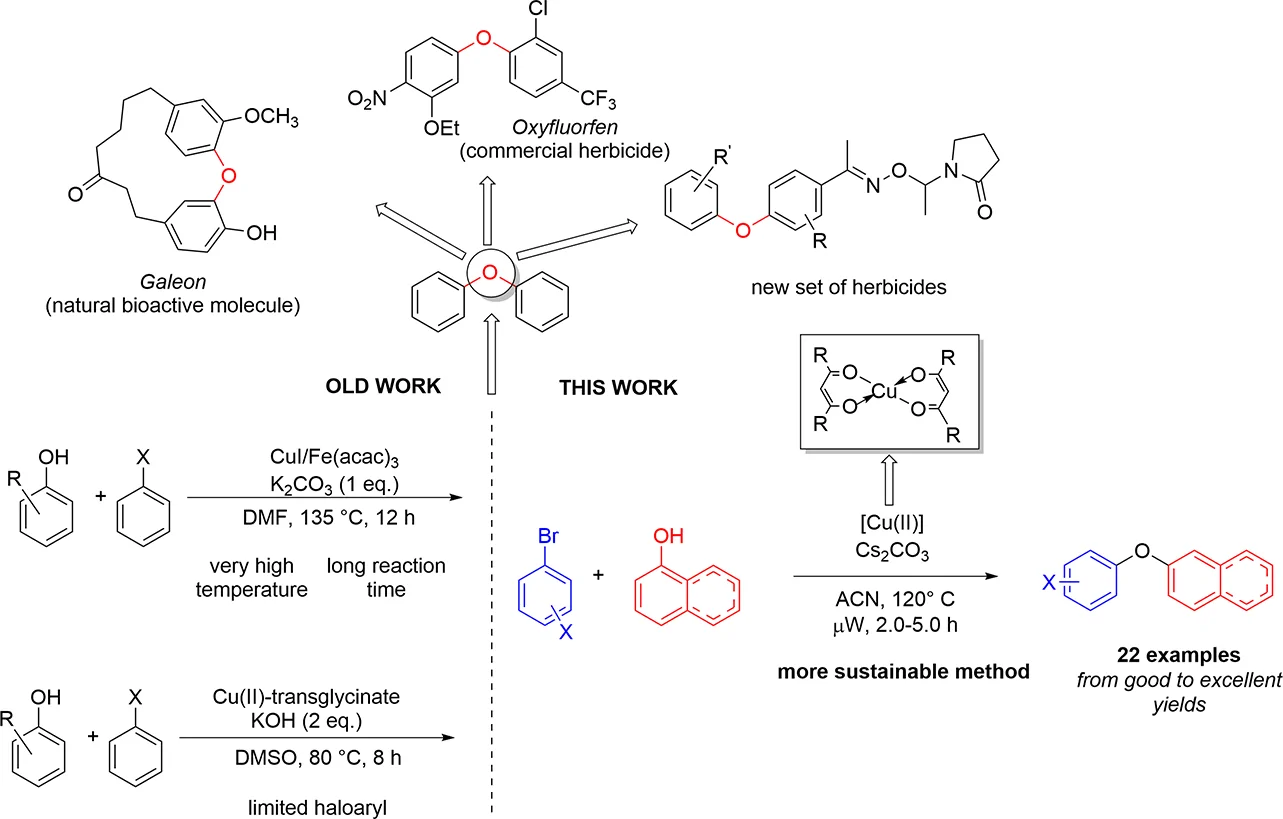
Old and New Methods for Ullmann Condensation Reaction

Previously, we reported the synthesis, structural characterization, and antitumor activity evaluation of Cu­(II) complexes supported by sterically demanding β-diketone anions.^76^ Here, we report a new protocol for the Ullmann-type coupling reaction for the synthesis of diaryl ethers under microwave irradiation, catalyzed by neutral copper­(II) β-diketonates **5a**–**e** (Table 2), together with the newly synthesized Cu­(II) complex **5f** and two ionic Cu­(II) complexes, **5g** ^77^ and **5h**,^78^ just studied as efficient systems for Kharasch–Sosnovsky copper-catalyzed C–H oxidation in allylic functionalization. Given the substantial progress achieved in copper-catalyzed Ullmann etherification, the present study seeks to develop an alternative microwave-assisted protocol based on Cu­(II) β-diketonate catalysts, with particular emphasis on short reaction times and on how the steric and electronic properties of the ligands affect catalytic performance. This method has proven highly effective for the synthesis of diaryl ethers, providing the target products in just a few hours with good to excellent yields.

## Results and Discussion

This work began by exploiting the complex **5a** as a catalyst for the Ullmann reaction, using dimethylformamide (DMF) as the solvent, yielding 89% in 20 h at 100 °C.^79^ Thus, our investigation began with the exploration of batch conditions, where bromobenzene **1a** and 2-naphthol **2a** served as pilot substrates in the reaction catalyzed by the Cu­(II) β-diketonate complex **5a**.^76^

From the beginning, product **3aa** was isolated in batch from DMF, with a 55% chromatographic yield after 24 h of reaction (Table 1, entry 1). Using other solvents, a significant improvement was observed, with the highest yield achieved using acetonitrile in the presence of Cs_2_CO_3_ (Table 1, entries 2–4).

**Table 1:**
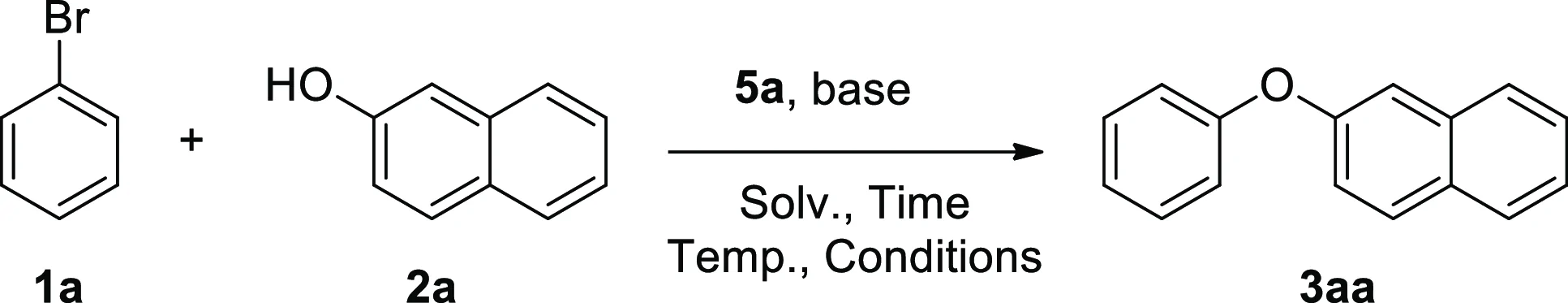
Optimization of Reaction Conditions^a^

**Entry**	**Solvent**	**Base**	**Heating**	**Time (h)**	**3aa** **(Y%)**
**1**	DMF	Cs_2_CO_3_	Batch	24	55
**2**	DMAc		Batch		72
3^b^	PhMe		Batch		59
4^b^	ACN		Batch		76
**5**	ACN	Cs_2_CO_3_	μW	3.5	88
6^c^	ACN		μW		56
7^d^	ACN		μW		71
**8**	EtOAc		μW		69
**9**	2-MeTHF		μW		-
**10**	DMF		μW		75
**11**	DMAc		μW		77
**12**	ACN	no base	μW	3.5	-
**13**	ACN	NaOH	μW		25
**14**	ACN	DIPEA	μW		-
**15**	ACN	Carbonate supported on polymer (500 mg/mmol)	μW		12
**16**	ACN	K_2_CO_3_	μW		82

a Typical reaction conditions: 1 mmol of bromobenzene **1**, 1.5 mmol of 2-naphthol. **2**, 2 mmol of base, solvent 1.0 M, 120 °C, 3.5 h reaction time and 10% mol of **5a**.

b Reaction performed in a sealed vial.

c Molar ratio **1**:**2** = 1.0:1.0.

d Molar ratio 1:2 = 1.5:1.0.

After the first screening, the switch from batch to microwave conditions successfully isolated product **3aa** in 88% yield after only 3.5 h (Table 1, entry 5), once again confirming the remarkable power of microwave irradiation to accelerate reaction rates. When compared under the respective optimized conditions explored in this study, microwave heating enabled the formation of **3aa** in a higher yield and in a markedly shorter reaction time than conventional batch heating. The batch kept for 3.5 h led to a very poor yield and the recovery of unaltered starting materials.

The variation in the substrate ratio, by altering the amounts of **1a** or **2a**, respectively, decreased the yield in both cases (Table 1, entries 6 and 7).

Then, several solvents were tested, yielding results comparable to those obtained in acetonitrile (Table 1, entries 8–11), except for 2-MeTHF (Table 1, entry 9), which produced a complex mixture of unidentified products. The reaction was also tested at different temperatures and yielded lower yields at both lower (90 °C, 53%) and higher (150 °C) temperatures. At 150 °C, the product was isolated in a 67% yield, while bromobenzene was not detected, likely due to its degradation.

Next, we explored the role of the base because, as shown in Table 1, entry 12, the reaction does not occur in its absence. Although NaOH is a stronger base than carbonate, it proved unsuitable for this protocol (Table 1, entry 13), likely because the Cu­(II) β-diketonate complex is intrinsically hydrolytically unstable, forming hydroxylated or polymeric species that disrupt its coordination environment and impair catalytic activity.^80^ On the other hand, when DIPEA was employed, only the starting materials were recovered (Table 1, entry 14).^16^ The use of a polymer-supported carbonate base also failed, yielding only a small amount of product **3aa** (Table 1, entry 15), suggesting that under heterogeneous conditions, a tight contact between the base and the starting materials does not take place. Finally, when employing potassium carbonate, the yield was comparable to the one obtained with cesium carbonate (Table 1, entry 16). Although K_2_CO_3_ afforded a comparable yield, Cs_2_CO_3_ was retained as the standard base because it consistently provided the best result under the optimized conditions, given also its higher solubility in ACN. After that, a catalyst screening was conducted to assess the activity of complexes **5a**–**h** supported by ligands **4a–g,** which vary in electronic and steric properties. For comparison, the performance of different commercial copper­(II) and copper­(I) salts was also assessed, as reported in Table 2, entries 9–11.

As stated above, the reaction catalyzed by complex **5a** gave the highest yield. Instead, using complex **5c**,^81–84^ in which the aromatic ring of the ligand bears electron-withdrawing −CF_3_ groups, the yield drops to 75% (Table 2, entry 3). We then tested complexes **5b** and **5d**, both bearing highly hindered aliphatic or aromatic substituents, and obtained similar yields (Table 2, entries 2 and 4). Complex **5f** showed a scarce yield, probably due to the conformation of the long aliphatic side chain that may hinder the alkoxide attack, disfavoring the overall reaction mechanism. Like the aromatic complex **5c**, the −CF_3_ group in the aliphatic complex also lowers the yield (Table 2, entry 5). Complex **5d** and the corresponding dinuclear **5h**, both obtained from ligand **4g**, showed very poor yields (Table 2, entries 6 and 7). Lastly, we tested the commercially available Cu­(II) derivatives **5i**–**k**, which, in each case, gave lower yields than those obtained using complex **5a**.

**Table 2:**
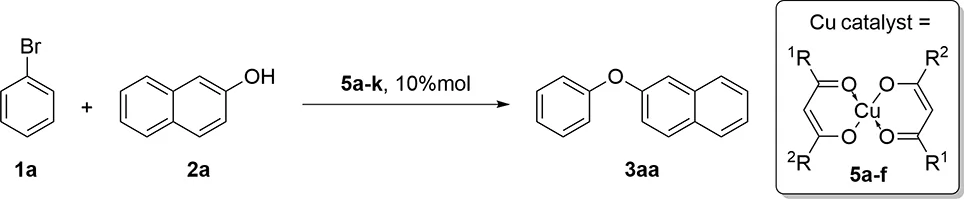

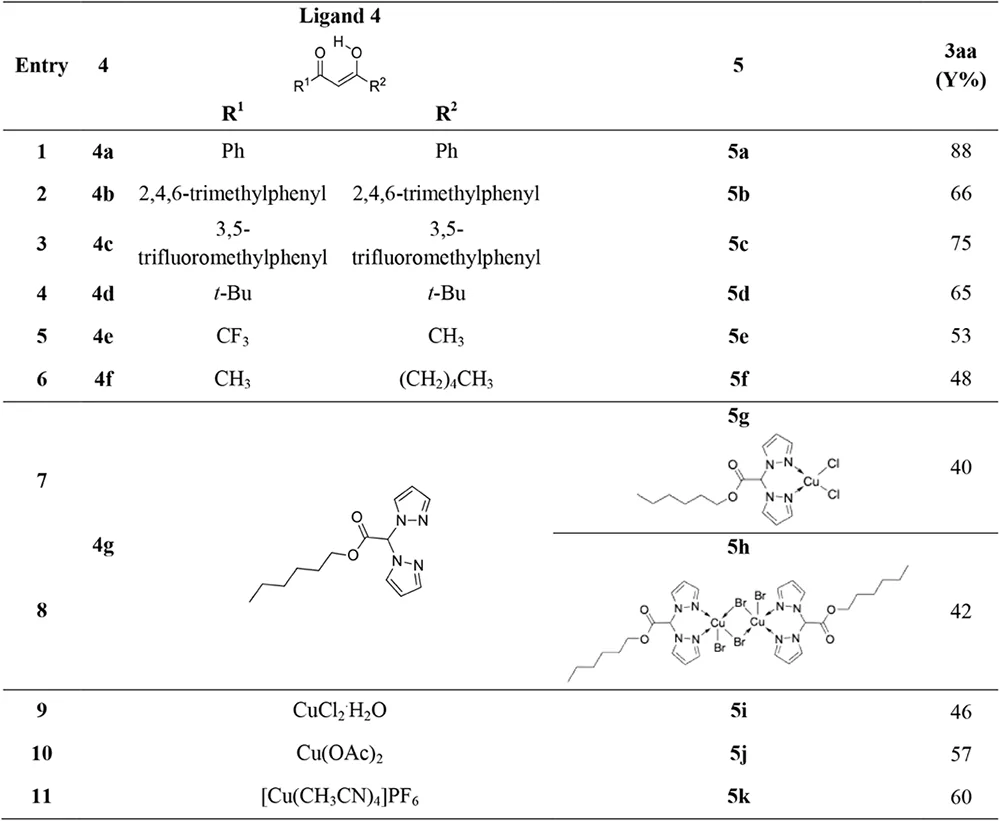
Screening of Catalyst Cu­(II) Complexes^a^

a Typical reaction conditions: 1 mmol of **1**, 1.5 mmol of **2**, 2.0 mmol of Cs_2_CO_3_, ACN (1.0 M), 120 °C, μW irradiation, 3.5 h reaction time and 10% mol of **5**.

Further experiments aimed at determining the optimal amount of catalyst **5a**, as reported in Table 3, revealed that a 10% molar amount of the catalyst gave the best result (Table 3, entry 3). As expected, the presence of the catalyst is fundamental, as no reaction occurred in its absence (Table 3, entry 1). Both 5% and 15% catalyst loadings gave yields below 10%. In the second case, the formation of unidentified side products was also observed, probably due to the degradation of the reagents by the excess copper catalyst.

**Table 3:**
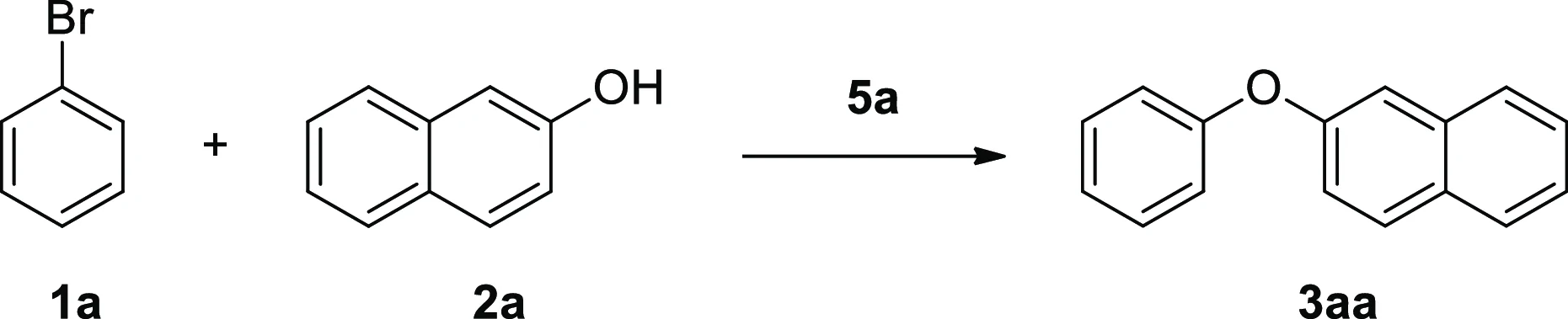
Optimization of the Catalyst Amount^a^

**Entry**	**Catalyst** (% mmol)	**3aa (Y%)**
**1**	-	-
**2**	5	60
**3**	10	88
**4**	15	60

a Typical reaction conditions: 1 mmol of **1**, 1.5 mmol of **2**, 2.0 mmol of Cs_2_CO_3_, ACN (1.0 M), 120 °C, μW irradiation, 3.5 h reaction time.

From a mechanistic perspective, it is challenging to determine the exact pathway followed by our copper-based catalyst.^85^ Although a definitive mechanistic assignment cannot be established from the present data, the precedent literature suggests that Cu­(I) is typically the catalytically active species in Ullmann-type couplings. When Cu­(II) complexes are employed, we can suppose three different paths: an *in situ* reduction under the reaction conditions (the involvement of single-electron transfer, SET),^6^ a cross-coupling via a nonradical step,^86^ or the formation of radical species. The reaction was performed in the presence of sterically hindered radical scavengers such as 2,4,6-tri-*tert*-butylphenol and 2,6-di-*tert*-butylphenol.^87^ In both cases, no effect on the reaction yield was observed: complete conversion of bromobenzene, selective formation of the target product in 88% yield, and no side products resulting from reactions between bromobenzene **1a** and the phenol radical scavengers. In accordance with the observed data, no biaryl radical homocoupling products were detected in our system.

Once the reaction was optimized with respect to the conditions and the catalyst, the generality of this microwave-assisted Ullmann’s coupling was explored using several phenols, as reported in Table 4.

**Table 4:**
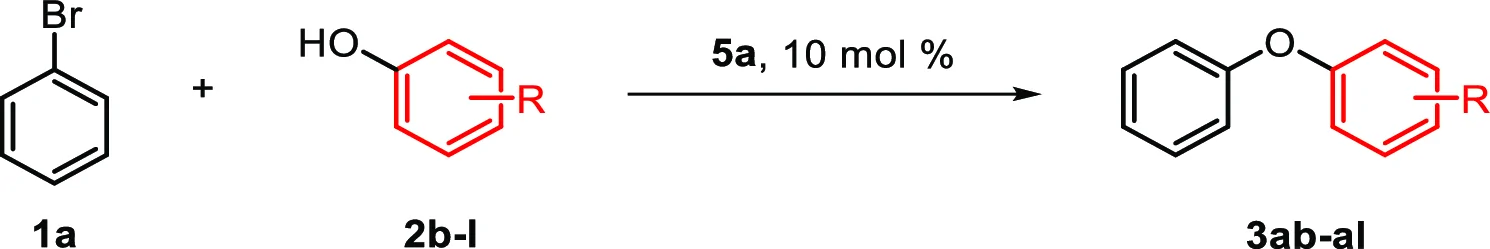

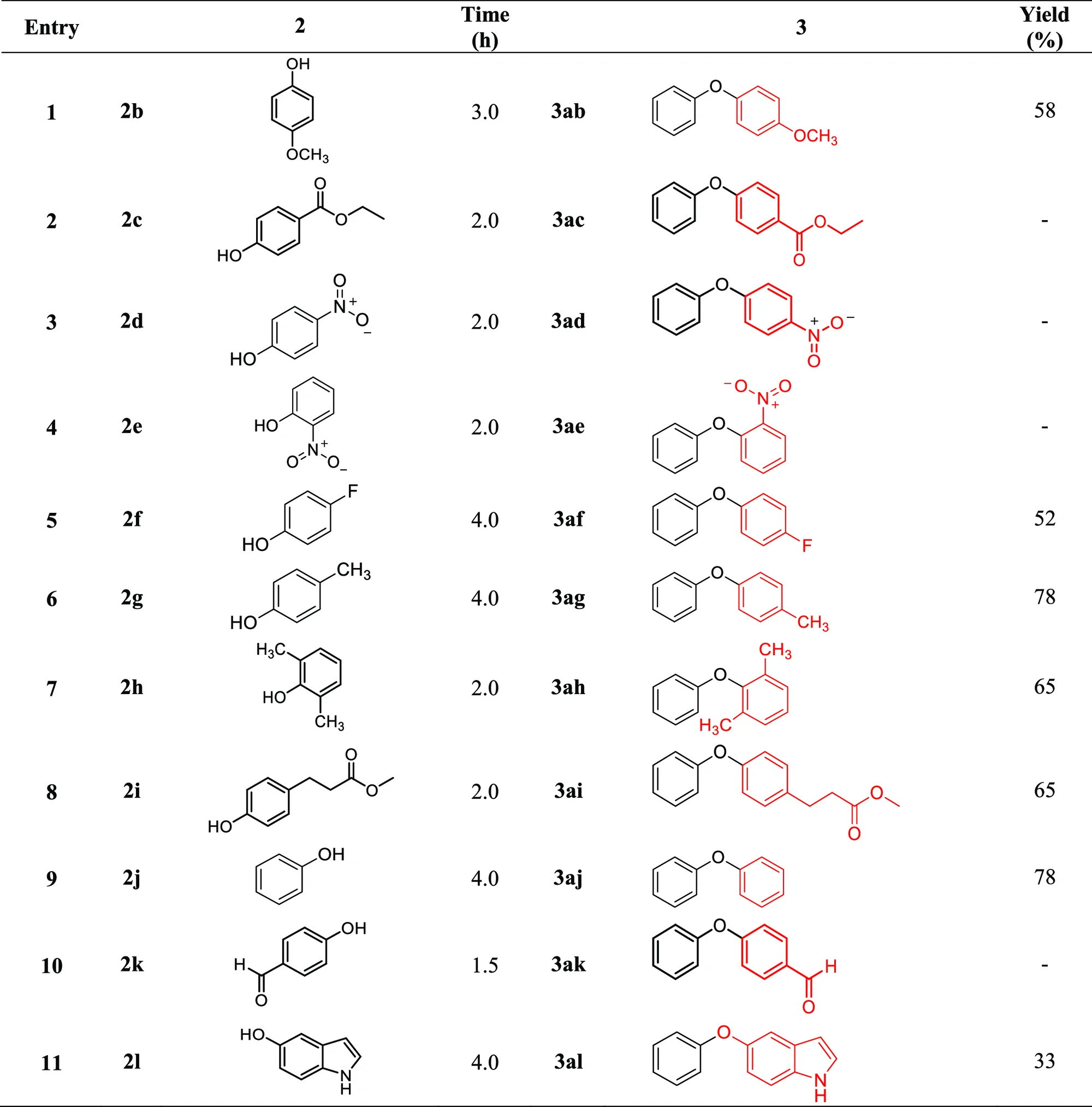
Screening of Phenol Derivatives^a^

a Typical reaction conditions: 1 mmol of **1a**, 1.5 mmol of **2**, 2.0 mmol of Cs_2_CO_3_, ACN (1.0 M), 120 °C, μW irradiation, 3.5 h reaction time.

An activated phenol, such as *p*-methoxyphenol **2b,** successfully led to the formation of product **3ab** (Table 4, entry 1), with no evidence of methoxy group modification. Additionally, other electron-donating substituted phenols were tested, such as *p*-cresol **2g**, the sterically hindered 2,6-dimethylphenol **2h** and methyl 3-(4-hydroxyphenyl)­propanoate **2i,** and the corresponding products were isolated in good yields (Table 4, entries 6–8). Notably, the reactions were performed using safe solvents and inexpensive catalysts and proceeded in short reaction times, yielding products comparable to or exceeding those previously reported in the literature.^52,88^ Unfortunately, nitro-substituted phenols **2d** and **2e** resulted in unreactivity under these conditions (Table 4, entries 3 and 4), probably because the electron-withdrawing substituent stabilizes the negative charge of the phenoxy ion and does not favor the attack on bromobenzene or the formation of a possible phenoxide-copper intermediate.^18^ It is of particular interest that the formation of compound **3af** (Table 4, entry 5), as the incorporation of fluorine into the final product opens promising avenues for the development of new diphenyl ether derivatives with potential biological applications, given fluorine’s well-known role in pharmacology.^89^ In contrast, the reaction with *p*-hydroxybenzaldehyde **2k** resulted in no detectable amounts of starting materials or product **3ak** (Table 4, entry 10). Conversely, the reaction with unsubstituted phenol proceeded efficiently, leading to diphenyl ether **3aj** (Table 4, entry 9). Finally, when 5-hydroxyindole **2l** was used, product **3al** was isolated in low yield (Table 4, entry 11). However, the most intriguing result was the absence of functionalization at the -NH group of indole, suggesting high chemoselectivity toward the −OH group.

The generality of the method was also studied under the point of view of the brominated reagent, where *p*-bromotoluene **1b** proved to be a versatile substrate, and the product **3ba** was isolated in good yield, similar to *p*-bromoanisole **1c** that led to the product **3ca** with a very good yield (Table 5, entries 1 and 2).

**Table 5:**
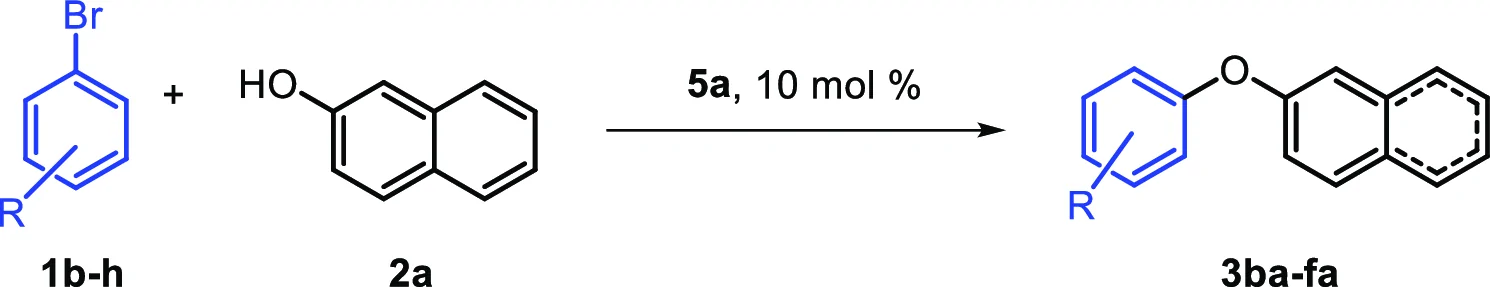

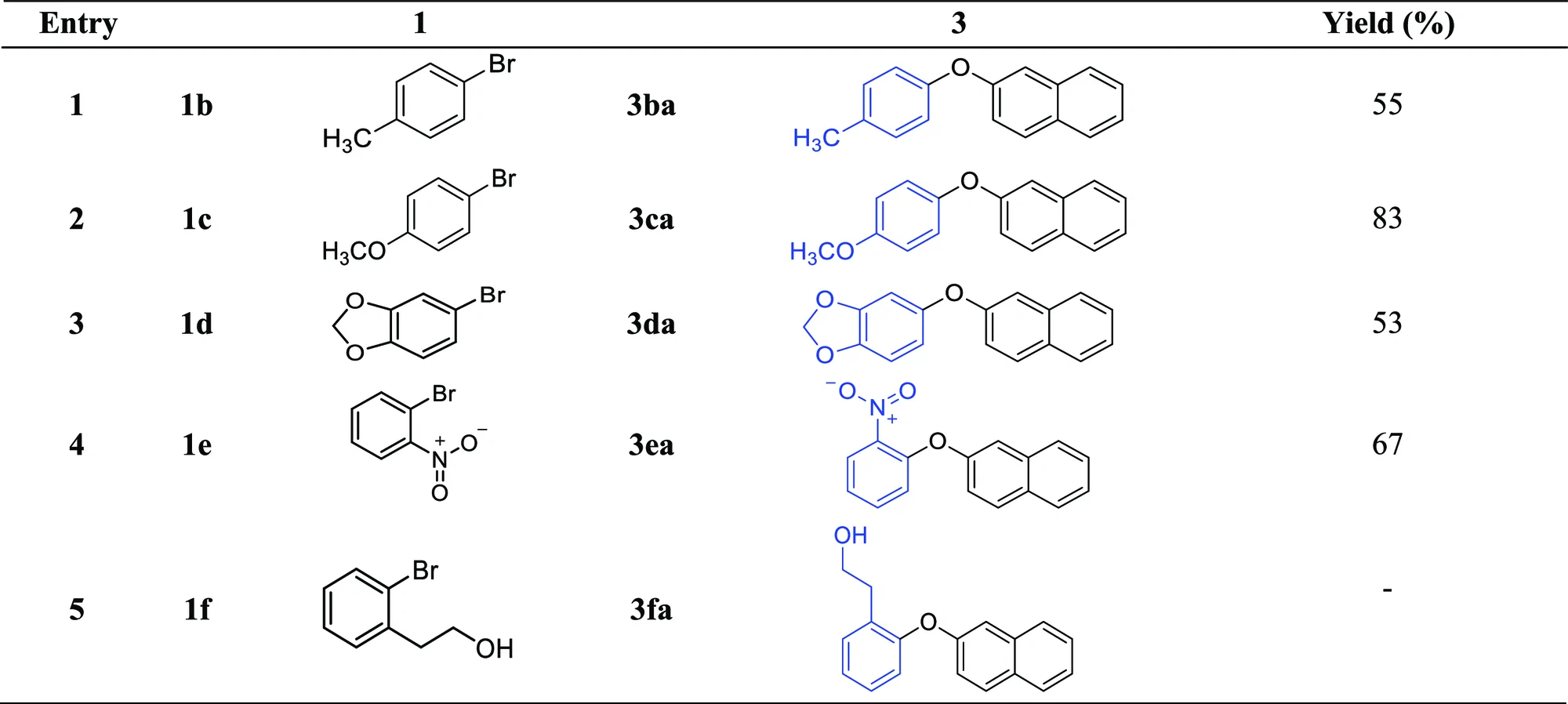
Ullmann’s Coupling of Bromobenzenes^a^

a Typical reaction conditions: 1 mmol of **1**, 1.5 mmol of **2**, 2.0 mmol of Cs_2_CO_3_, ACN (1.0 M), 120 °C, μW irradiation, 3.5 h reaction time.

Notably, product **3da** was also isolated in good yield, without observing any alteration of the dioxolane ring (Table 5, entry 3). The reaction with *o*-nitrobromobenzene **1e** successfully led to the formation of product **3ea** (Table 5, entry 4), and this is probably due to a disfavored formation of the intermediate between Cu­(II) and the haloaryl compound. Reactions using 5-bromofurfural and (2-bromophenyl)­methanol were unsuccessful, with no product formation observed. When 2-(2-bromophenyl)­ethanol **1f** was employed, the reaction did not yield the target product **3fa** but the dihydrobenzopyran **3fa’** in a yield of 42% (Table 5, entry 5).^90^ This result may be due to the favorable position of the −OH group with respect to the bromine atom in the starting material that, in the presence of both copper salts and a base, facilitates the formation of a new five-membered ring by a fast intramolecular cyclization, as previously reported in the literature and depicted in Scheme 2, rather than the attack by 2-naphthol **2a** ^91^

**Scheme 2:**
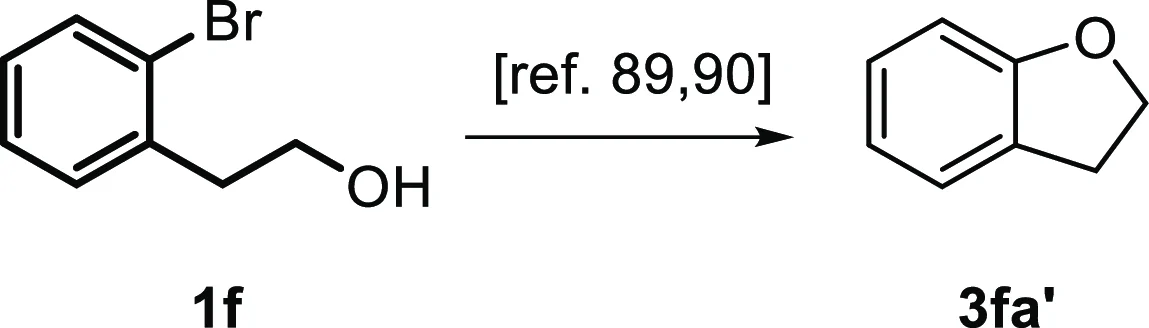
Intramolecular Cyclization of 2-(2-Bromophenyl)­ethanol **1f** to Dihydrobenzopyran **3fa’**

Then, replacing bromobenzene with iodobenzene **6** resulted in moderate yields for compound **3aa** and very good yields for **3ab**. Even if it is reported that iodobenzene^92^ is slightly more reactive than bromobenzene, its use with deactivated phenols such as **2c**–**e** and **2k** did not lead to product formation and detection (Table 6, entries 3–6).

**Table 6:**
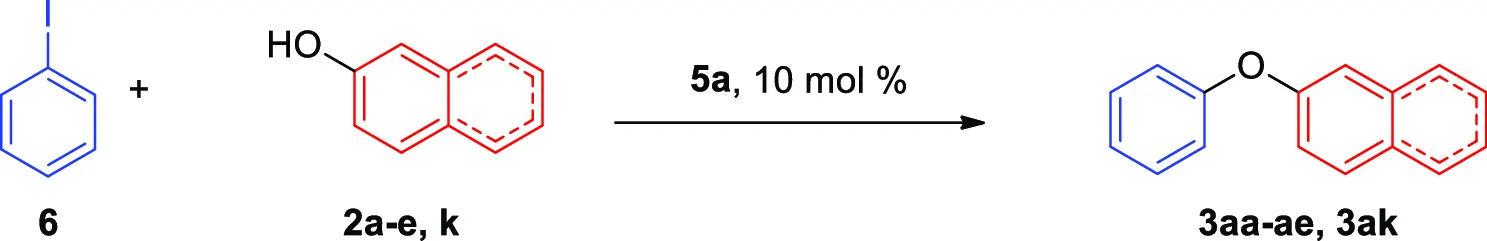

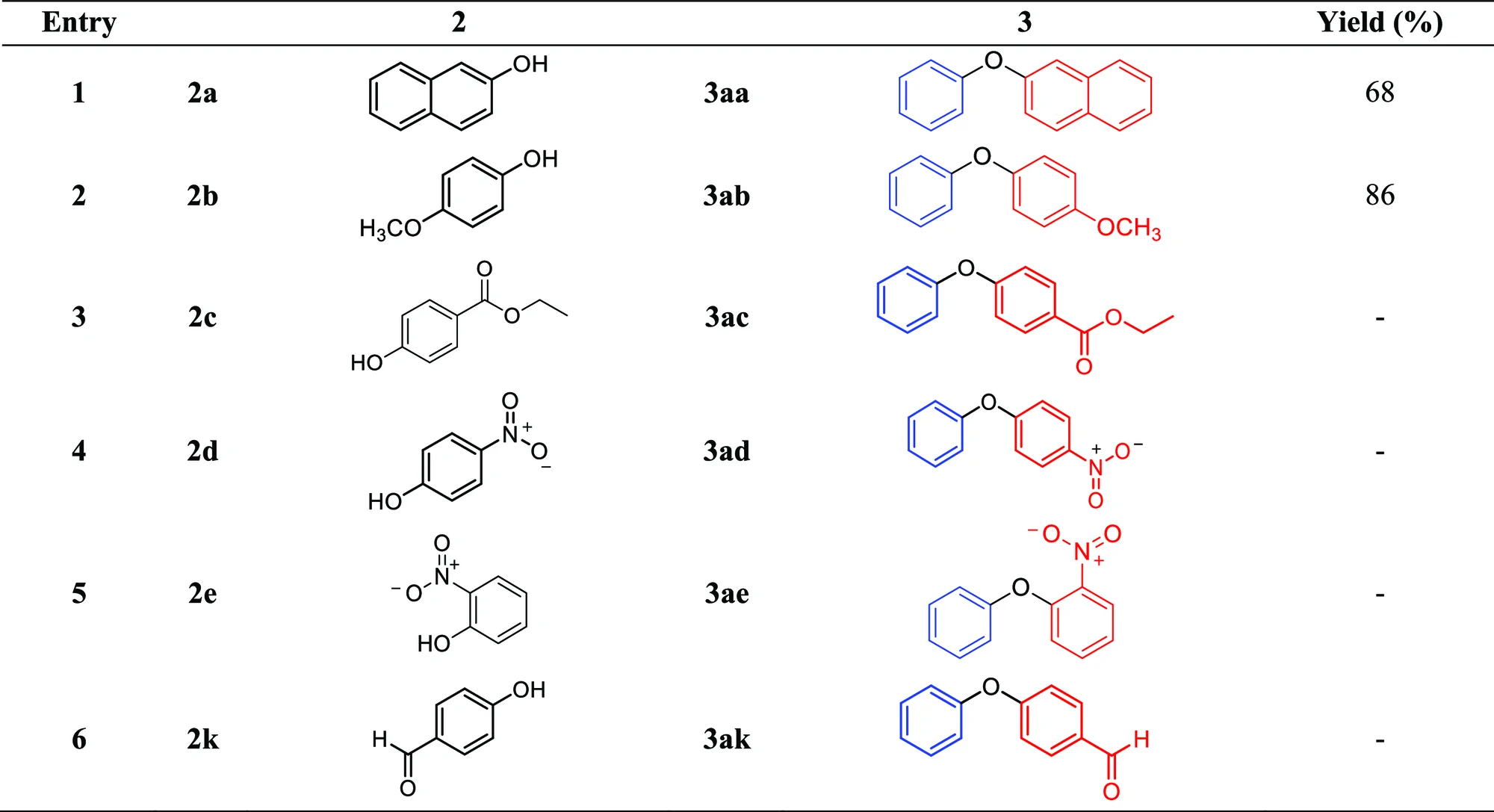
Ullmann’s Coupling of Iodobenzene 6^a^

a Typical reaction conditions: 1 mmol of **1**, 1.5 mmol of **2**, 2.0 mmol of Cs_2_CO_3_, ACN (1.0 M), 120 °C, μW irradiation, 3.5 h reaction time.

The reaction works well when activated bromobenzenes and deactivated phenols are used, but yields are higher when both reagents bear an activated aromatic ring (Table 7, entries 1–3).

**Table 7:**
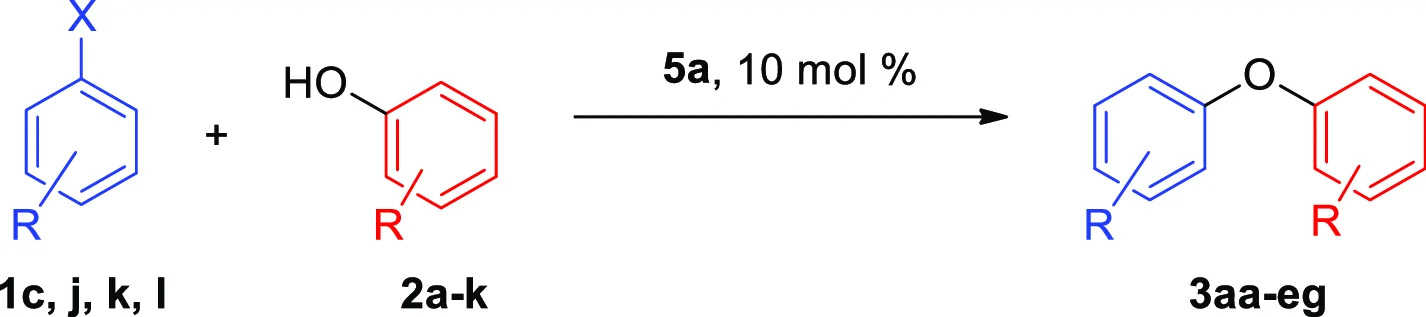

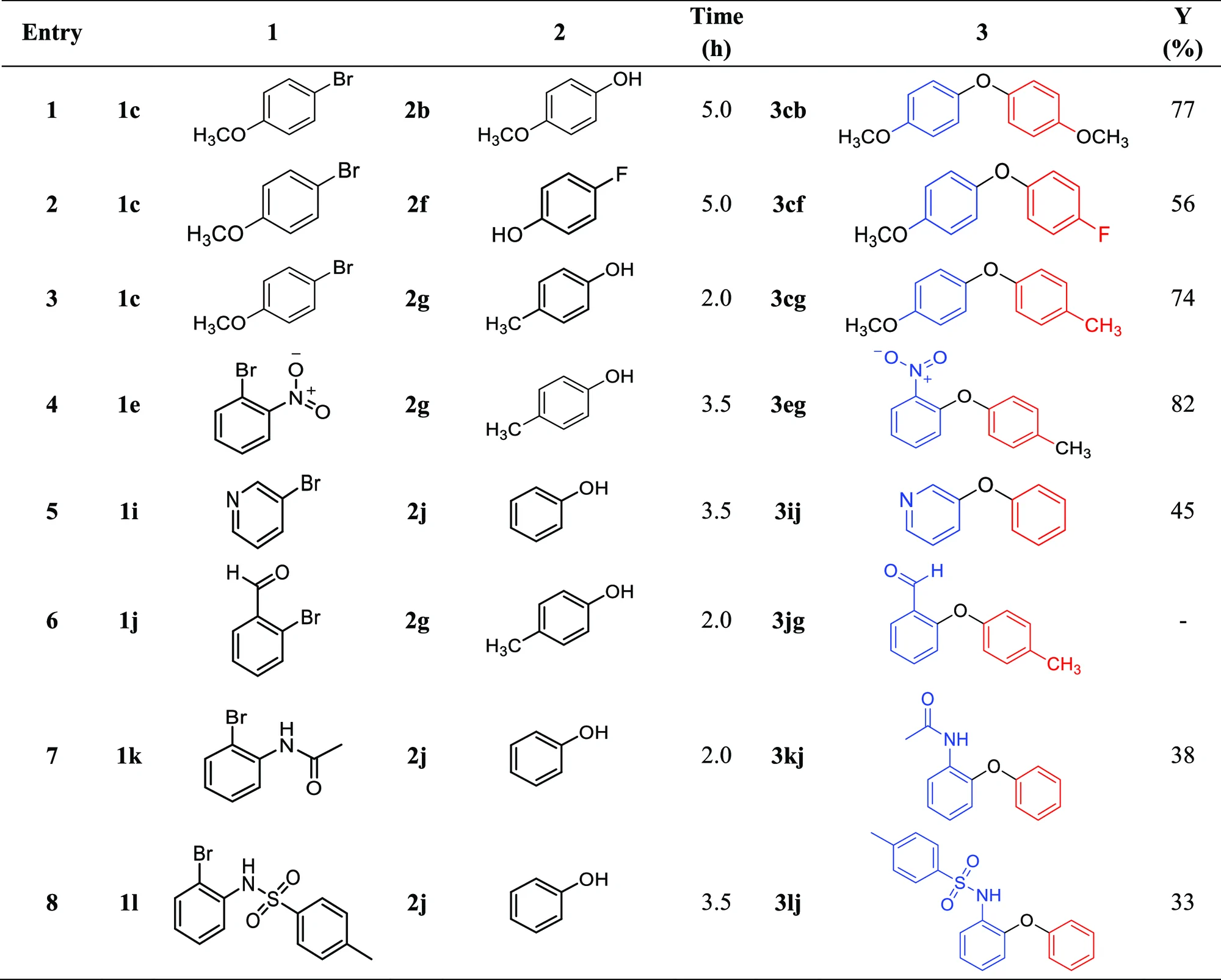
Ullmann’s Coupling of Substituted Bromobenzenes^a^

a Note: Typical reaction conditions: 1 mmol of **1**, 1.5 mmol of **2**, 2.0 mmol of Cs_2_CO_3_, ACN (1.0 M), 120 °C, μW irradiation, 3.5 h reaction time.

In addition, compound **3cf** is an intermediate in the total synthesis of XB-1 analogues known to be novel histamine H_3_ receptor antagonists.^93^ The incorporation of a heterocycle **1i** adversely impacted product formation **3ij** (Table 7, entry 5). This is likely due to the coordination of the nitrogen atom with the copper center.^94^ Unfortunately, when reagent **1j** was employed, no product was obtained (Table 7, entry 6), confirming the limited activity of aldehydes, probably due to their instability under the reaction conditions. The combination of electron-withdrawing groups on the aryl halide and electron-donating groups on the phenol was highly effective, producing a very high yield of diphenyl ether **3eg** (Table 7, entry 4). Finally, the reactivity of the two protected 2-bromoanilines, **1k** and **1l,** was also evaluated. The reaction with **1l** was ineffective (Table 7, entry 8), resulting in the recovery of a large amount of the starting material, no trace of unprotected **3lj,** and a low yield of product **3lj**. Fascinating results were obtained with *N*-(2-bromophenyl)­acetamide **1k** because two distinct products were isolated, arising from competition between intramolecular and intermolecular reaction pathways. Product **3kj** was obtained through the standard intermolecular Ullmann coupling studied in this paper (Table 7, entry 7), while benzoxazole **3kj’** was formed with a yield of 40% by an intramolecular route, as just reported in the literature under similar reaction conditions^95^ and depicted in Scheme 3.

**Scheme 3:**
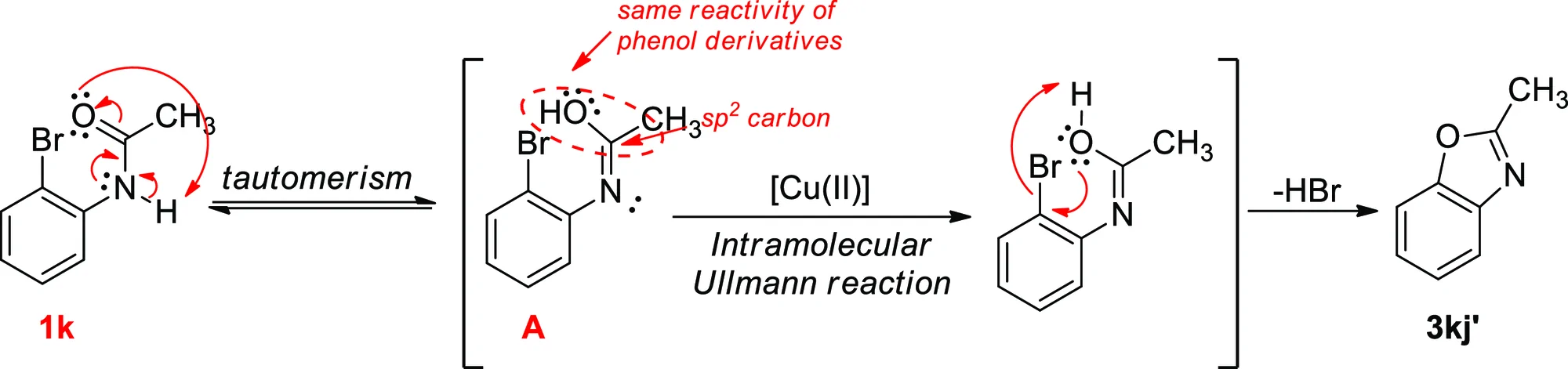
Mechanism Hypothesis for the Formation of Product **3kj’**

The starting material **1k** undergoes a tautomeric equilibrium at the amide moiety, forming the enol intermediate **A,** which can explain the phenol-like reactivity. Once formed, **A** undergoes an intramolecular Ullmann reaction, leading to the formation of benzoxazole **3kj’**, in a yield comparable to that of **3kj**.

## Conclusions

In this study, a microwave-assisted copper­(II)-catalyzed Ullmann-type C–O coupling protocol was developed and optimized for the efficient synthesis of a wide range of diaryl ethers. β-Diketonate ligands bearing different structural motifs enabled fine-tuning of the catalytic activity of the corresponding Cu­(II) complexes. The aim of the present study is to demonstrate that Cu­(II) β-diketonate complexes represent an effective catalyst platform for Ullmann-type C–O bond formation under microwave irradiation. These results support the view that Cu­(II)-based catalytic systems deserve further attention in microwave-assisted Ullmann C–O coupling and may complement the more established Cu­(I)-based methodologies.

Among them, complex **5a** is the most efficient system, consistently providing high yields of diaryl ether products under mild and rapid microwave conditions. The synthetic protocol was demonstrated to be general and tolerant toward a broad range of substrates, including both electron-rich and electron-poor phenols and aryl halides, revealing notable trends in reactivity and selectivity. The reaction was shown to proceed via a nonradical pathway, as demonstrated by control experiments in the presence of radical scavengers. From a synthetic point of view, the use of newly synthesized Cu­(II) complexes such as **5f**, as well as the previously characterized **5b**–**d**, allowed us to explore how steric and electronic properties of ligands can influence the catalytic performance. In conclusion, this microwave-assisted Ullmann-type reaction represents a practical and faster alternative to conventional thermal methods, providing shorter reaction times and high efficiencies under the investigated conditions. The short reaction times, high selectivity, and applicability to a variety of substrates make this methodology highly attractive for the synthesis of bioactive diaryl ether scaffolds and for further development of copper-catalyzed organic synthesis.

## General Methods

Here is the general method for synthesizing diaryl ethers. In a microwave vial, aryl halides **1a**–**h** and **6** (1 mmol, 1 equiv), phenol derivative **2a**–**k** (1.5 eq., 1.5 mmol), Cs_2_CO_3_ (2.0 mmol, 2.0 equiv), and copper complex **5a**–**h** (0.1 mmol, 0.1 equiv) were weighed. The vial was then placed in the microwave reactor, and the reaction was heated to 120 °C. The reaction progress was monitored by thin-layer chromatography (9:1 Hexane/EtOAc) and gas chromatography. Once the starting material was consumed, the reaction mixture was extracted with EtOAc (20 mL) and brine (20 mL). The two layers were separated, and the aqueous phase was washed twice with fresh EtOAc (2 × 20 mL). The combined organic layers were dried over anhydrous Na_2_SO_4_, filtered, and the solvent was evaporated under reduced pressure using a Rotavapor. The resulting crude product was purified by silica gel column chromatography (gradient from 99:1 to 80:20 Hexane/EtOAc).

## Supplementary Material



## Notes

General information, reaction conditions, materials, full characterization by FT-IR, MS, and melting point data, and ^1^H, ^13^C, ^19^F are reported. All reagents used for the synthesis of ligands and complexes were purchased from Merck and used without further purification (PDF)
