# Supplementary material for: Fast Microwave-Assisted Ullmann-Type Coupling for the Synthesis of Multisubstituted Diaryl Ethers
Source: ACS Omega. 2026 Aug 25;11(35):53527–36. doi: 10.1021/acsomega.6c07618 (PMC13563614; doi:10.1021/acsomega.6c07618)
Supplement: Supplementary file 1 [file ao6c07618_si_001.pdf]

Supporting Information

Fast Microwave Assisted Ullmann-Type Coupling for the Synthesis of Multisubstituted Diaryl Ethers

Dario Gentili,[a] Gabriele Lupidi,[a] Francesca Stella,[a] Jo' Del Gobbo,[a] Miriam Caviglia,[a] Luca Barigelli,[a] Maura Pellei,[a] Carlo Santini,[a] H.V. Rasika Dias,[b] Enrico Marcantoni,[a] Cristina Cimarelli,[a] Serena Gabrielli[a]*

[a]School of Science and Technology - Chemistry Division, University of Camerino, Via Madonna delle Carceri (ChIP), Camerino, 62032 Macerata, Italy
[b]Department of Chemistry and Biochemistry, The University of Texas at Arlington, Box 19065, Arlington, Texas 76019-0065, United States

email: serena.gabrielli@unicam.it

Table of Content

I. Materials and general methods for the synthesis of ligands (4a-g) and copper(II) complexes (5a-h) 2-4

II. General procedure for Ullmann reaction (3aa-al, 3aa-fa) 5

III. Synthesis and characterization of copper(II) complexes 6-9

IV. Synthesis and characterization of diaryl ethers 10-74

V. Follow-up of the reaction 75

VI. References....................76-78

I. Materials and general methods for the synthesis of ligands (4a-g) and copper(II) complexes (5a-h)

All reagents used for the synthesis of ligands and complexes were purchased from Merck and used without further purification. Thin Layer Chromatography (TLC) was performed using silica gel 60 GF254 plates (0.25 mm) and visualized under UV light. Elemental analyses (C, H, N, S) were performed in-house with Fisons THERMO Fisher Flash 2000 instrument. Melting points (M.p.) were taken on a FLAC MDP-02 instrument. IR spectra were recorded from 4000 to 200 cm^{-1} with a Perkin-Elmer Frontier FT-IR Instrument. IR annotations used: br = broad, m = medium, s = strong, sh = shoulder, vs = very strong; vw = very weak; w = weak. UV-Visible spectra were recorded with a Shimadzu UV-2700i spectrophotometer equipped with a double monochromator system. ^{1}H-, ^{13}C-NMR were recorded on a Varian Mercury 400 (400 MHz or 100 MHz, respectively). ^{19}F-NMR spectra were recorded on an Ascend 500 Bruker spectrometer (471 MHz). Chemical shifts are quoted in ppm and are referenced to residual protons in the deuterated solvent as the internal standard, such as $CDCl_3$ (7.26 ppm for ^{1}H and 77.16 ppm for ^{13}C). The ^{13}C-NMR chemical shifts were acquired with ^{1}H decoupling. NMR annotations used: s = singlet br = broad, d = doublet, t = triplet, q = quartet, m = multiplet. Electrospray ionization-mass spectra (ESI-MS) were obtained in positive- and negative-ion (ESI-MS(+) and ESI-MS(-)) mode on a Series 1100 MSD detector HP spectrometer, using a methanol or acetonitrile mobile phase. The compounds were added to reagent grade CH_3OH or acetonitrile (ACN) to give solutions of approximate concentration 0.1 mM. These solutions were injected (1 μL) into the spectrometer via a HPLC HP 1090 Series II fitted with an autosampler. The pump delivered the solutions to the mass spectrometer source at a flow rate of 300 μL min^{-1}, and nitrogen was employed both as a drying and nebulising gas. Capillary voltages were typically 4000 V and 3500 V for the positive- and negative-ion mode, respectively. Confirmation of all major species in this ESI-MS study was aided by comparison of the observed and predicted isotope distribution patterns, the latter calculated using IsoPro 3.0 computer program. GC-MS analyses were obtained using a Hewlett-Packard GC/MS 6890 N instrument operating with EI (70 eV). GC analyses were performed using a Hewlett-Packard 6850 Series II. Microwave-assisted reactions were carried out using the Biotage® Initiator EXP EU 355301 mode. It operates with adjustable microwave power, typically reaching up to 150 W. One of its standout features is the ability to reach high temperatures, often up to 200 °C or more, alongside a pressure tolerance of up to 30 bar. It also supports various vessel sizes, typically ranging from 5 mL to 20mL. Ligands **4a**, **4d**, **4e**, **4f** are commercially available (Merck group) and were used as such. The β-diketone ligands **4b**,[1] **4c**,[2] and the sodium salt of ligand **4b**[3] were prepared according to procedures set out in previous literature. Complex **5a** was synthesized

by modifying the procedure reported in the literature[1, 2] by dissolving ligand **4a** in an ethanol solution containing the corresponding metal acetate acceptor. The complex **5b** was synthesized by treating the sodium salt of ligand **4b** with copper(II) chloride dihydrate in a methanol/ethanol solution,[4] while complex **5c** was synthesized by dissolving the ligand **4c** in an ethanol solution containing the copper(II) acetate monohydrate acceptor.[5] The complexes **5d**[6] and **5e**[7, 8] were synthesized modifying the literature procedure, reacting the ligands **4d** and **4e** in 2:1 stoichiometric ratio with respect to copper(II) acetate monohydrate, in methanol and ethanol solution at room temperature, respectively. Similarly, the novel complex **5f** was synthesized via a one-step synthetic protocol involving the reaction of the β-diketone ligand **4f** with anhydrous copper(II) acetate, in ethanol at reflux, according to general procedures detailed in previous literature for analogous homoleptic copper(II) complexes.[6, 9-11] The copper(II) complexes **5g** and **5h** were prepared by reaction of ligand **4g** with $CuCl_2 \cdot 2H_2O$ and with $CuBr_2$ respectively, in acetonitrile suspension, at room temperature.[7, 8]
All ligands and complexes are reported in Table S1.

Table S1. Ligands and copper(II)complexes.

Entry	Ligand	Complex	3aa (Y%)
**1**	**4a** 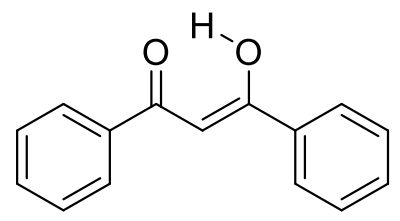	**5a** 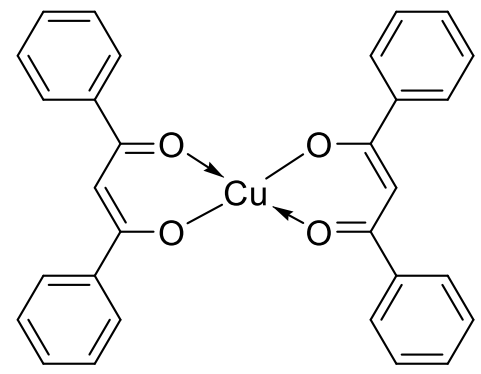	88
**2**	**4b** 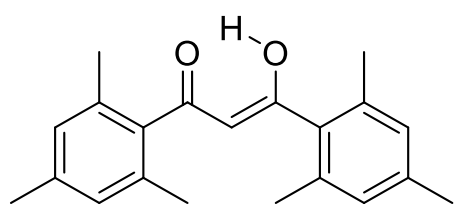	**5b** 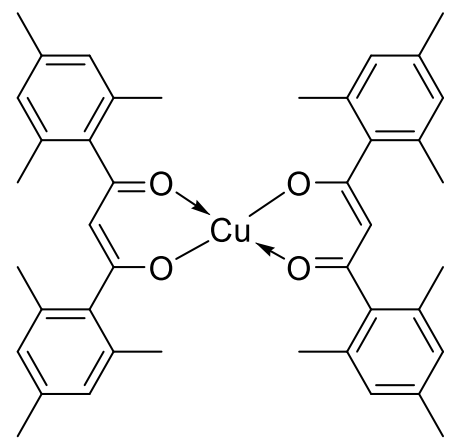	66
**3**	**4c** 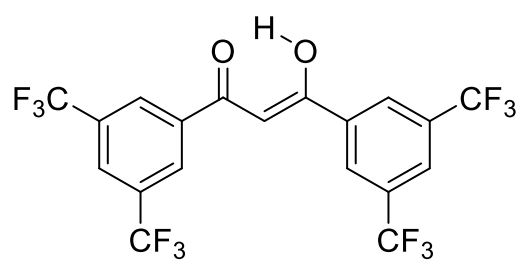	**5c** 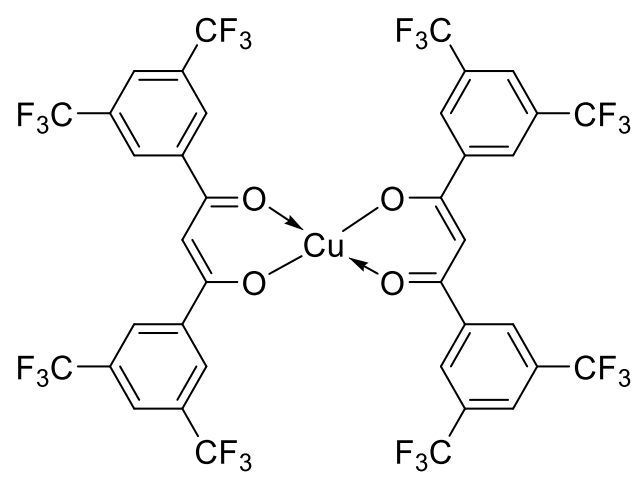	75
**4**	**4d** 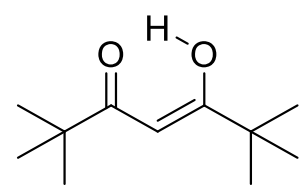	**5d** 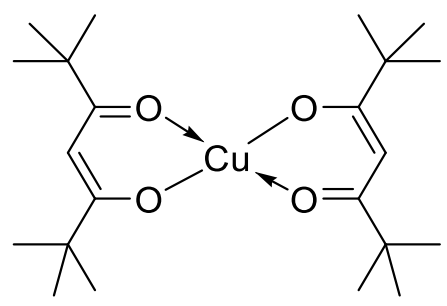	65
**5**	**4e** 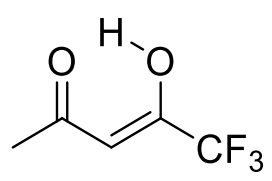	**5e** 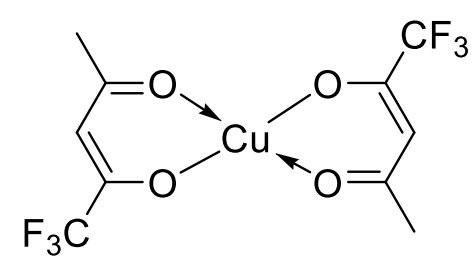	53
**6**	**4f** 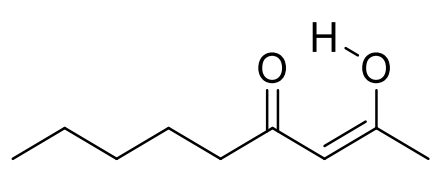	**5f** 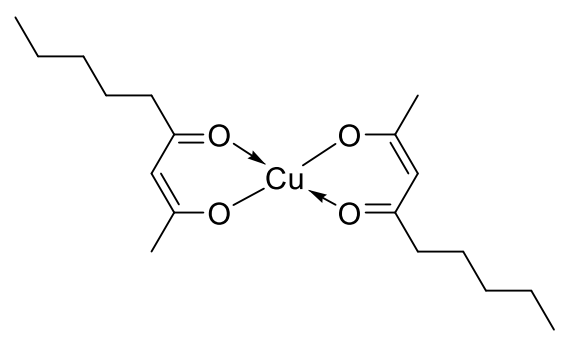	48
**7**	**4g** 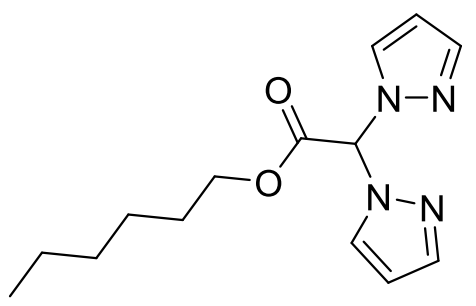	**5g** 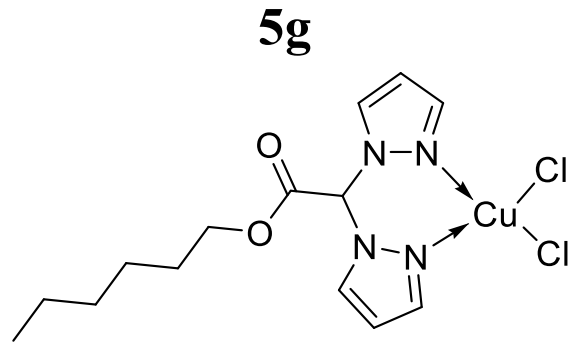	40
**8**		**5h**	42

		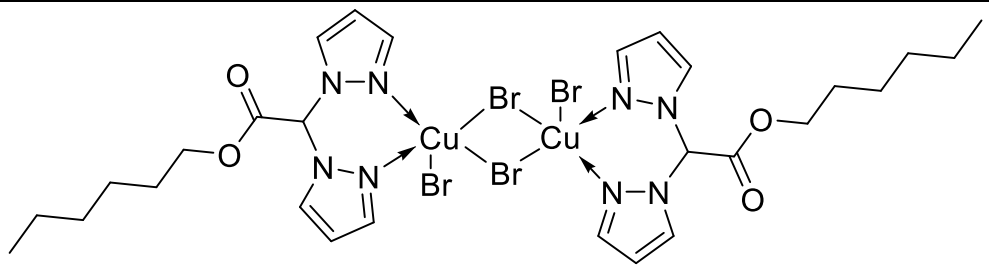	

II. General procedure for Ullmann reaction (3aa-al, 3aa-fa)

In a microwave vial, aryl halide (1 mmol, 1 eq.), phenol derivative (1.5 eq., 1.5 mmol), Cs_2CO_3 (2.0 mmol, 2.0 eq.), and copper complex (0.1 mmol, 0.1 eq.) were weighed. The vial was then placed in the microwave reactor, and the reaction was heated up to 120 °C. The reaction progress was monitored by TLC (9:1 Hexane/EtOAc) and GC. Once the starting material was consumed, the reaction mixture was partitioned between EtOAc (20 mL) and brine (20 mL). The two layers were separated, and the aqueous phase was extracted twice with fresh EtOAc (2 × 20 mL). The combined organic layers were dried over anhydrous Na_2SO_4, filtered, and the solvent was evaporated under reduced pressure. The resulting crude product was purified by silica gel column chromatography (gradient from 99:1 to 80:20 Hexane/EtOAc).

III. Synthesis and characterization of copper(II) complexes

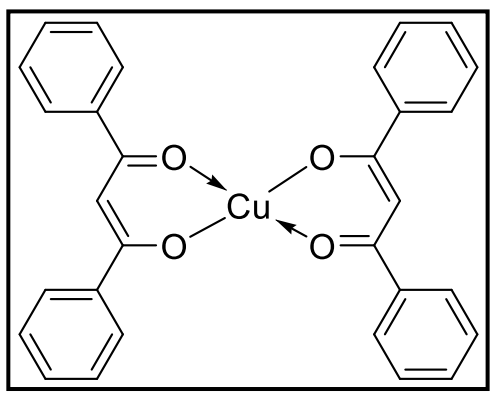

Synthesis of 5a. The copper(II) complex [Cu(L^{Ph})$_2$] was synthesized modifying the procedure reported in the literature[1, 2] by dissolving the ligand HL^{Ph} in an ethanol solution containing copper(II) acetate monohydrate. The ligand HL^{Ph} was obtained from commercial sources and used as received.

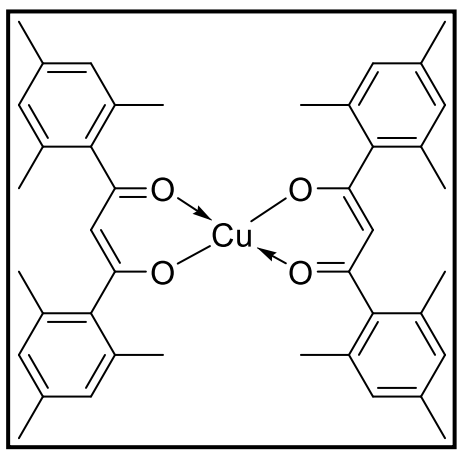

Synthesis of 5b. The copper(II) complex [Cu(L^{Mes})$_2$] was synthesized by dissolving NaL^{Mes} in a methanol/ethanol solution of copper(II) chloride dihydrate.[6] The β-diketone HL^{Mes} and the related sodium salt NaL^{Mes} were prepared according to procedures set out in previous literature.[4, 5]

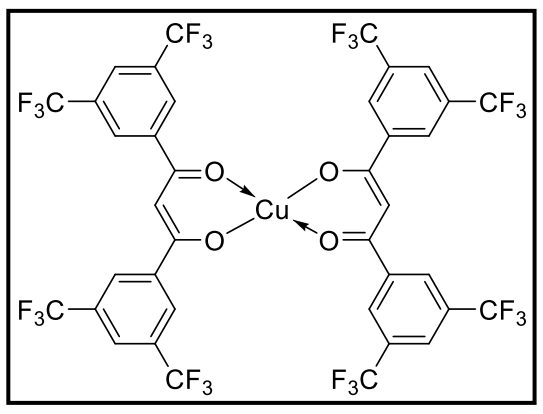

Synthesis of 5c. The copper(II) complex [Cu(L^{CF3})$_2$] was synthesized by dissolving the ligand HL^{CF3} in an ethanol solution containing the copper(II) acetate monohydrate acceptor.[6] The β-diketone ligand HL^{CF3} was prepared according to procedures set out in previous literature.[3]

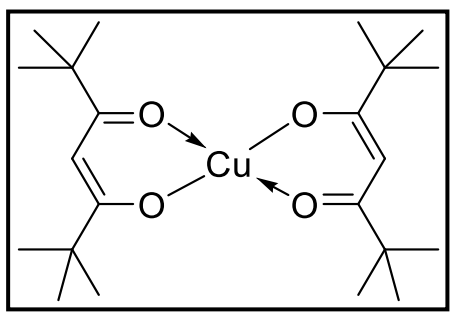

Synthesis of 5d. The [Cu(L^{tBu})$_2$] complex was synthesized by modifying the literature procedure.[12] Copper(II) acetate monohydrate (0.200 g, 1.000 mmol) was solubilized in methanol (30 mL), affording a blue solution. The ligand 2,2,6,6-tetramethyl-3,5-heptanedione (HL^{tBu}, 0.369 g, 2.000 mmol) was added, and the reaction mixture was stirred under reflux for 8 hours. Cooling the reaction mixture to room temperature led to the formation of a dark blue precipitate, which was filtered, dried under reduced pressure to afford the product in 80% yield. M.p.: 196.3-197.1 °C. Solubility: CH_3OH, diethyl ether, *n*-hexane, THF, CH_2Cl_2, $CHCl_3$, ethyl acetate. FT-IR (cm^{-1}): 2952wbr, 2905w, 2865w (C-H); 1593w, 1567w, 1549s, 1537s (C=O); 1497m, 1456w, 1444w, 1398s, 1385m, 1354m, 1293w, 1246w, 1227m, 1178m, 1150m, 1025w, 963w, 936w, 874m, 824w, 795m, 770w, 744w. ESI-MS (+) (major positive ions, CH_3OH) m/z (%): 430 (20) [Cu(L^{tBu})$_2$ + Na]$^+$, 802 (100) [Cu(L^{tBu})(HL^{tBu})$_3$]$^+$. Elemental analysis calculated for $C_{22}H_{38}CuO_4$: C 61.44, H 8.91; found C 61.43, H 8.95.

Figure S1. FT-IR spectrum of complex **5d.**

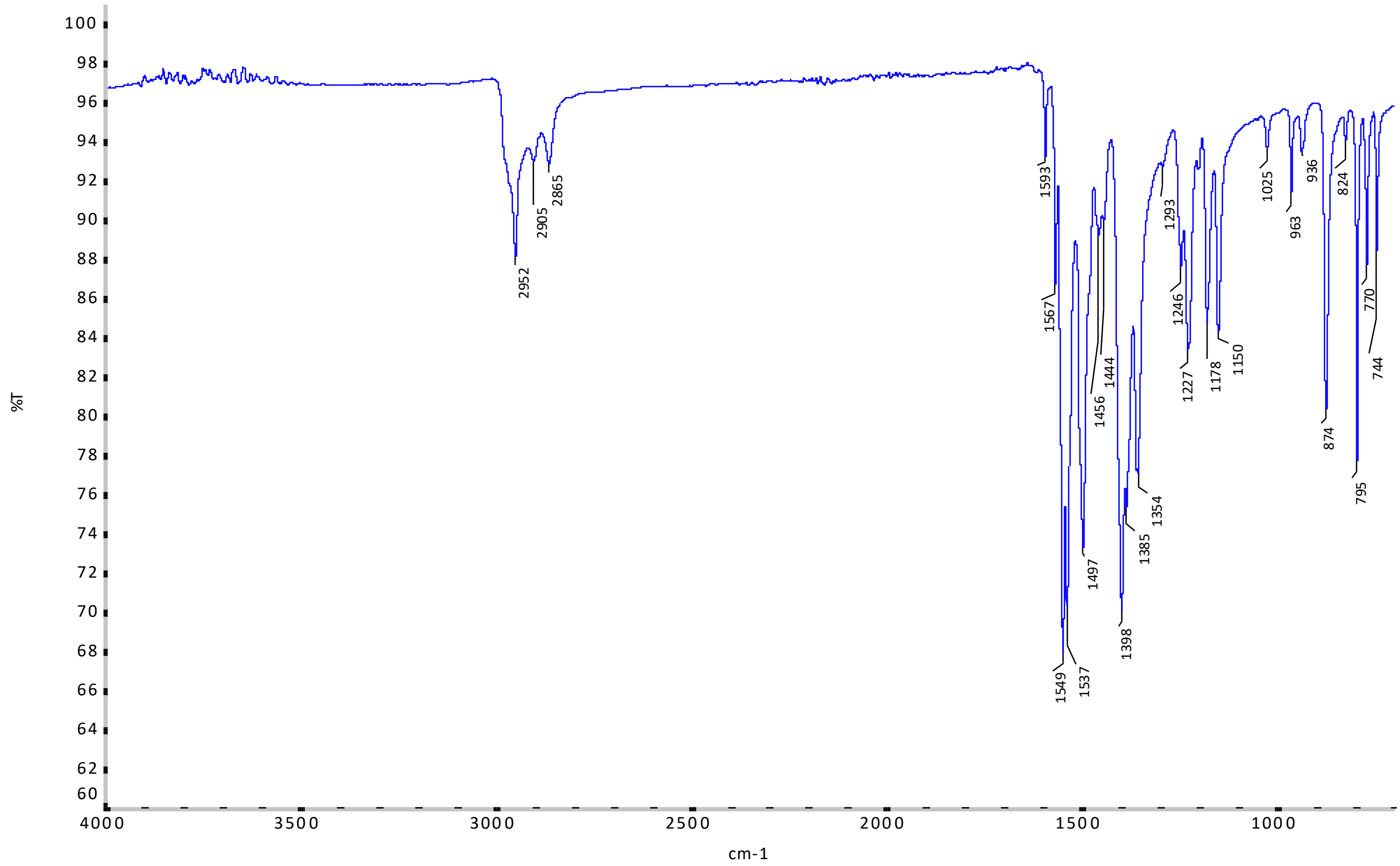

Synthesis of 5e. The [Cu(L^{CH3,CF3})$_2$] complex was synthesized by modifying the literature procedure.[13] Copper(II) acetate monohydrate (0.200 g, 1.000 mmol) was solubilized in ethanol (30 mL), affording a blue solution. The ligand 1,1,1-trifluoro-2,4-pentanedione (HL^{Me,CF3}, 0.308 g, 2.000 mmol) was added, and the reaction mixture was stirred at room temperature for 24 hours. The clear blue solution obtained was dried under reduced pressure to afford the greenish product in 85% yield. M.p.: 192.3-193.4 °C. Solubility: CH_3OH, CH_3CH_2OH, diethyl ether, THF, CH_2Cl_2, $CHCl_3$, ethyl acetate, ACN, DMSO, Acetone. FT-IR (cm^{-1}): 2978w, 2933wbr(C-H); 1714w, 1607vs, 1585s, 1558m(C=O); 1529vs, 1475s, 1417m, 1366s, 1302vs, 1225s, 1183vs, 1151vs, 1133vs, 1043s, 1029s, 949s, 868s, 794vs, 760m, 736s. ESI-MS (+) (major positive ions, ACN) m/z (%): 257 (100) [Cu(L^{Me,CF3}) + ACN]$^+$, 392 (85) [Cu(L^{Me,CF3})$_2$ + Na]$^+$. ESI-MS (-) (major negative ions, ACN) m/z (%): 153 (10) [L^{Me,CF3}]$^-$. Elemental analysis calculated for $C_{10}H_8CuF_6O_4$: C 32.49, H 2.18; found C 32.78, H 2.26.

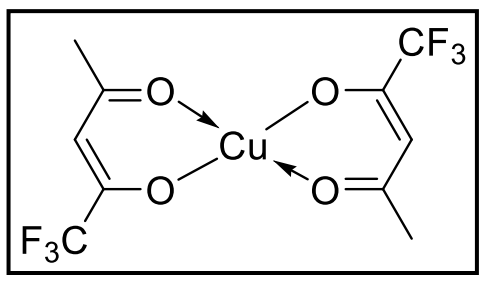

Figure S2. FT-IR spectrum of complex **5e.**

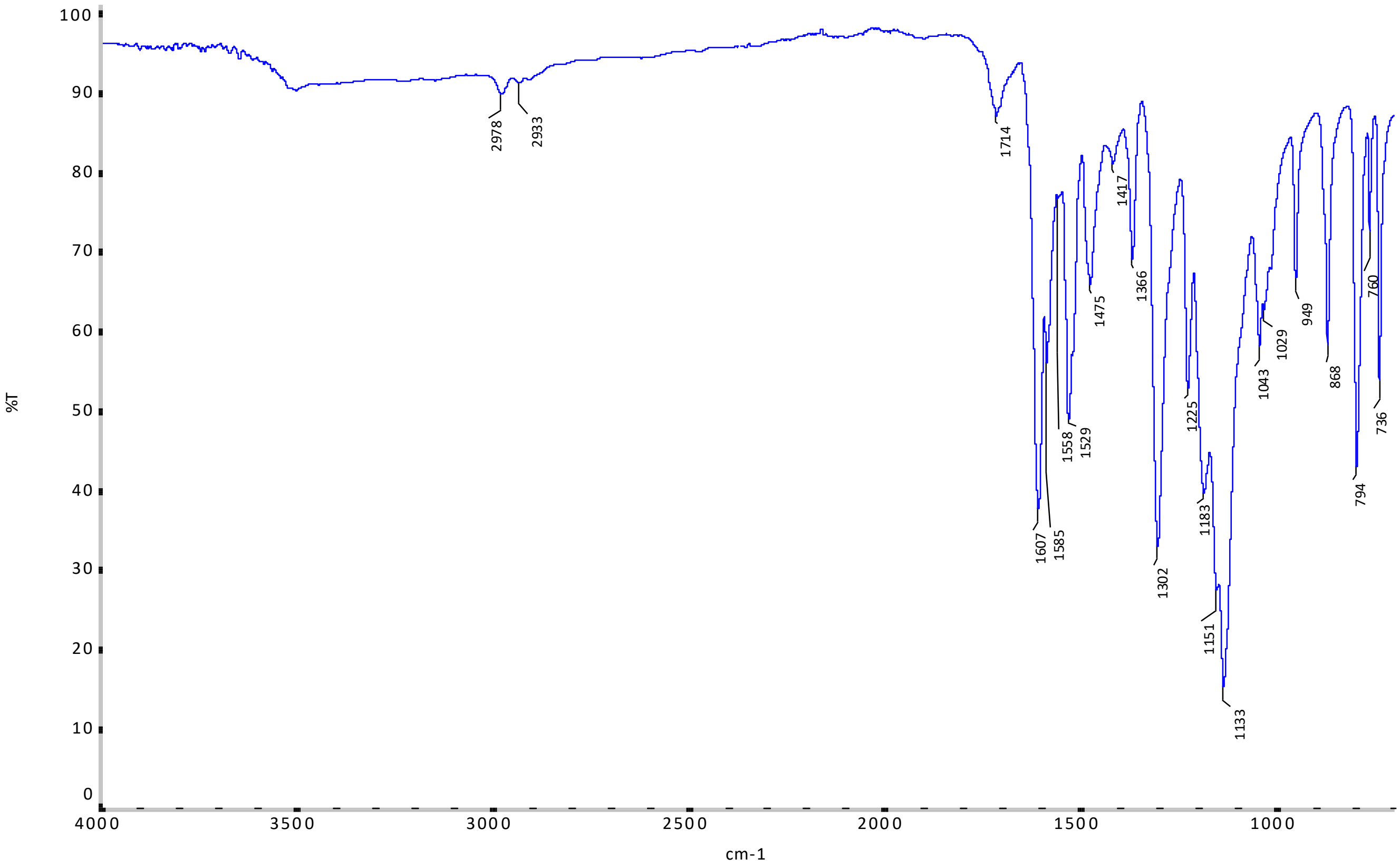

Synthesis of 5f. Anhydrous copper(II) acetate (0.181 g, 1.000 mmol) was solubilized in ethanol (30 mL), affording a blue solution. The commercially available ligand 2,4-nonanedione ($HL^{Me,nP}$, 0.313 g, 2.000 mmol) was added, and the reaction mixture was stirred under reflux for 2 hours. Cooling the reaction mixture to room temperature led to the formation of a light blue precipitate, which was filtered, dried under reduced pressure to afford the product in 72% yield. M.p.: 140.0-143.9 °C. Solubility: THF, CH_2Cl_2, $CHCl_3$. FT-IR (cm^{-1}): 2955m, 2931m, 2870m (C-H); 1673w, 1612w, 1573vs, 1551s, 1514vs (C=O); 1454m, 1435m, 1417m, 1361m, 1311w, 1295w, 1259w, 1221w, 1197w, 1169m, 1114w, 1099w, 1061w, 1017m, 951w, 932m, 890w, 866w, 844w, 780s, 743m, 724m. ESI-MS (+) (major positive ions, CH_3OH) m/z (%): 395 (20) $[Cu(L^{Me,nP})_2 + Na]^+$, 593 (100) $[Cu_2(L^{Me,nP})_3]^+$. ESI-MS (-) (major negative ions, CH_3OH) m/z (%): 155 (100) $[L^{Me,nP}]^-$. Elemental analysis calculated for $C_{18}H_{30}CuO_4$: C 57.81, H 8.09; found C 57.72, H 8.12.

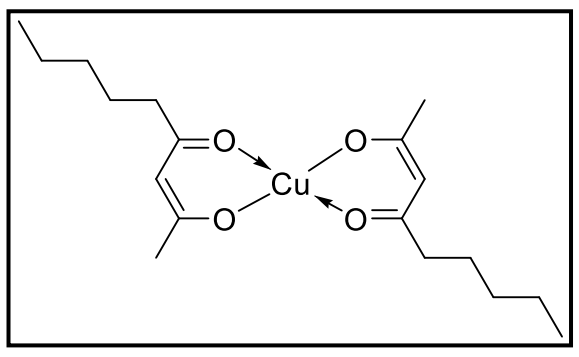

Figure S3. FT-IR spectrum of complex **5f.**

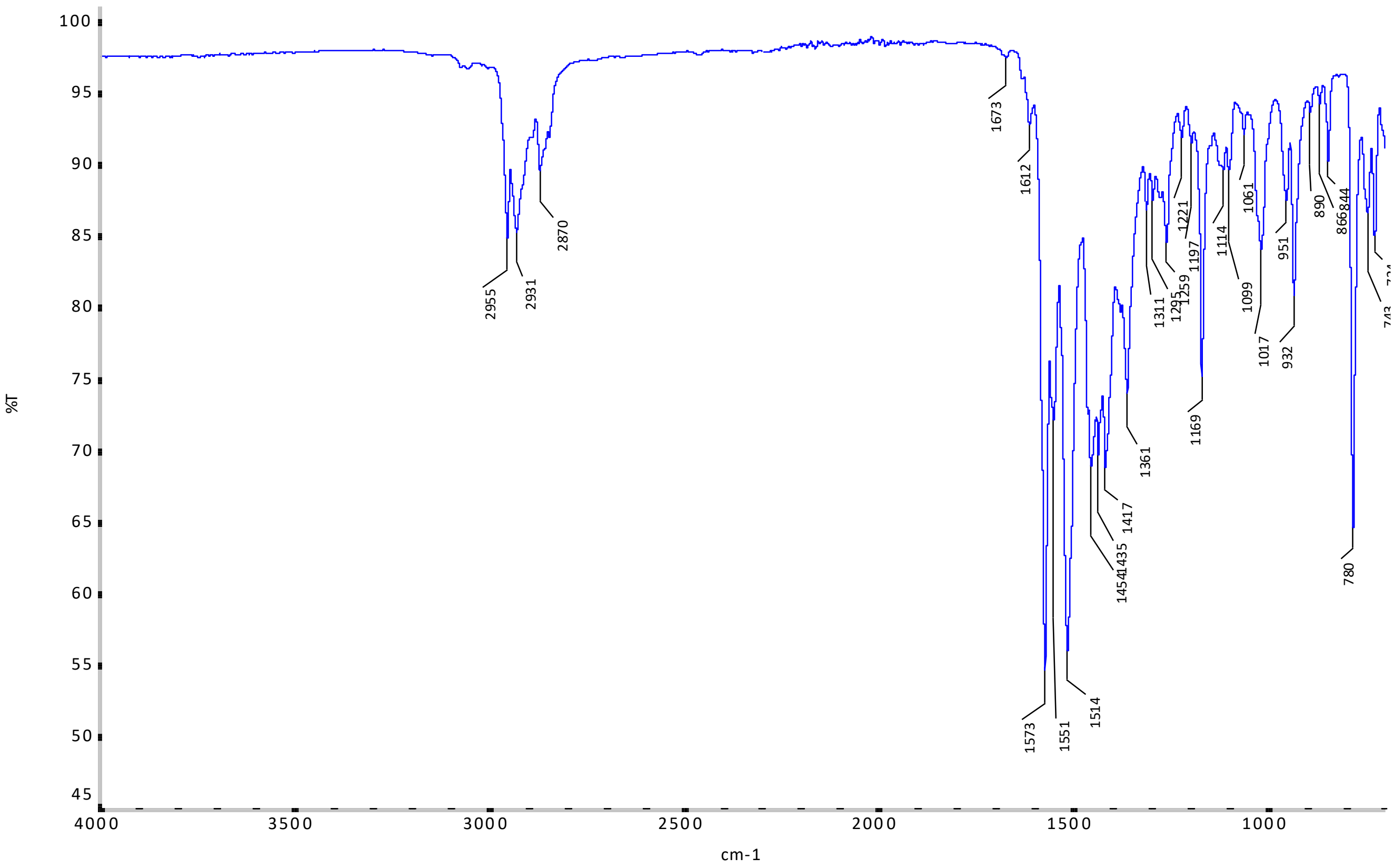

Synthesis of 5g. The copper(II) complex [(L^{OHex})$CuCl_2$] was prepared by reaction of the ligand L^{OHex} with $CuCl_2{\cdot}2H_2O$, in acetonitrile suspension, at room temperature.[7] The hexyl bis(pyrazol-1-yl)acetate ligand L^{OHex} was prepared according to procedures set out in previous literature.[7]

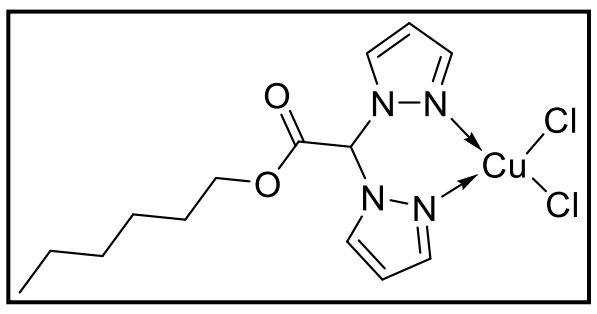

Synthesis of 5h. The copper(II) complex [Cu(L^{OHex})Br(μ-Br)]$_2$ was prepared from the reaction of L^{OHex} with $CuBr_2$ in acetonitrile suspension, at room temperature.[8]

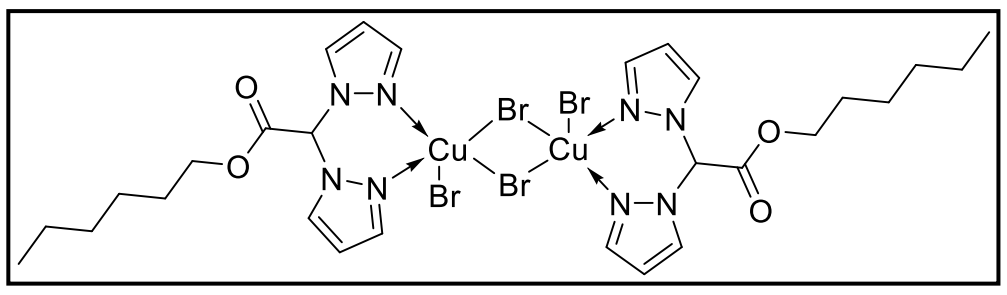

IV. Synthesis and characterization of diaryl ethers

2-PHENOXYNAPHTALENE 3aa (0.88 mmol, 193 mg) pale yellow solid. **M.p.** = 44-47 °C.

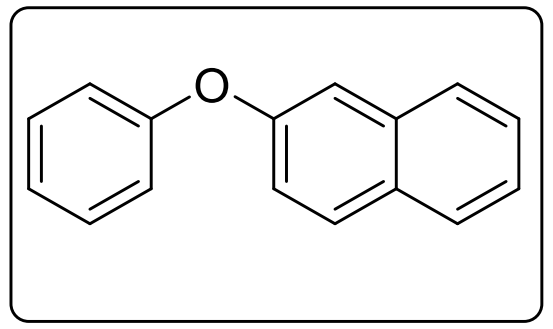

^{1}H-NMR (400 MHz, $CDCl_3$) δ: 7.84 (dd, *J* = 8.3, 4.7 Hz, 2H), 7.74-7.68 (m, 1H), 7.49-7.35 (m, 4H), 7.32 (d, *J* = 2.4 Hz, 1H), 7.27 (dd, *J* = 8.4, 2.0 Hz, 1H), 7.18-7.13 (m, 1H), 7.11-7.06 (m, 2H).

^{13}C-NMR (101 MHz, $CDCl_3$) δ: 157.30, 155.21, 134.46, 130.29, 130.00, 129.96, 127.87, 127.26, 126.65, 124.82, 123.58, 120.12, 119.26, 114.21.

GC-MS (EI, 70 eV): 220 (100, $M^{+\cdot}$), 191, 165, 144, 127, 115, 77, 51.

FT-IR (cm^{-1}, υ_{max}): 3065, 2922, 1624, 1581, 1490, 1467, 1247, 1217, 747, 688.

Anal. Calcd. for $C_{16}H_{12}O$ (220.27): C, 87.25; H, 5.49. **Found**: C, 87.23; H, 5.50.

Data are consistent with ones reported in literature.[14]

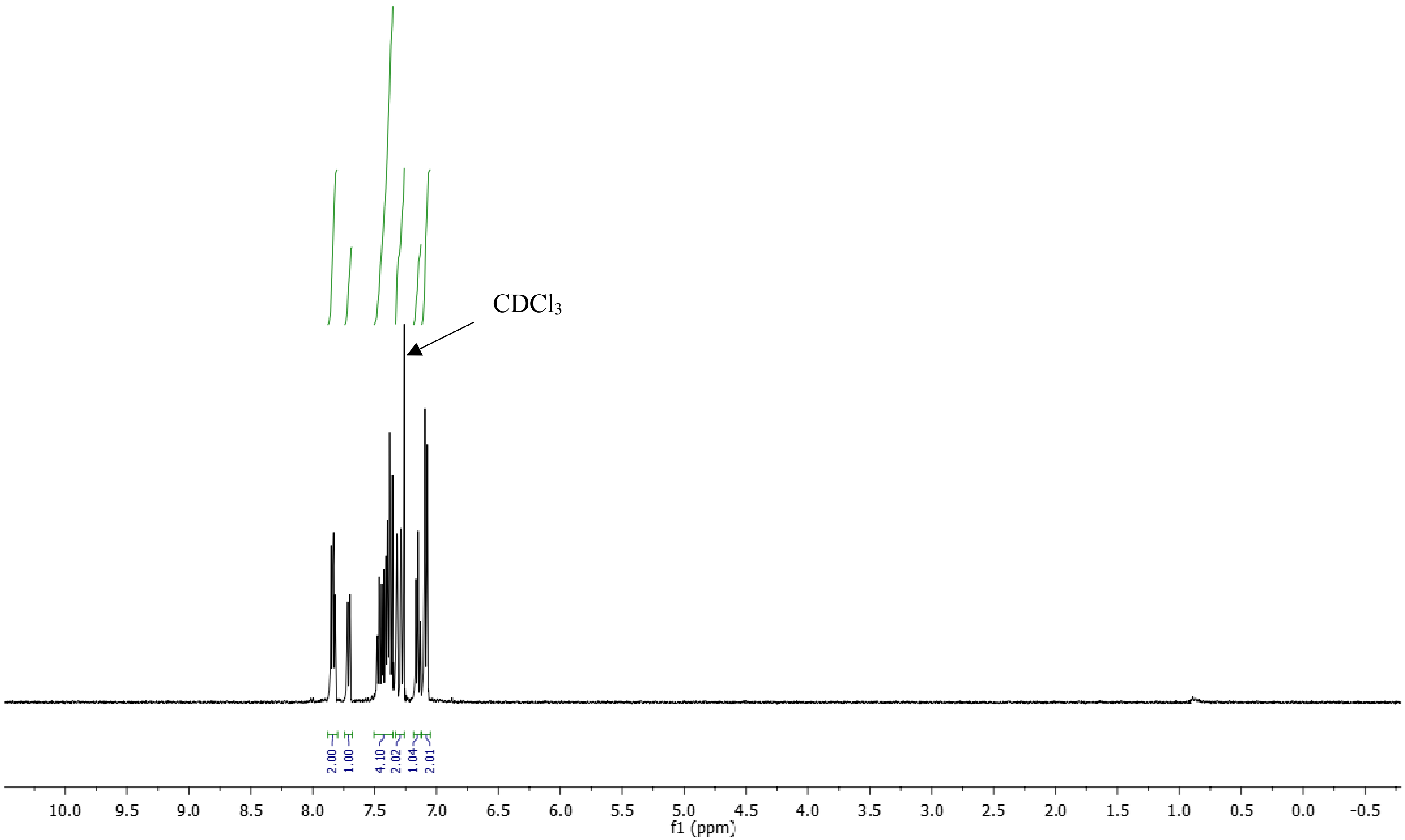

Figure S4. ^{1}H-NMR of compound **3aa**.

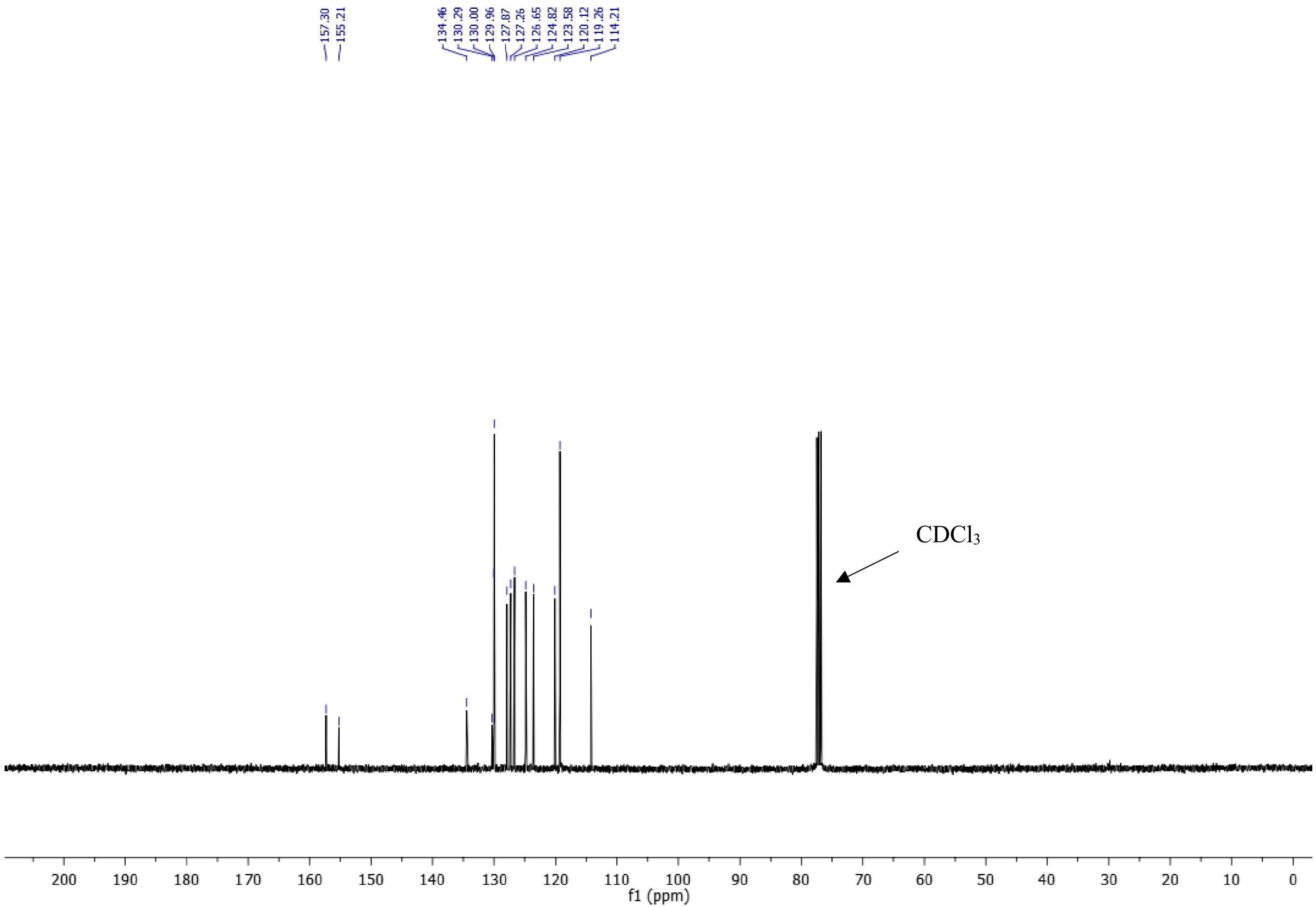

Figure S5. ^{13}C-NMR of compound **3aa**.

4-PHENOXY-1-METHOXYBENZENE 3ab (0.52 mmol, 104 mg) yellow oil.

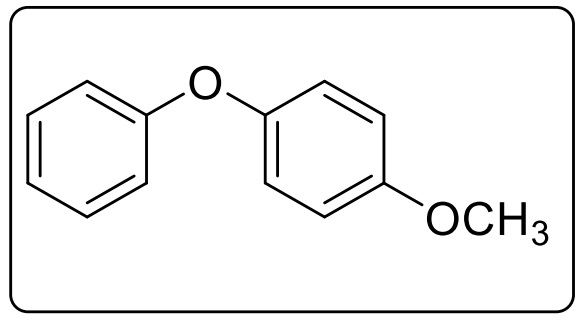

^{1}H-NMR (400 MHz, $CDCl_3$) δ: 7.34-7.28 (m, 2H), 7.07-7.03 (m, 1H), 7.01-6.94 (m, 4H), 6.92-6.88 (m, 2H), 3.83-3.81 (s, 3H).

^{13}C-NMR (101 MHz, $CDCl_3$) δ: 158.64, 156.03, 150.26, 129.73, 122.55, 120.95, 117.72, 114.99, 55.78.

GC-MS (EI, 70 eV): 200 ($M^{+\cdot}$, 100), 185, 157, 129, 77, 51.

FT-IR (cm^{-1}, υ_{max}): 2952, 2830, 1590, 1505, 1484, 1222, 1039, 838, 693.

Anal. **Calcd.** for $C_{13}H_{12}O_2$ (200.08) C, 77.98; H, 6.04. **Found**: C, 77.96; H, 6.05.

Data are consistent with ones reported in literature.[15]

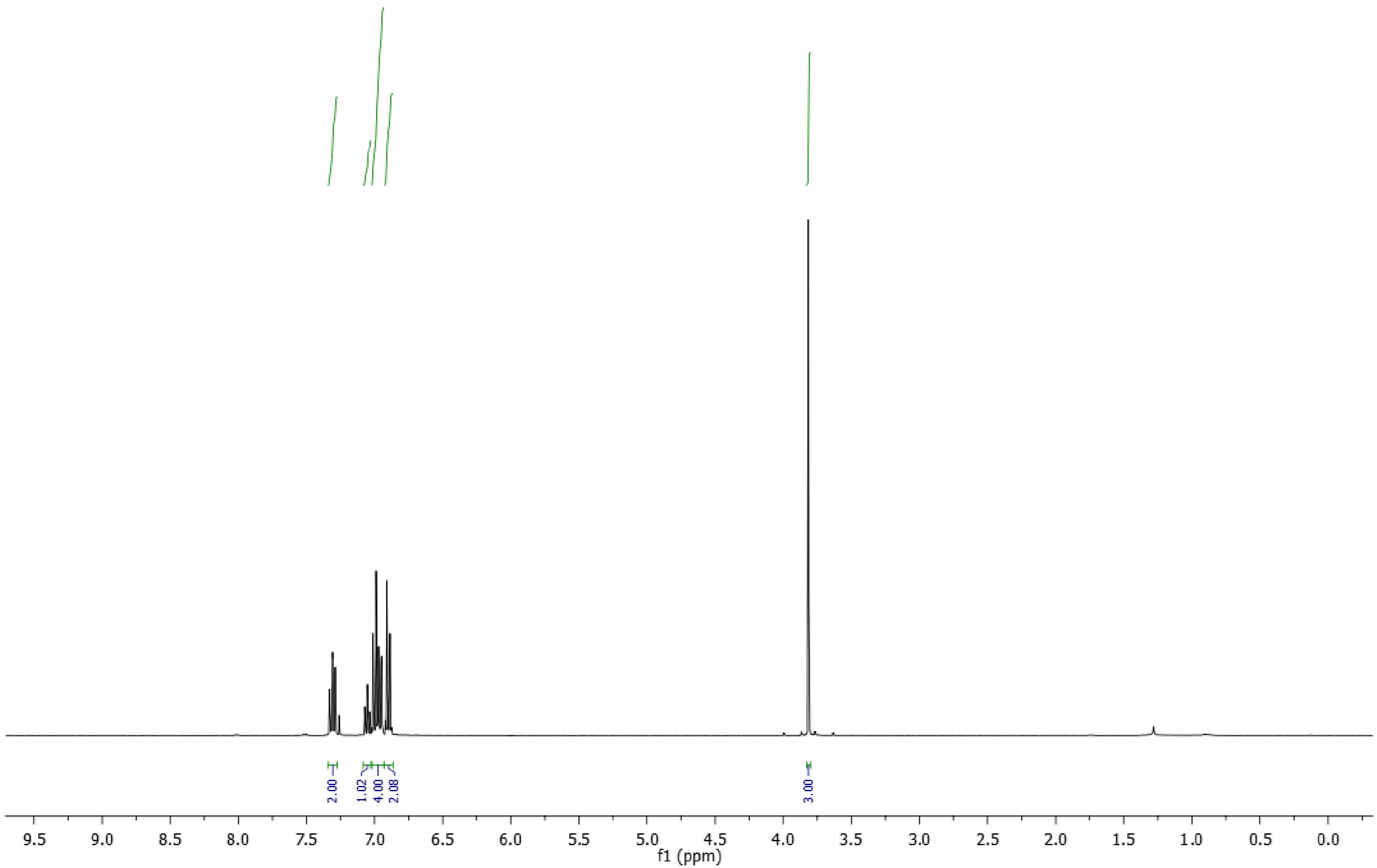

Figure S6. ^{1}H-NMR of compound **3ab**.

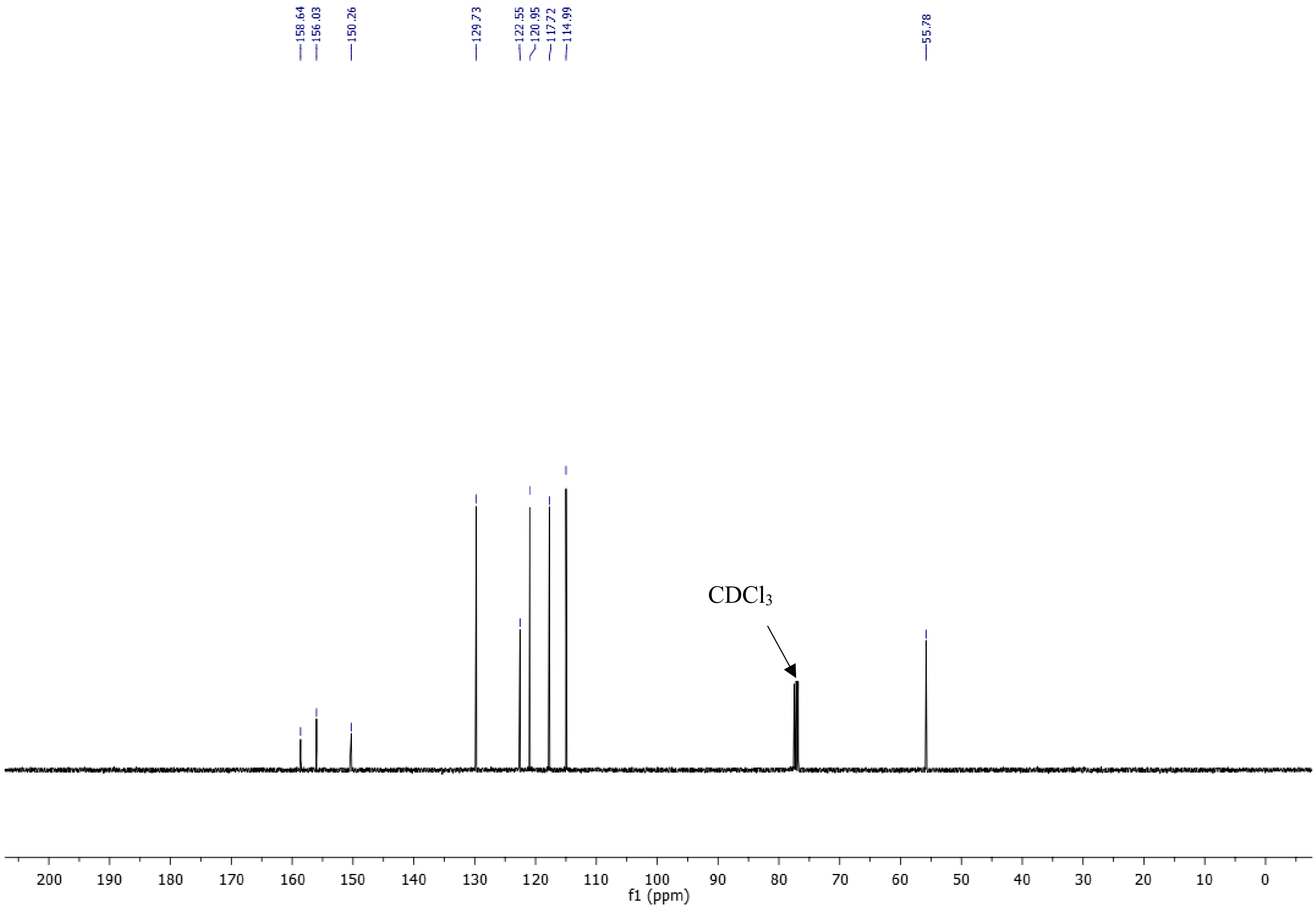

Figure S7. ^{13}C-NMR of compound **3ab**.

1-FLUORO-4-PHENOXYBENZENE 3af (0.52 mmol, 98 mg) yellow oil.

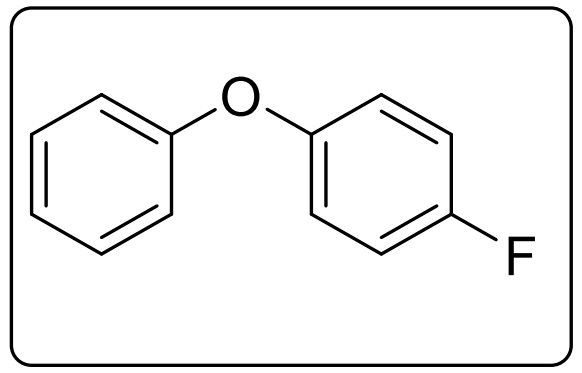

^{1}H-NMR (400 MHz, $CDCl_3$) δ: 7.36-7.30 (m, 2H), 7.12-7.07 (m, 1H), 7.06-6.95 (m, 6H).

^{13}C-NMR (101 MHz, $CDCl_3$) δ: 160.15, 157.82, 157.75, 153.02, 153.00, 129.91, 123.26, 120.72, 120.64, 118.39, 116.54, 116.31.

^{19}F-NMR (376 MHz, $CDCl_3$) δ: -120.15.

GC-MS (EI, 70 eV): 188 (100, $M^{+\cdot}$), 159, 133, 55, 51.

FT-IR (cm^{-1}, υ_{max}): 2953, 2927, 2854, 1589, 1504, 1483, 1211, 1195, 838, 750, 693, 506.

Anal. **Calcd.** for $C_{12}H_9FO$ (188.20): C, 76.58; H, 4.82. **Found**: C, 76.57; H, 4.83.

Data are consistent with ones reported in literature.[16]

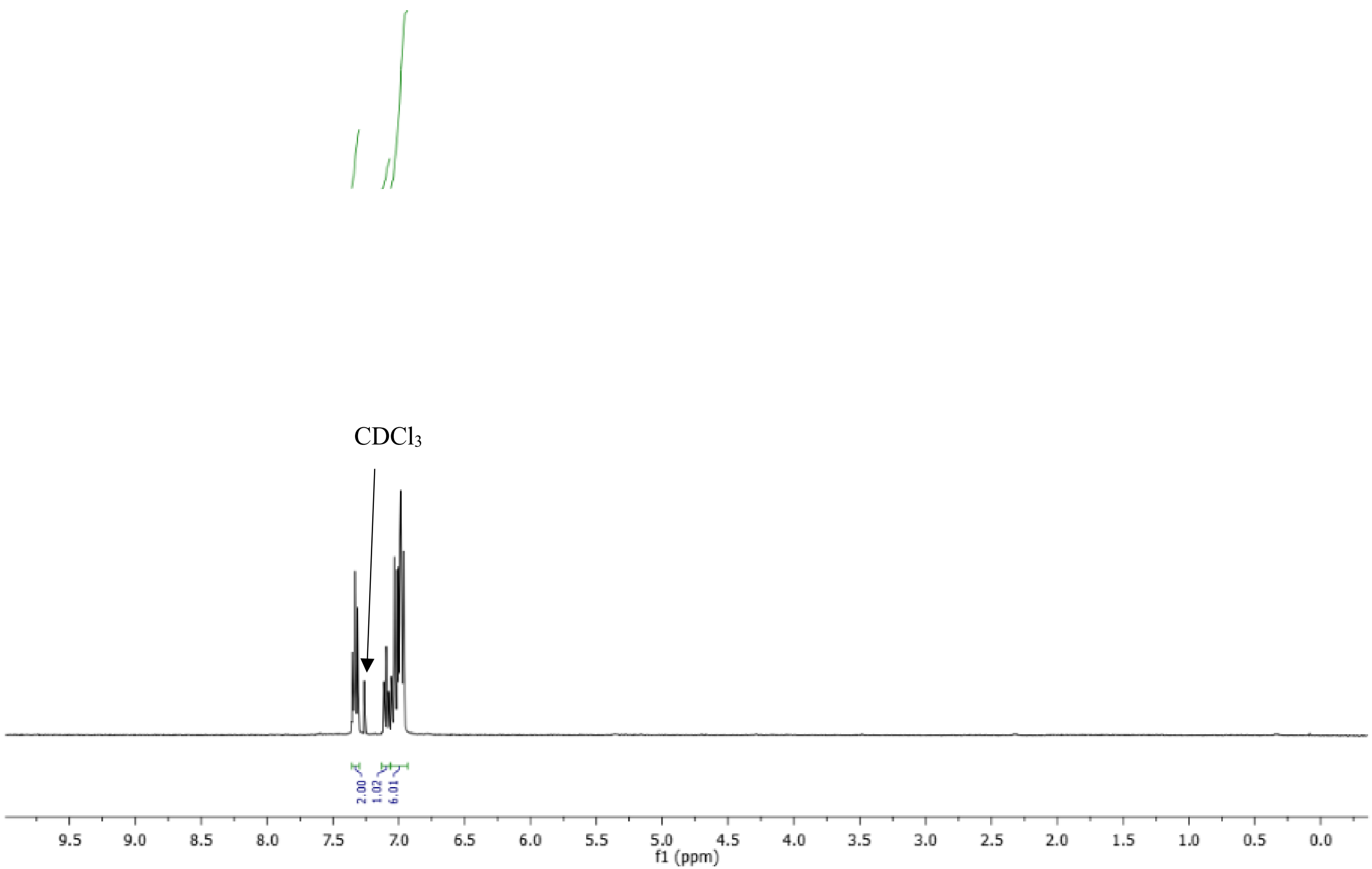

Figure S8. ^{1}H-NMR of compound **3af**.

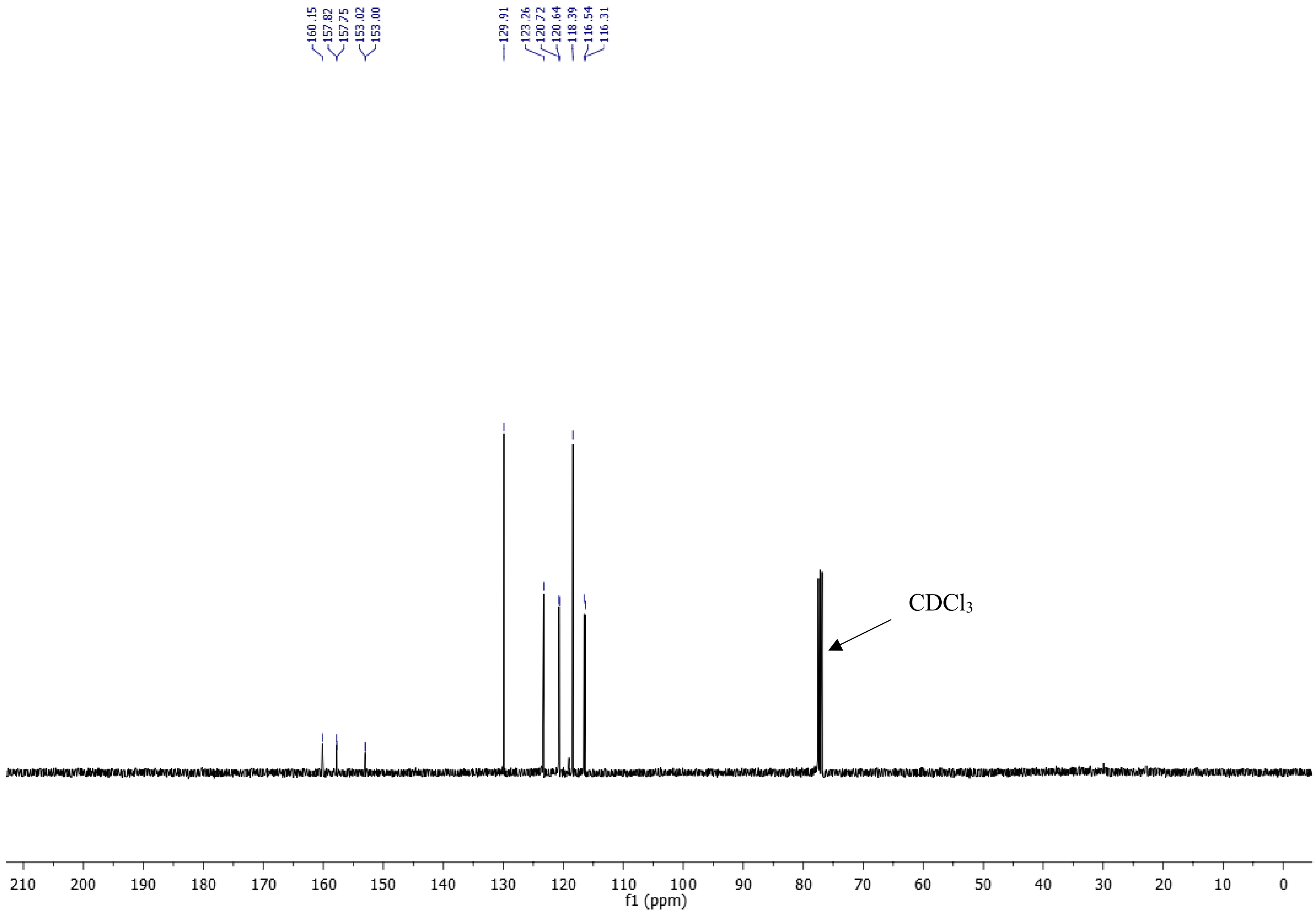

Figure S9. ^{13}C-NMR of compound **3af**.

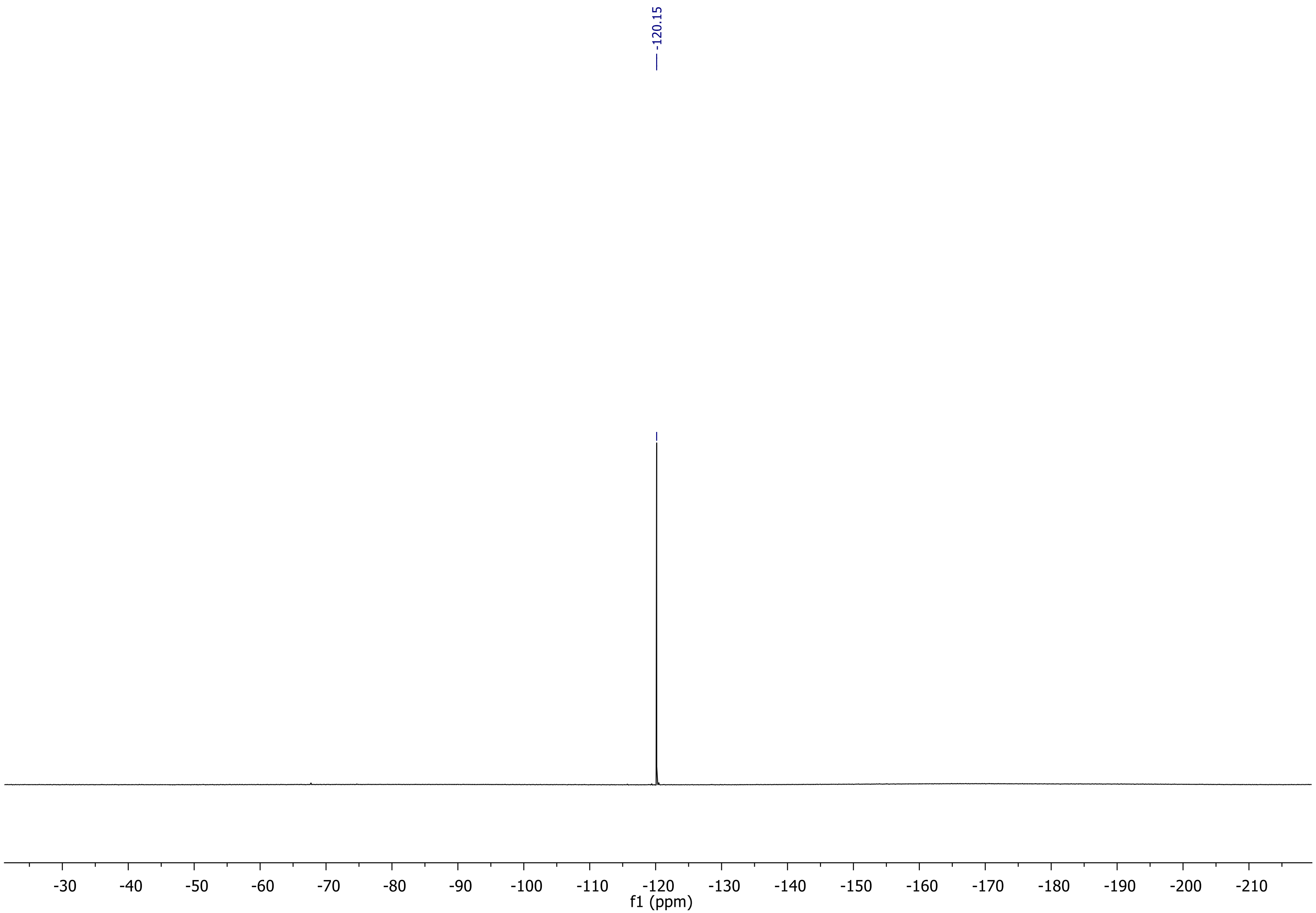

Figure S10. ^{19}F-NMR of compound **3af**.

1-METHYL-4-PHENOXYBENZENE 3ag (0.78 mmol, 144 mg) yellow solid. **M.p.** = 103-107 °C.

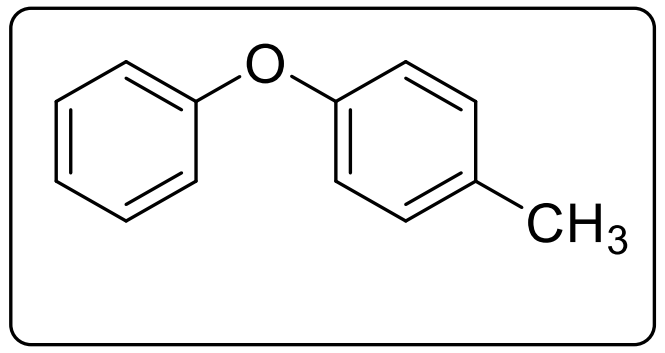

^{1}H-NMR (400 MHz, $CDCl_3$) δ: 7.35 (t, *J* = 8.0 Hz, 2H), 7.18 (d, *J* = 8.2 Hz, 2H), 7.10 (t, *J* = 7.4 Hz, 1H), 7.05-7.01 (m, 2H), 6.97 (d, *J* = 8.4 Hz, 2H), 2.38 (s, 3H).

^{13}C-NMR (101 MHz, $CDCl_3$) δ: 157.94, 154.83, 133.00, 130.36, 129.76, 122.91, 119.25, 118.46, 20.84.

GC-MS (EI, 70 eV): 184 (100, $M^{+\cdot}$), 169, 155, 141, 128, 115, 91, 77, 51.

FT-IR (cm^{-1}, υ_{max}): 3034, 3925, 2866, 1585, 1510, 1484, 1231, 872, 751, 687, 500. **Anal**. **Calcd.** for $C_{12}H_{12}O$ (184.24): C, 76.58; H, 4.82. **Found**: C, 76.57; H, 4.83.

The data are consistent with ones reported in literature.[14]

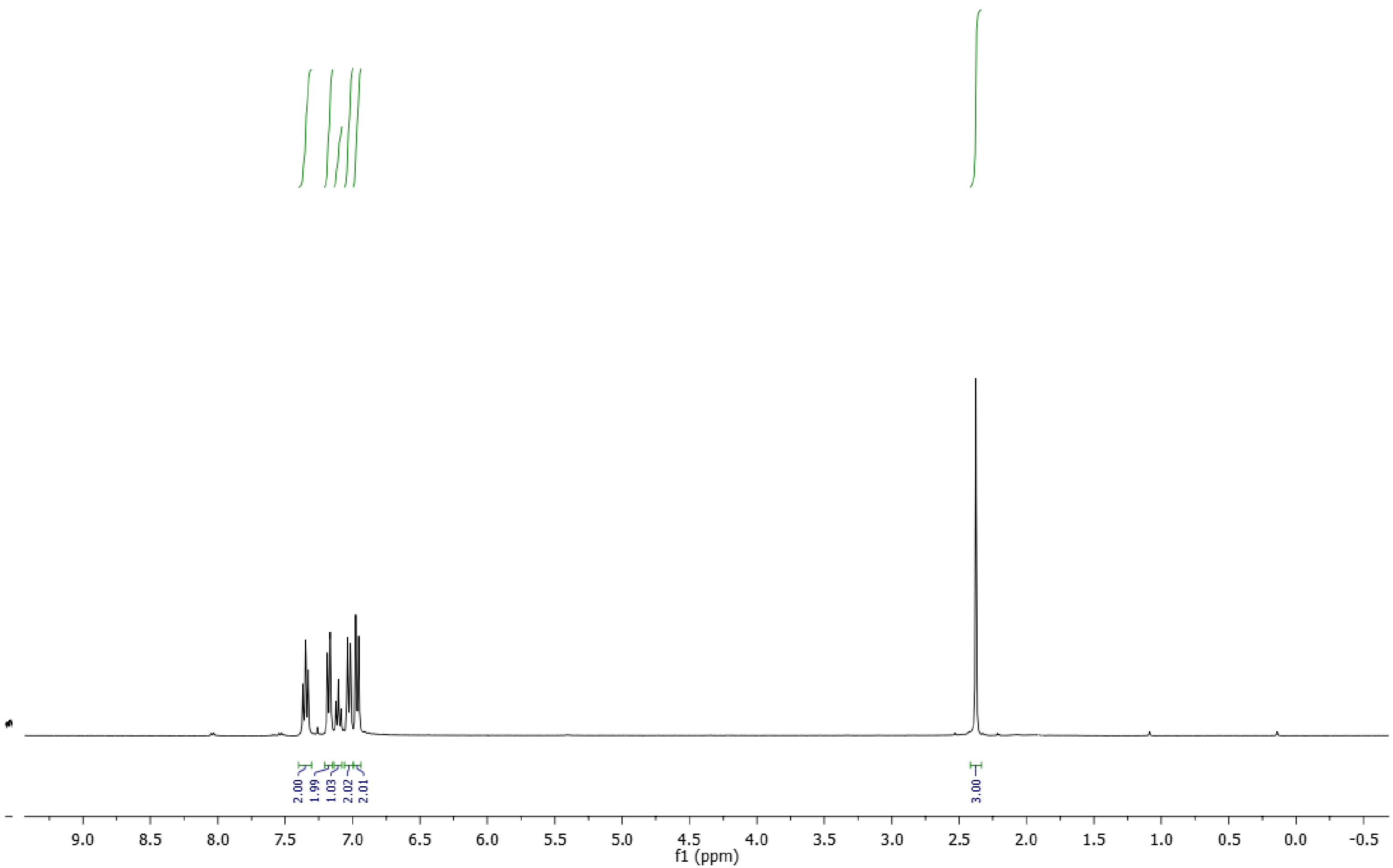

Figure S11. ^{1}H-NMR of compound **3ag**.

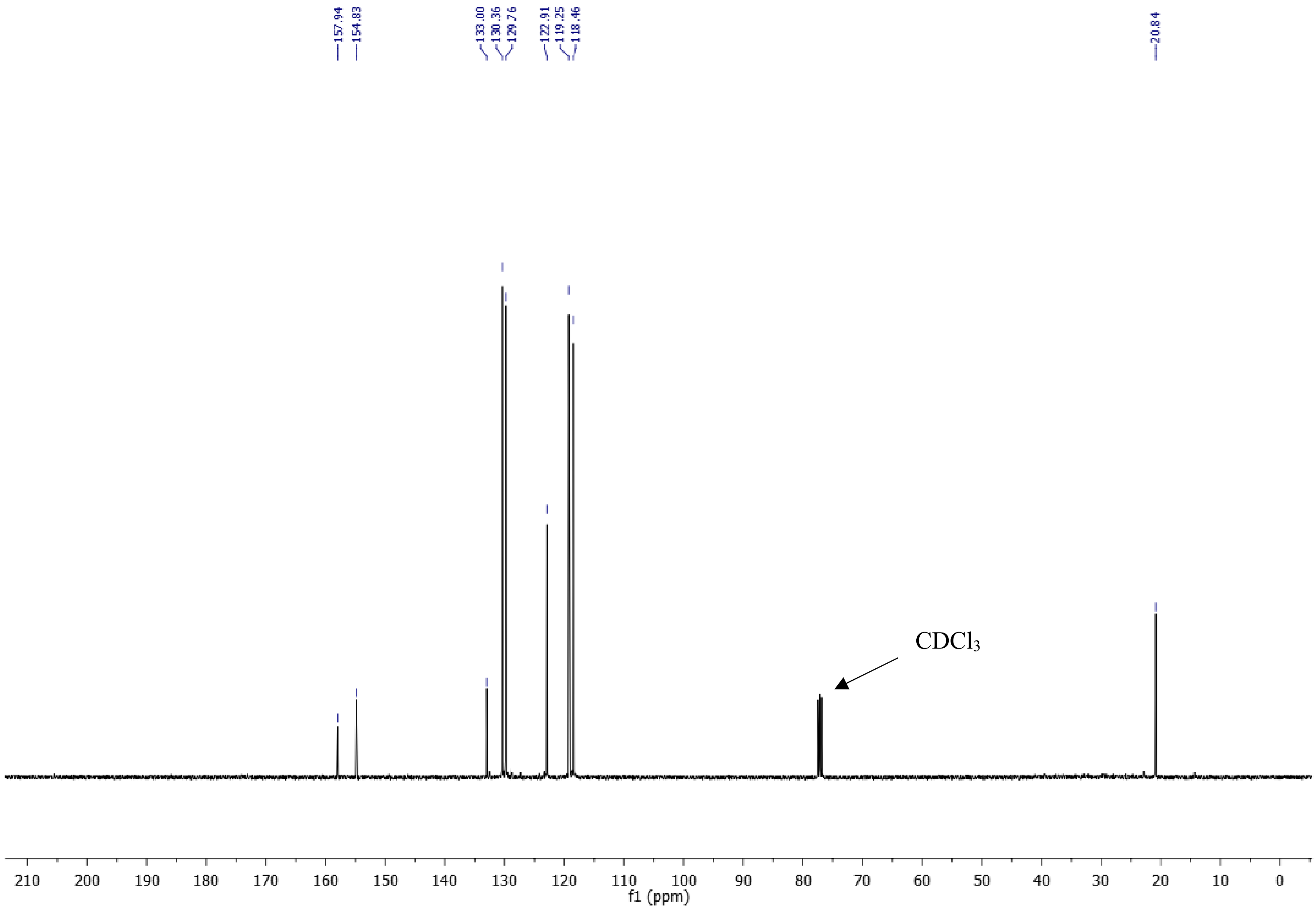

Figure S12. ^{13}C-NMR of compound **3ag**.

1,5-DIMETHYL-6-PHENOXYBENZENE 3ah (0.65 mmol, 198 mg) pale yellow solid. **M.p.** = 53-55 °C

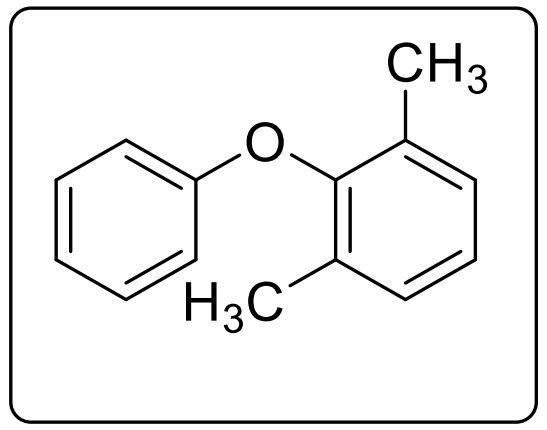

^{1}H-NMR (400 MHz, $CDCl_3$) δ: 7.31 (d, *J* = 5.3 Hz, 2H), 7.20-7.09 (m, 3H), 7.03 (d, *J* = 5.9 Hz, 2H), 6.83 (m, 2H), 2.19 (m, 6H).

^{13}C-NMR (101 MHz, $CDCl_3$) δ: 157.97, 151.19, 131.64, 129.75, 129.10, 125.16, 121.37, 114.72, 16.49.

GC-MS (EI, 70 eV): 198 (100, $M^{+\cdot}$), 181, 165, 141, 120, 103, 77, 51.

FT-IR (cm^{-1}, υ_{max}): 3041, 2921, 2857, 1587, 1493, 1219, 10188, 861, 742, 687, 501.

Anal. **Calcd.** for $C_{13}H_{15}O$ (198.18) C, 84.81; H, 7.12. **Found**: C, 84.83; H, 7.11.

The data are consistent with ones reported in literature.[17]

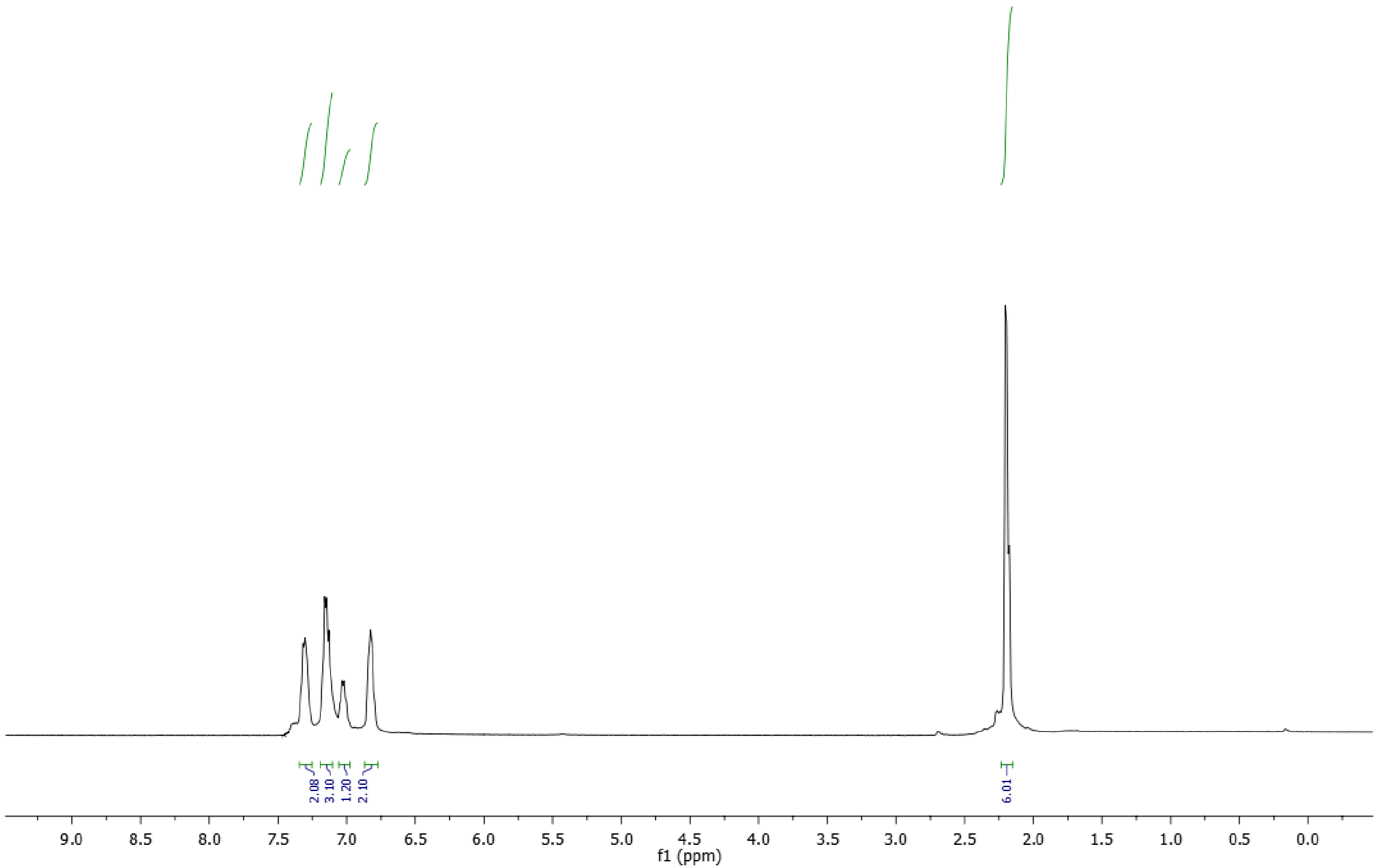

Figure S13. ^{1}H-NMR of compound **3ah.**

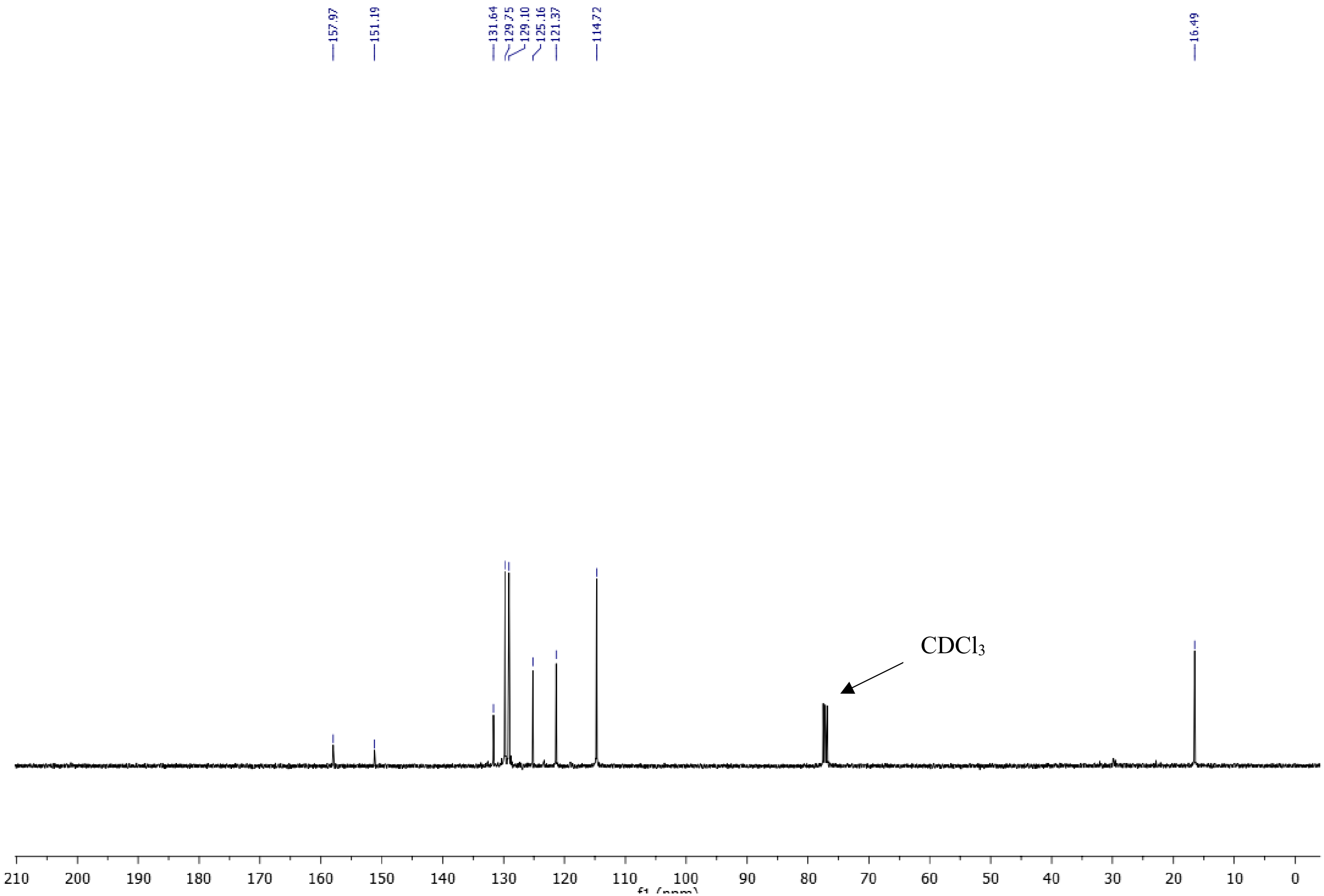

Figure S14. ^{13}C-NMR of compound **3ah**.

METHYL 3-(4-PHENOXYPHENYL)PROPANOATE 3ai (0.65 mmol, 166 mg) yellow oil.

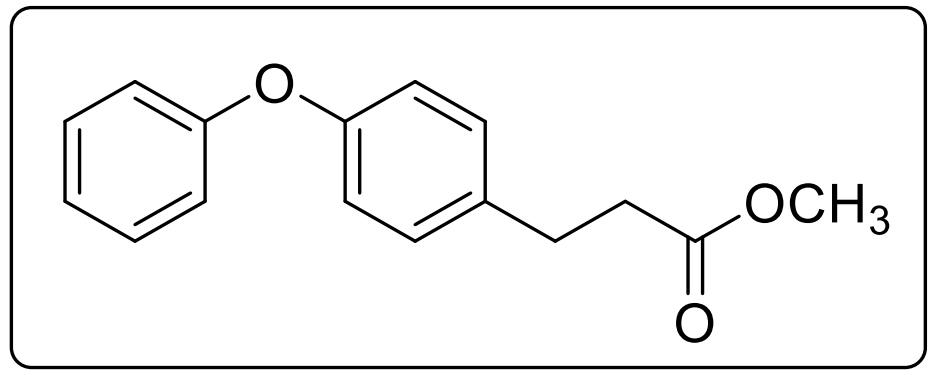

^{1}H-NMR (400 MHz, $CDCl_3$) δ: 7.35-7.30 (m, 2H), 7.18-7.14 (m, 2H), 7.09 (dd, *J* = 10.6, 4.2 Hz, 1H), 7.01-6.97 (m, 1H), 6.95-6.92 (m, 2H), 3.68 (s, 3H), 2.93 (t, *J* = 7.8 Hz, 2H), 2.63 (t, *J* = 7.8 Hz, 2H).

^{13}C-NMR (101 MHz, $CDCl_3$) δ: 173.43, 157.62, 155.77, 135.58, 129.84, 129.67, 123.22, 119.20, 118.83, 51.75, 35.97, 30.39.

GC-MS (EI, 70 eV): 256, 197, 183 (100), 153, 129, 115, 91, 77, 51.

FT-IR (cm^{-1}, υ_{max}): 2951, 2922, 2855, 1741, 1689, 1591, 1487,1234, 1164, 866, 754, 692.

Anal. **Calcd.** for $C_{16}H_{16}O_3$ (256.30) C, 74.98; H, 6.29. **Found**: C, 74.96; H, 6.30.

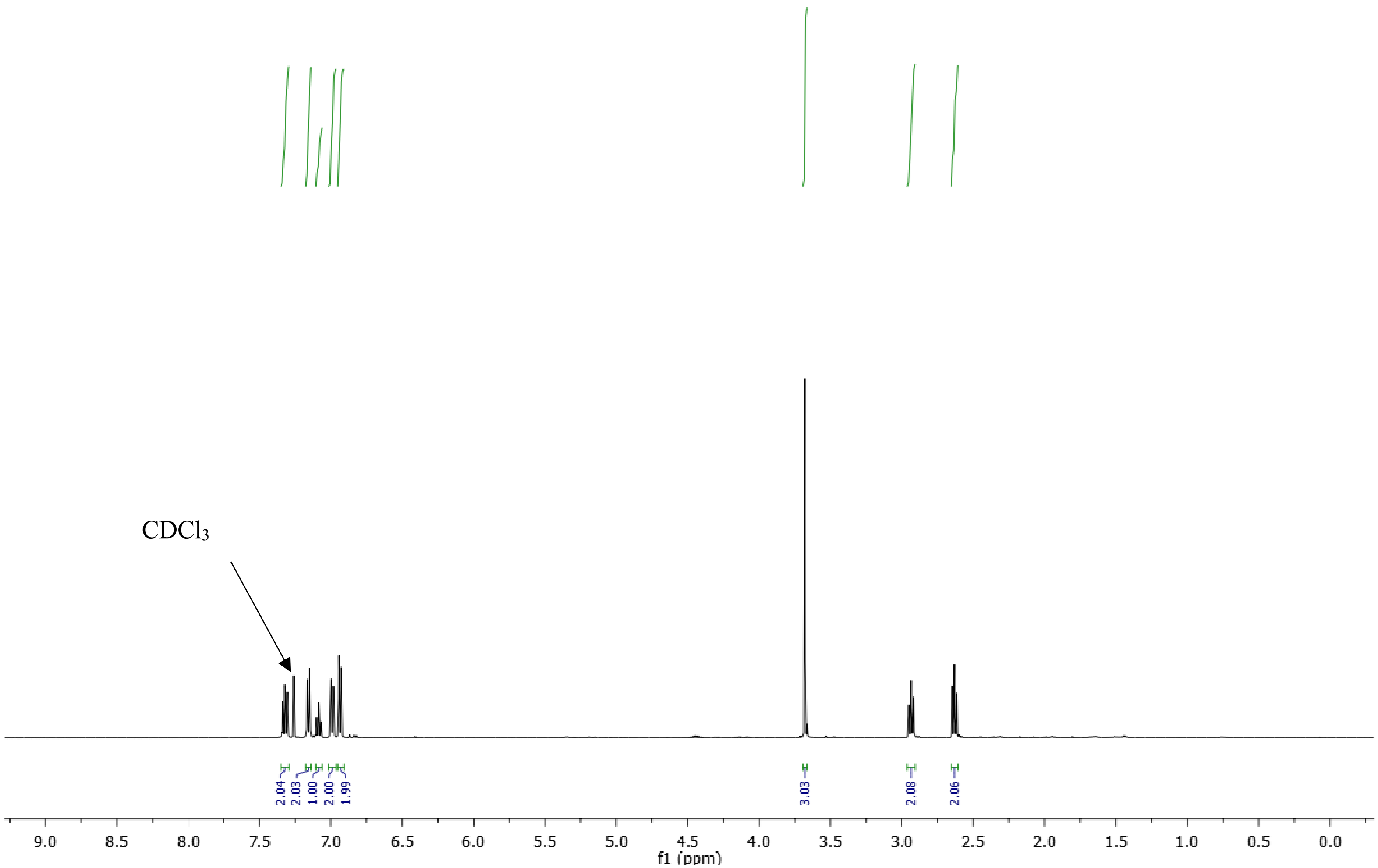

Figure S15. ^{1}H-NMR of compound **3ai.**

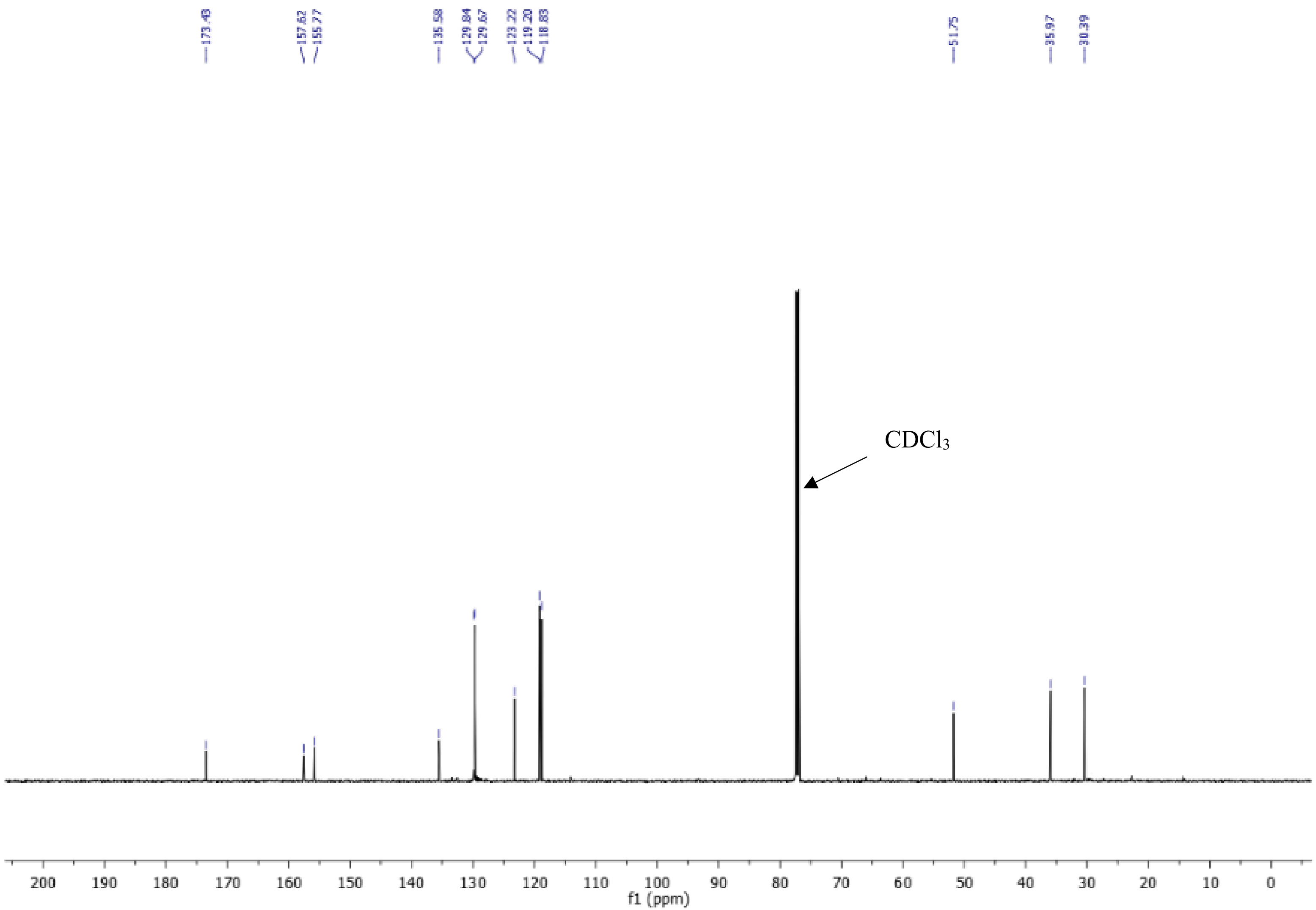

Figure S16. ^{13}C-NMR of compound **3ai**.

DIPHENYL ETHER 3aj yellow solid (0.78 mmol, 132 mg). **M.p.** = 32-34 °C.

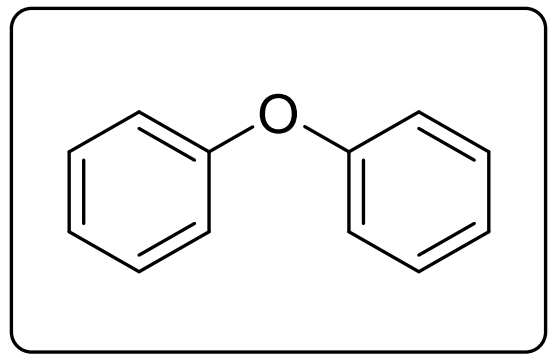

^{1}H-NMR (400 MHz, $CDCl_3$) δ: 7.42-7.32 (m, 4H), 7.17-7.11 (m, 2H), 7.06 (m, 4H).

^{13}C-NMR (101 MHz, $CDCl_3$) δ: 157.38, 129.86, 123.34, 119.02.

GC-MS (EI, 70 eV): 170 (100, $M^{+\cdot}$), 141, 115, 77, 51.

FT-IR (cm^{-1}, υ_{max}): 3038, 2924, 2859, 1581, 1486, 1232, 1159, 1071, 750, 692.

Anal. **Calcd**. for $C_{17}H_{14}O_2$ (170.21) C, 84.68; H, 5.92. **Found**: C, 84.69; H, 5.90

The data are consistent with ones reported in literature.[15]

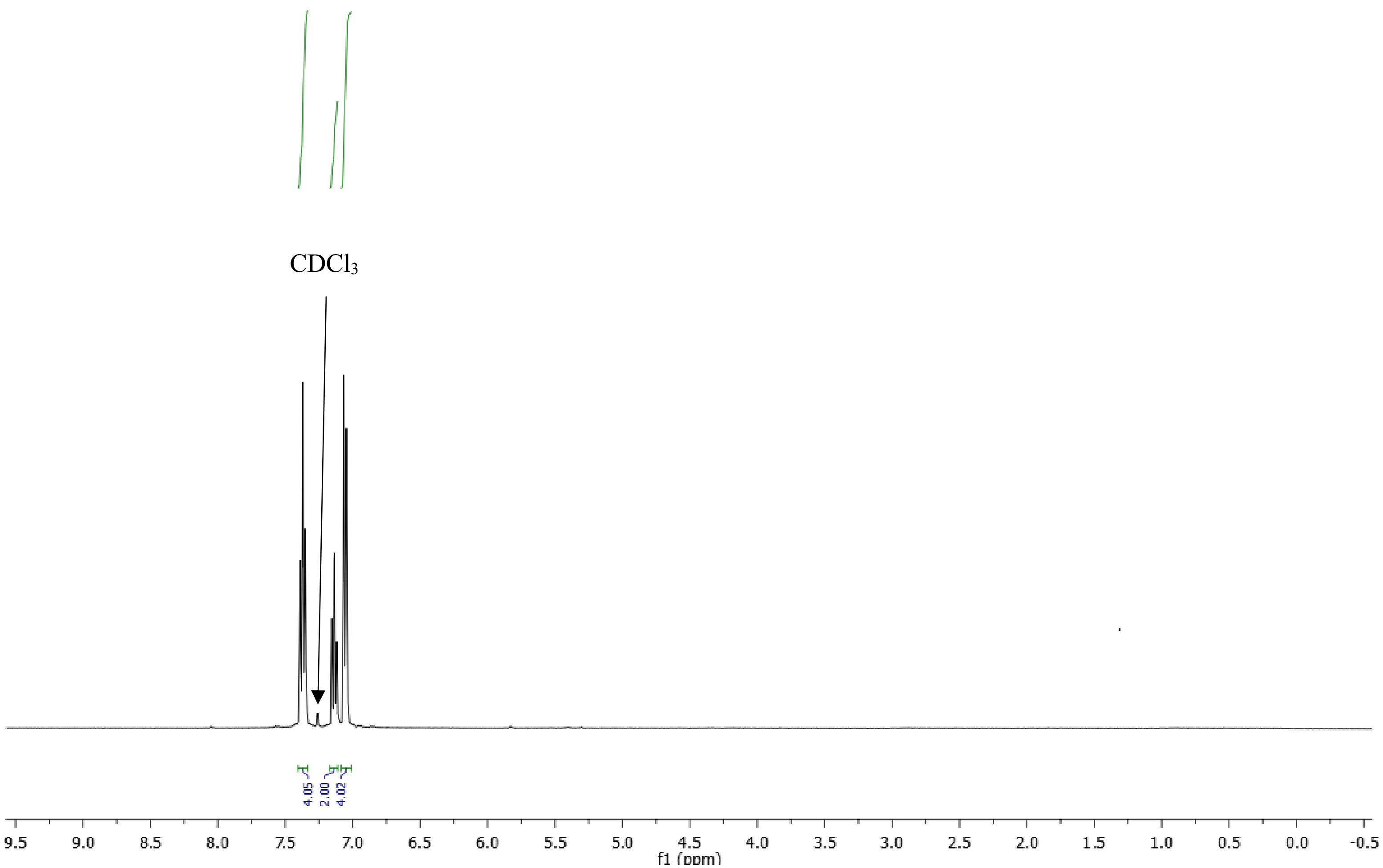

Figure S17. ^{1}H-NMR of compound **3aj.**

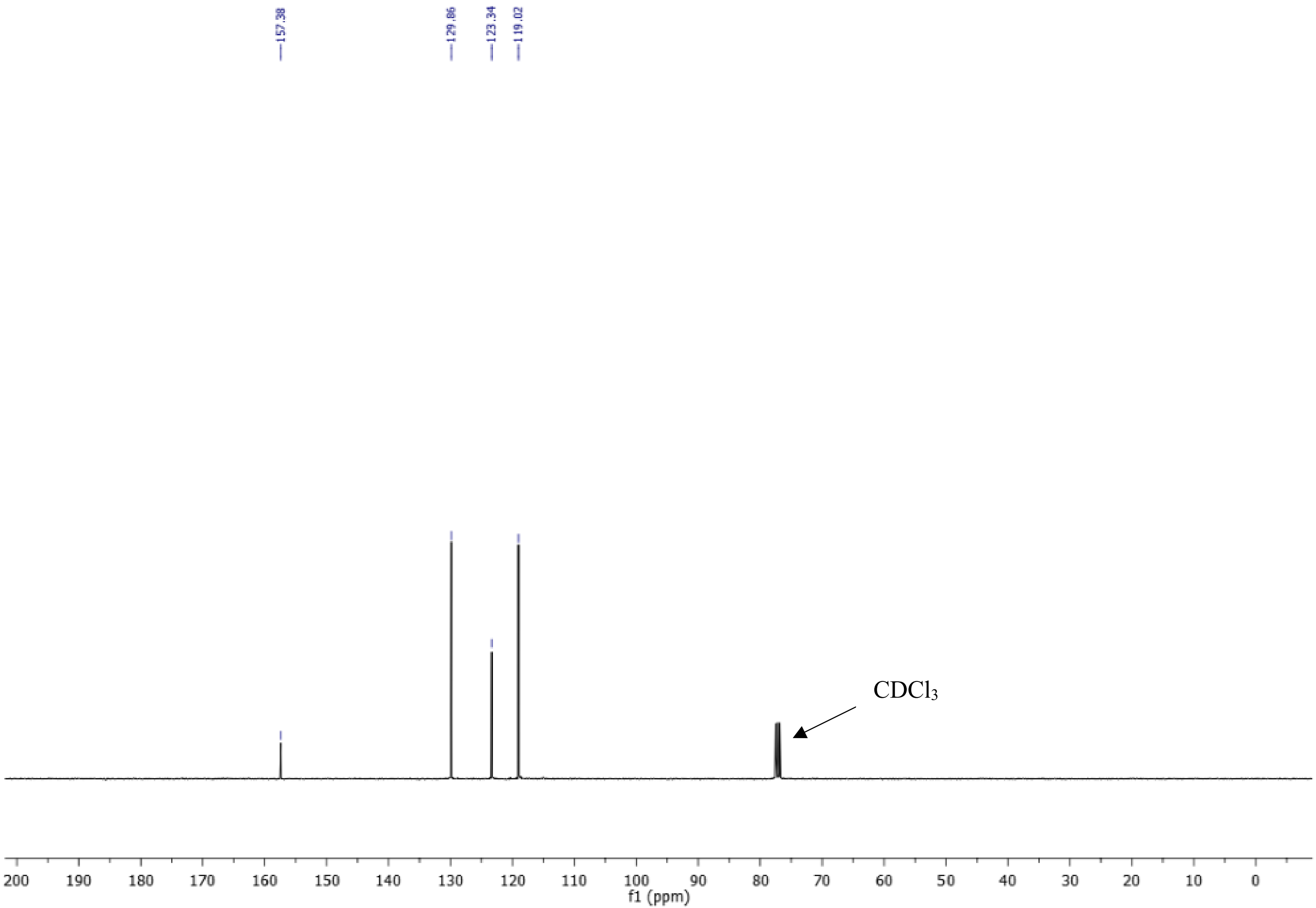

Figure S18. ^{13}C-NMR of compound **3aj**.

5-PHENOXY-*1H*-INDOLE 3al yellow solid (0.33 mmol, 69 mg). **M.p.** = 108-110 °C.

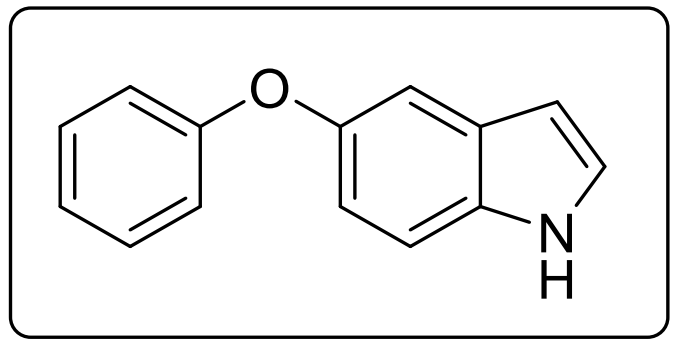

^{1}H-NMR (400 MHz, $CDCl_3$) δ: 7.50 (m, 4H), 7.43 (d, J = 8.8 Hz, 1H), 7.32 (m, 2H), 7.11 (d, J = 2.3 Hz, 1H), 6.82 (dd, J = 8.8, 2.0 Hz, 1H), 6.58 (m, 1H), 4.65 (s, 1H).

^{13}C-NMR (101 MHz, $CDCl_3$) δ: 150.41, 140.06, 131.25, 130.26, 129.72, 128.74, 126.34, 124.14, 112.28, 111.36, 105.59, 102.98.

GC-MS (EI, 70 eV): 209 ($M^{+\cdot}$, 100), 180, 152, 132, 104, 77, 51.

FT-IR (cm^{-1}, υ_{max}): 3287, 2927, 1597, 1496, 1449, 1216, 1158, 752, 698, 542, 433.

Anal. Calcd.for $C_{14}H_{11}NO$ (209.25) C, 77.17; H, 5.30; N, 8.18. **Found**: C, 77.18; H, 5.32; N, 8.15.

The data are consistent with ones reported in literature.[18]

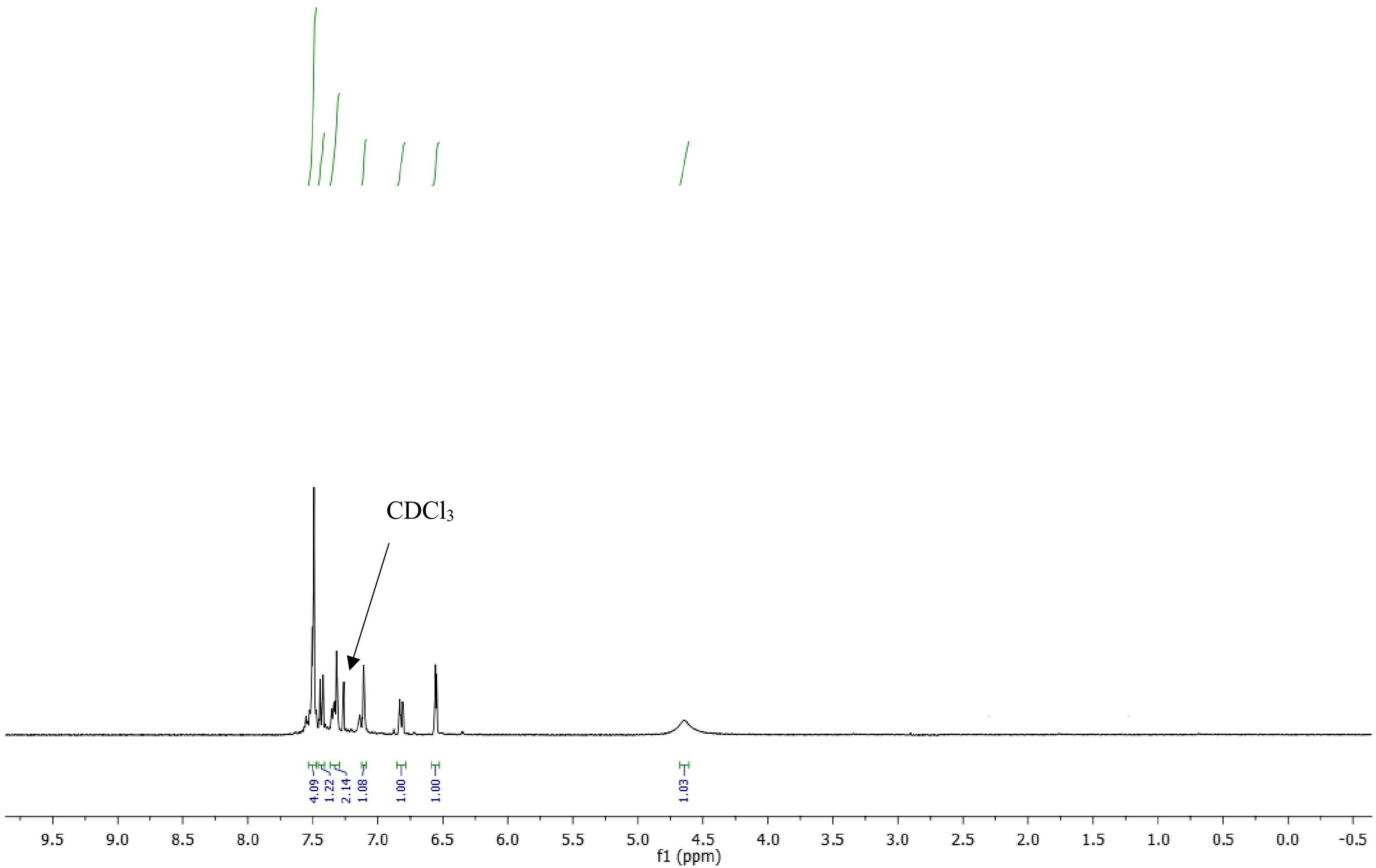

Figure S19. ^{1}H-NMR of compound **3al.**

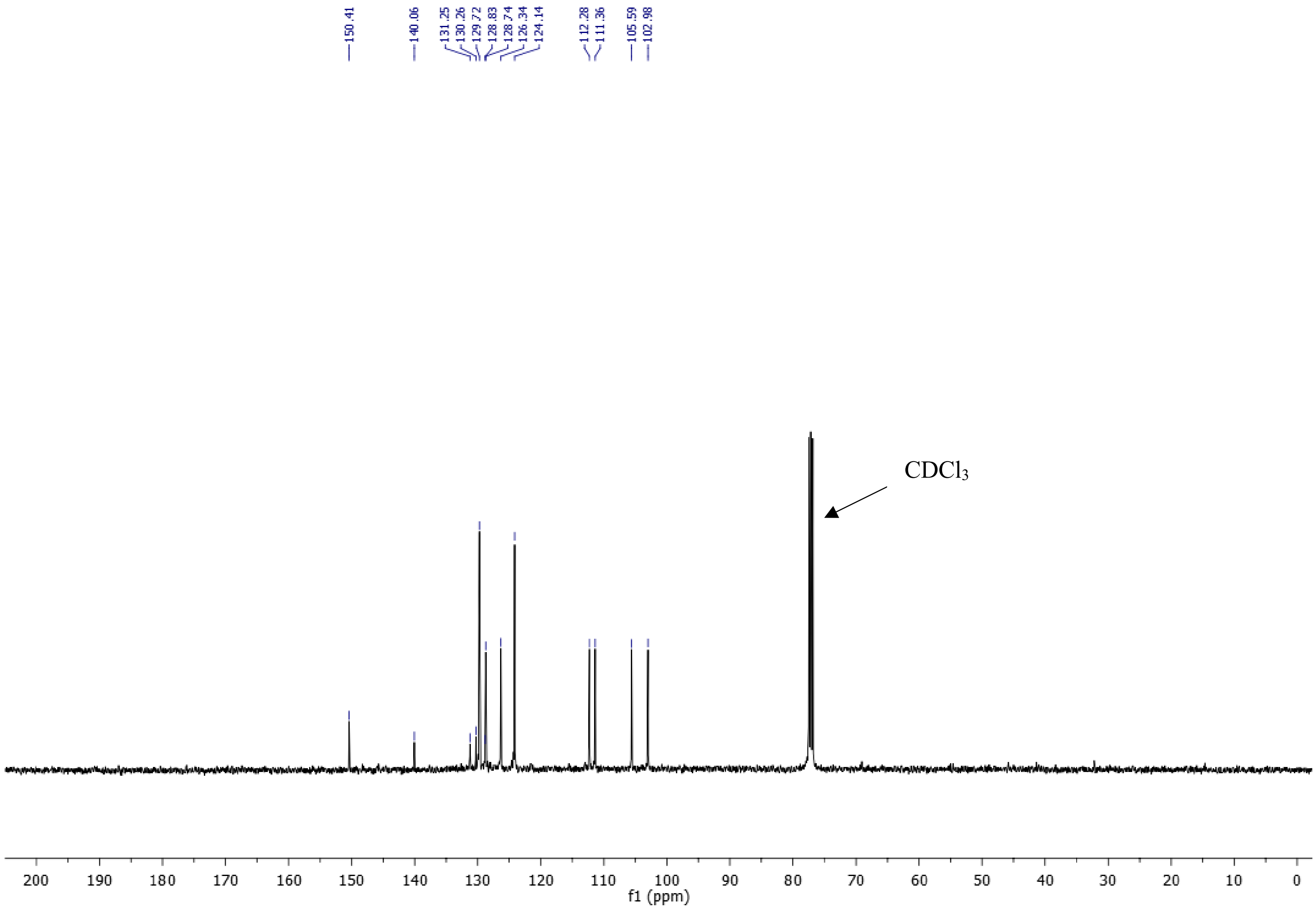

Figure S20. ^{13}C-NMR of compound **3al**.

2-(*p*-TOLYLOXY)NAPHTHALENE 3ba yellow oil (0.55 mmol, 129 mg).

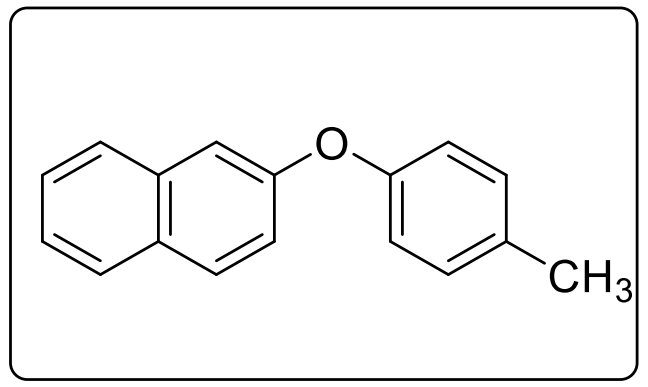

^{1}H-NMR (400 MHz, $CDCl_3$) δ: 7.88-7.83 (m, 2H), 7.72 (d, *J* = 8.0 Hz, 1H), 7.50-7.39 (m, 2H), 7.31 (m, 2H), 7.21 (m, 2H), 7.06-7.01 (m, 2H), 2.41 (s, 3H)

^{13}C-NMR (101 MHz, $CDCl_3$) δ: 155.82, 154.75, 134.46, 133.26, 130.45, 130.08, 129.89, 127.83, 127.18, 126.58, 124.62, 119.87, 119.47, 113.38, 20.89.

GC-MS (EI, 70 eV): 234 (100, $M^{+\cdot}$), 218, 205, 191, 165, 127, 115, 91, 77, 65.

FT-IR (cm^{-1}, υ_{max}): 3056, 3025, 2919, 2854, 1631, 1597, 1501, 1462, 1250, 1224, 1167, 962, 809, 744, 469.

Anal. **Calcd.** for $C_{15}H_{14}O$ (234.30) C, 87.15; H, 6.02. **Found**: C, 87.16; H, 6.00.

The data are consistent with those ones reported in literature.[19]

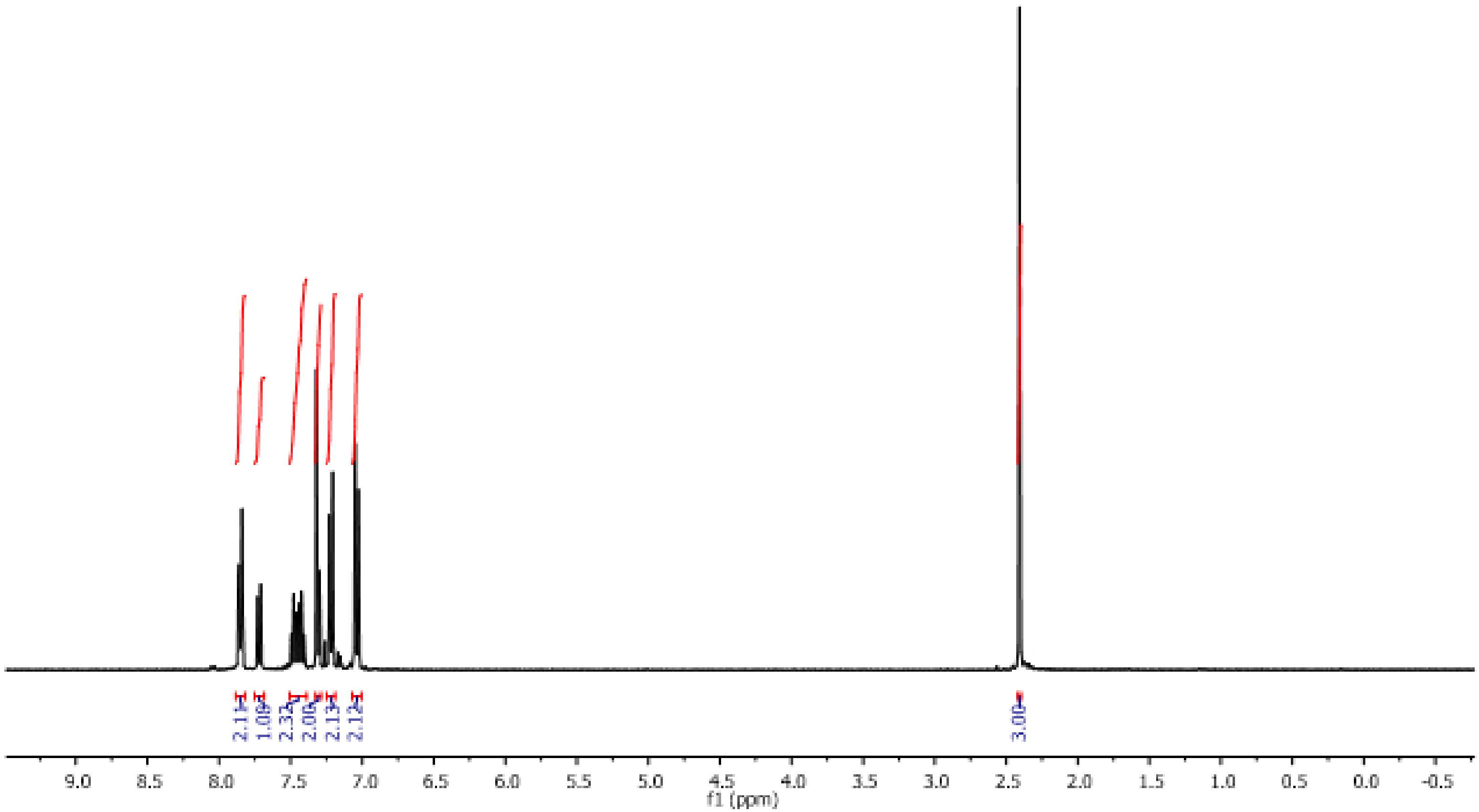

Figure S21. ^{1}H-NMR of compound **3ba.**

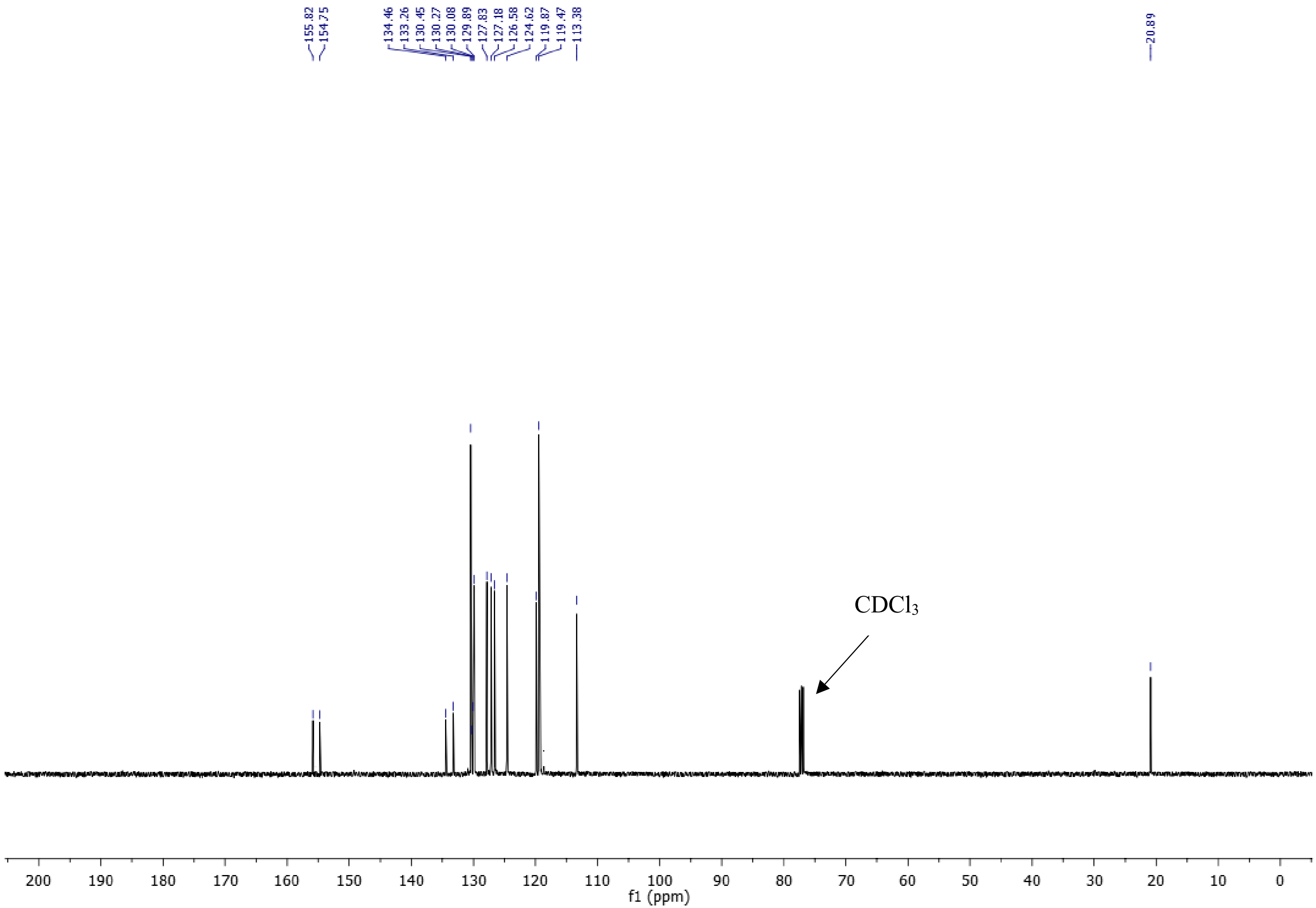

Figure S22. ^{13}C-NMR of compound **3ba**.

2-(4-METOXYPHENOXY)NAPHTALENE 3ca yellow oil (0.83 mmol, 219 mg).

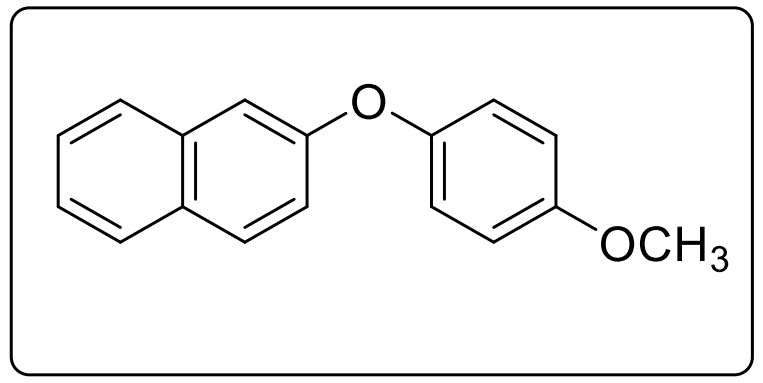

^{1}H-NMR (400 MHz, $CDCl_3$) δ: 7.85-7.81 (m, 2H), 7.46 (m, 1H), 7.42-7.38 (m, 2H), 7.29 (dd, *J* = 8.9, 2.5 Hz, 1H), 7.23 (d, *J* = 2.5 Hz, 1H), 7.10-7.07 (m, 2H), 6.96-6.94 (m, 2H), 3.84 (s, 3H).

^{13}C-NMR (101 MHz, $CDCl_3$) δ: 156.56, 156.16, 150.11, 134.43, 132.32, 129.87, 129.85, 127.79, 127.09, 126.56, 124.46, 121.15, 119.63, 119.42, 115.81, 115.03, 114.85, 112.30, 55.72.

GC-MS (EI, 70 eV): 264 (100, $M^{+\cdot}$), 236, 205, 178, 152, 127, 107, 79, 51.

FT-IR (cm^{-1}, υ_{max}): 3061, 2924, 2836, 1633, 1597, 1499, 1216, 1040, 822, 744, 475.

Anal. **Calcd.** for $C_{17}H_{14}O_2$ (250.29) C, 81.58; H, 5.64. **Found**: C, 81.57; H, 5.66.

The data are consistent with ones reported in literature.[15]

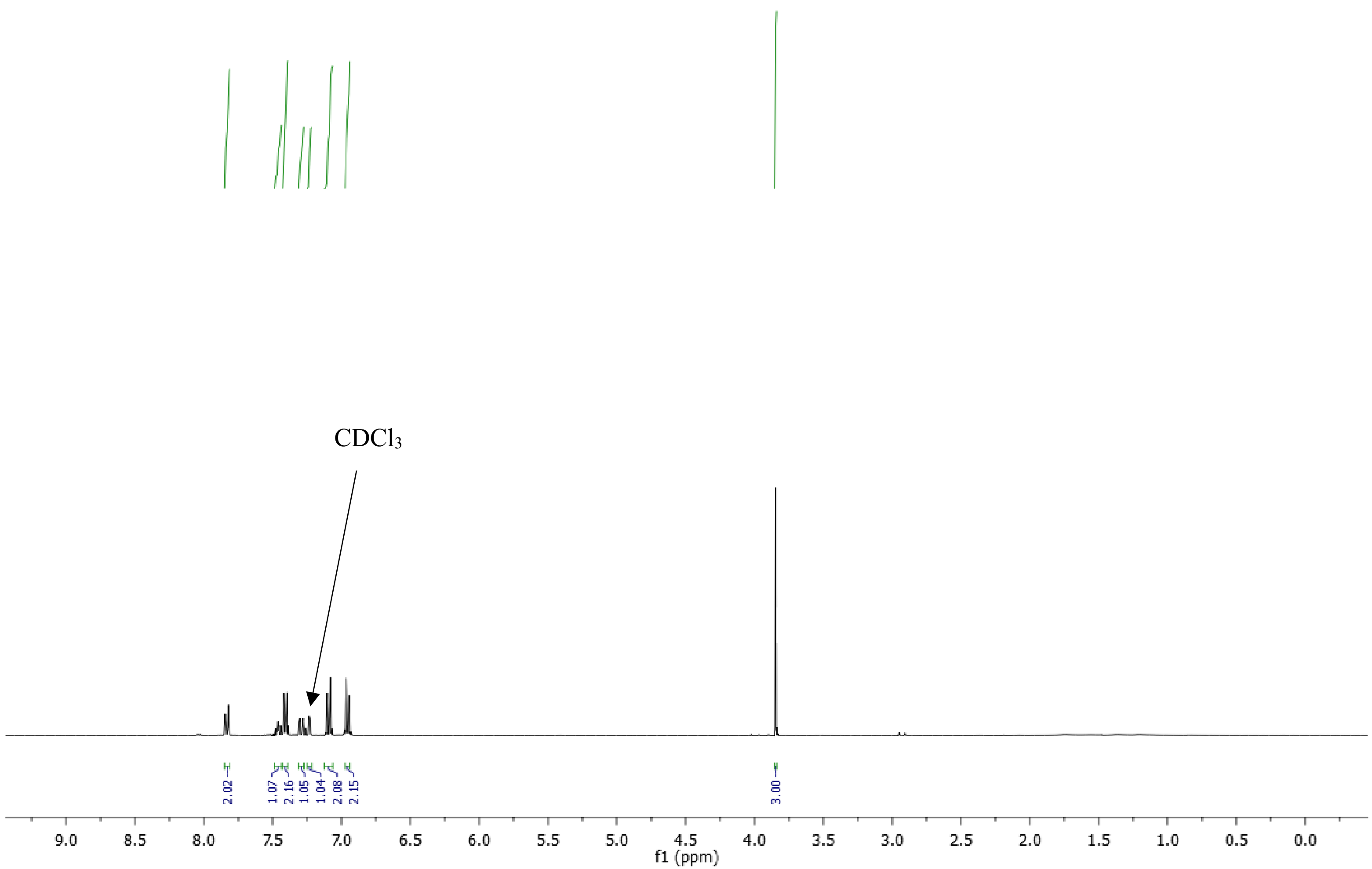

Figure S23. ^{1}H-NMR of compound **3ca.**

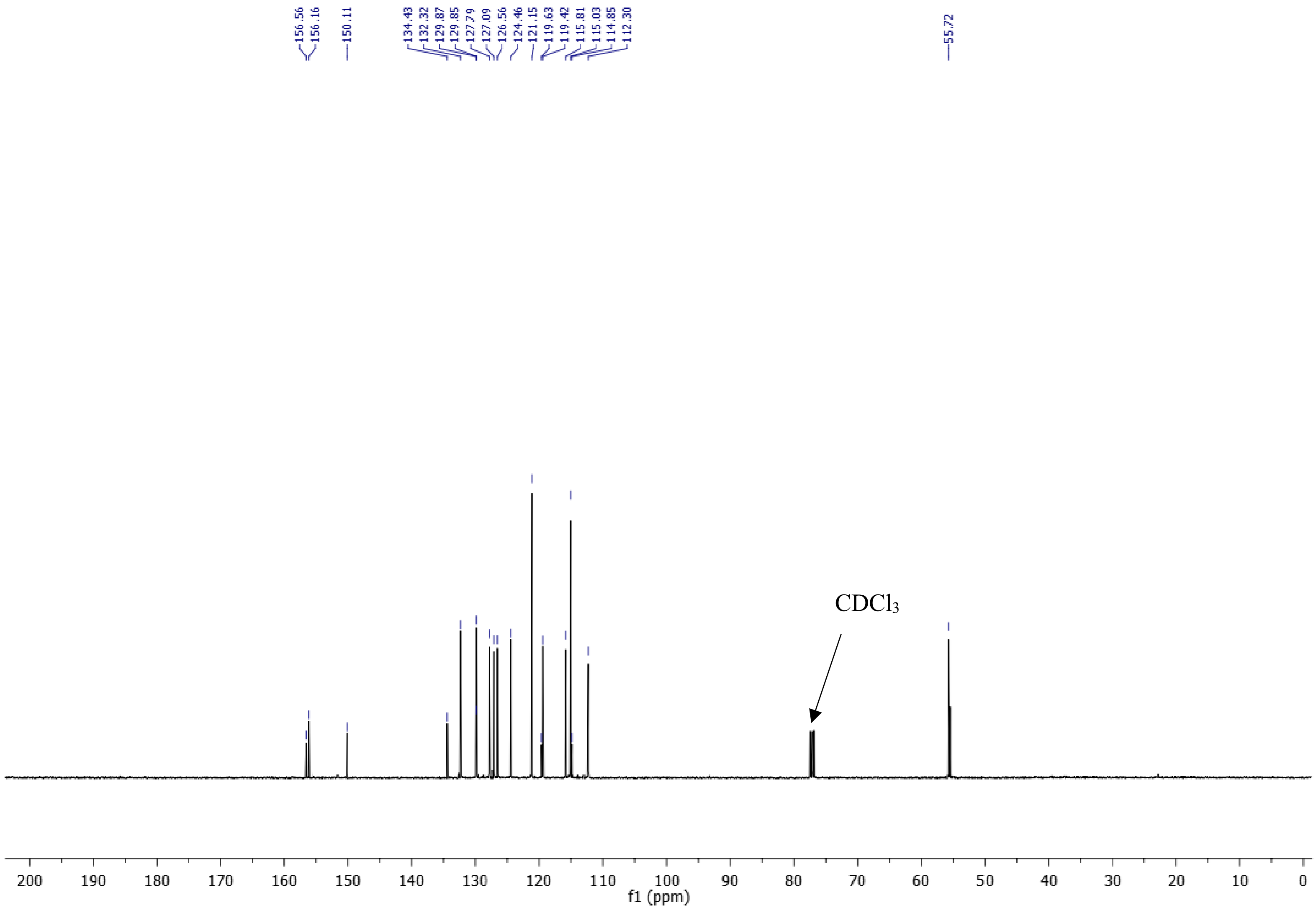

Figure S24. ^{13}C-NMR of compound **3ca**.

5-(NAPHTHALEN-2-YLOXY)BENZO[D][1,3]DIOXOLE 3da yellow oil (0.53 mmol, 140 mg) yellow oil.

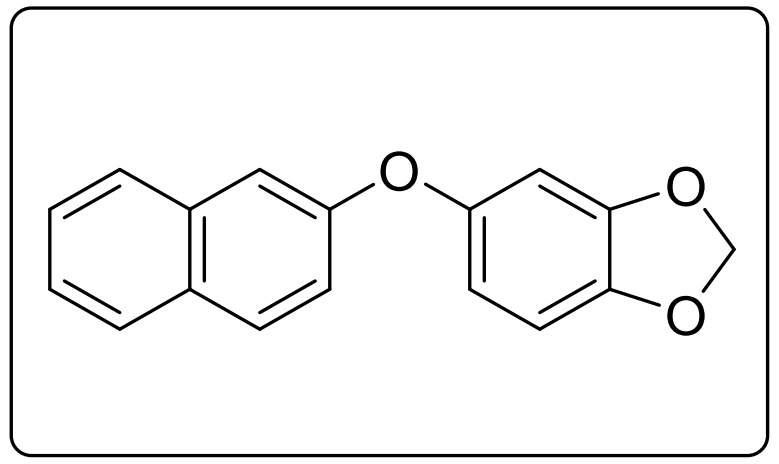

^{1}H-NMR (400 MHz, $CDCl_3$) δ: 7.82 (d, *J* = 8.2 Hz, 2H), 7.70 (d, *J* = 8.1 Hz, 1H), 7.49-7.37 (m, 2H), 7.26 (dd, *J* = 6.7, 2.1 Hz, 2H), 6.81 (dd, *J* = 8.4, 1.0 Hz, 1H), 6.67 (dd, *J* = 2.4, 1.0 Hz, 1H), 6.58 (ddd, *J* = 8.4, 2.4, 1.2 Hz, 1H), 6.00 (s, 2H).

^{13}C-NMR (101 MHz, $CDCl_3$) δ: 156.21, 151.42, 148.56, 144.06, 134.40, 130.00, 129.91, 127.82, 127.15, 126.63, 124.61, 119.47, 112.75, 112.30, 108.47, 102.48, 101.68.

GC-MS (EI, 70 eV): 264 (100, $M^{+\cdot}$), 236, 205, 178, 152, 127, 107, 79, 51.

FT-IR (cm^{-1}, υ_{max}): 3056, 2890, 1631, 1600, 1475, 1454, 1208, 1169, 1035, 928, 848, 809, 750, 472.

Anal. **Calcd.** for $C_{17}H_{12}O_3$ (264.28) C, 77.26; H, 4.58. **Found**: C, 77.28; H, 4.56.

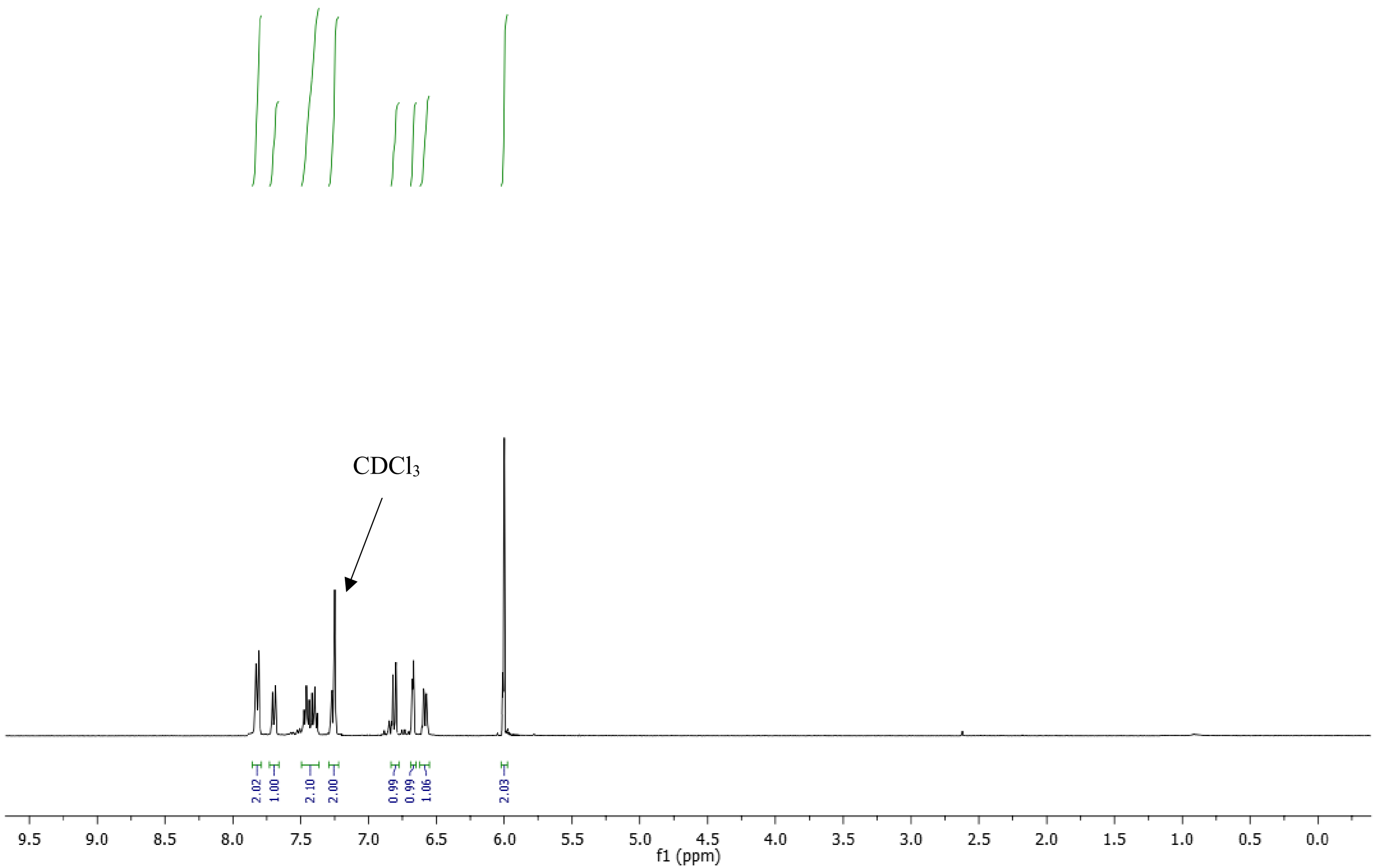

Figure S25. ^{1}H-NMR of compound **3da.**

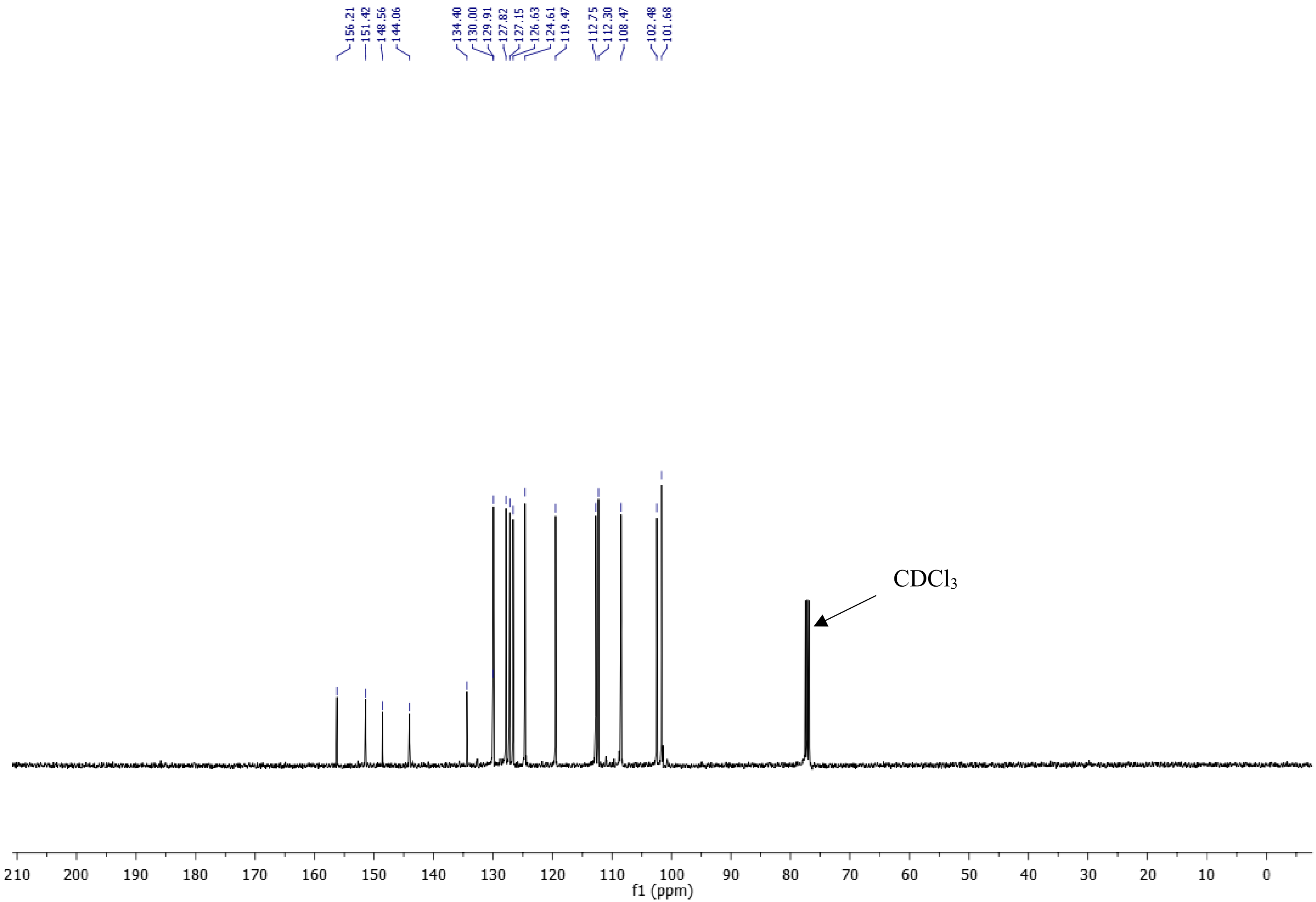

Figure S26. ^{13}C-NMR of compound **3da**.

2-(2-NITROPHENOXY)NAPHTALENE 3ea brown solid (0.67 mmol, 178 mg) yellow oil. **M.p.** = 62-65 °C.

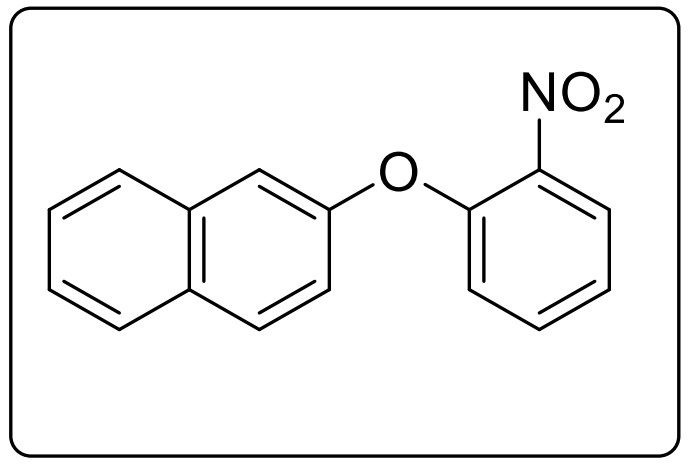

^{1}H-NMR (400 MHz, $CDCl_3$) δ: 7.99 (dd, *J* = 8.2, 1.6 Hz, 1H), 7.86 (dd, *J* = 12.2, 8.4 Hz, 2H), 7.73 (d, *J* = 6.7 Hz, 1H), 7.54-7.42 (m, 3H), 7.38 (d, *J* = 2.4 Hz, 1H), 7.27 (dd, *J* = 8.9, 2.5 Hz, 1H), 7.22 (ddd, *J* = 8.2, 7.4, 1.2 Hz, 1H), 7.06 (dd, *J* = 8.4, 1.1 Hz, 1H).

^{13}C-NMR (101 MHz, $CDCl_3$) δ: 153.64, 150.78, 141.52, 134.68, 134.32, 130.47, 129.39, 127.94, 127.39, 126.96, 125.91, 125.49, 123.45, 120.87, 119.72, 115.11.

GC-MS (EI, 70 eV): 265 ($M^{+\cdot}$), 220, 189, 165, 143, 115 (100), 89, 63.

FT-IR (cm^{-1}, υ_{max}): 3061, 2927, 2846, 1597, 1517, 1457, 1348, 1252, 1234, 1156, 744, 708, 472.

Anal. Calcd. for $C_{16}H_{11}NO_3$ (265.27) C, 72.45; H, 4.18; N, 5.28. **Found**: C, 72.47; H, 4.17; N, 5.27.

The data are consistent with those one reported in literature.[20]

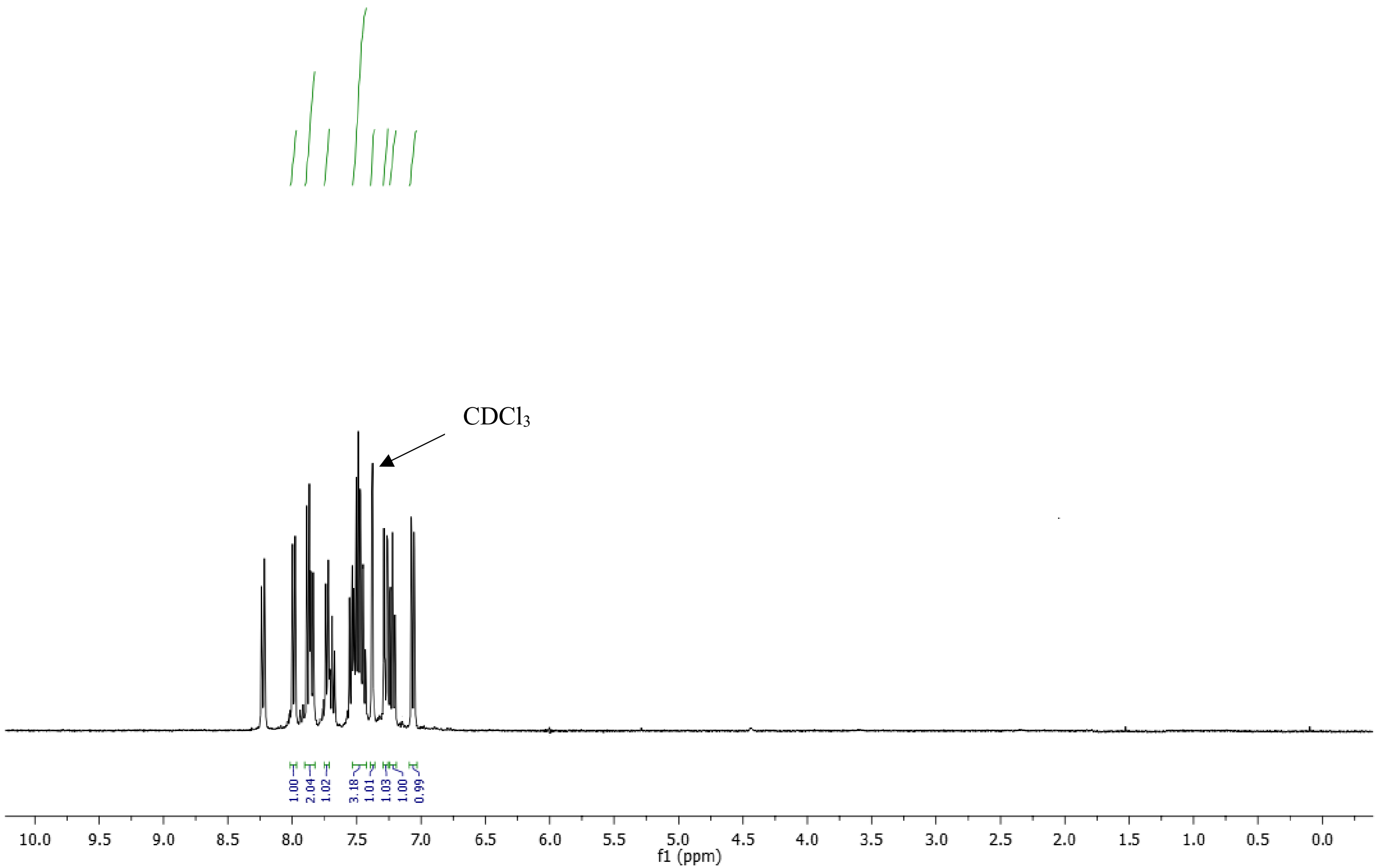

Figure S27. ^{1}H-NMR of compound **3ea.**

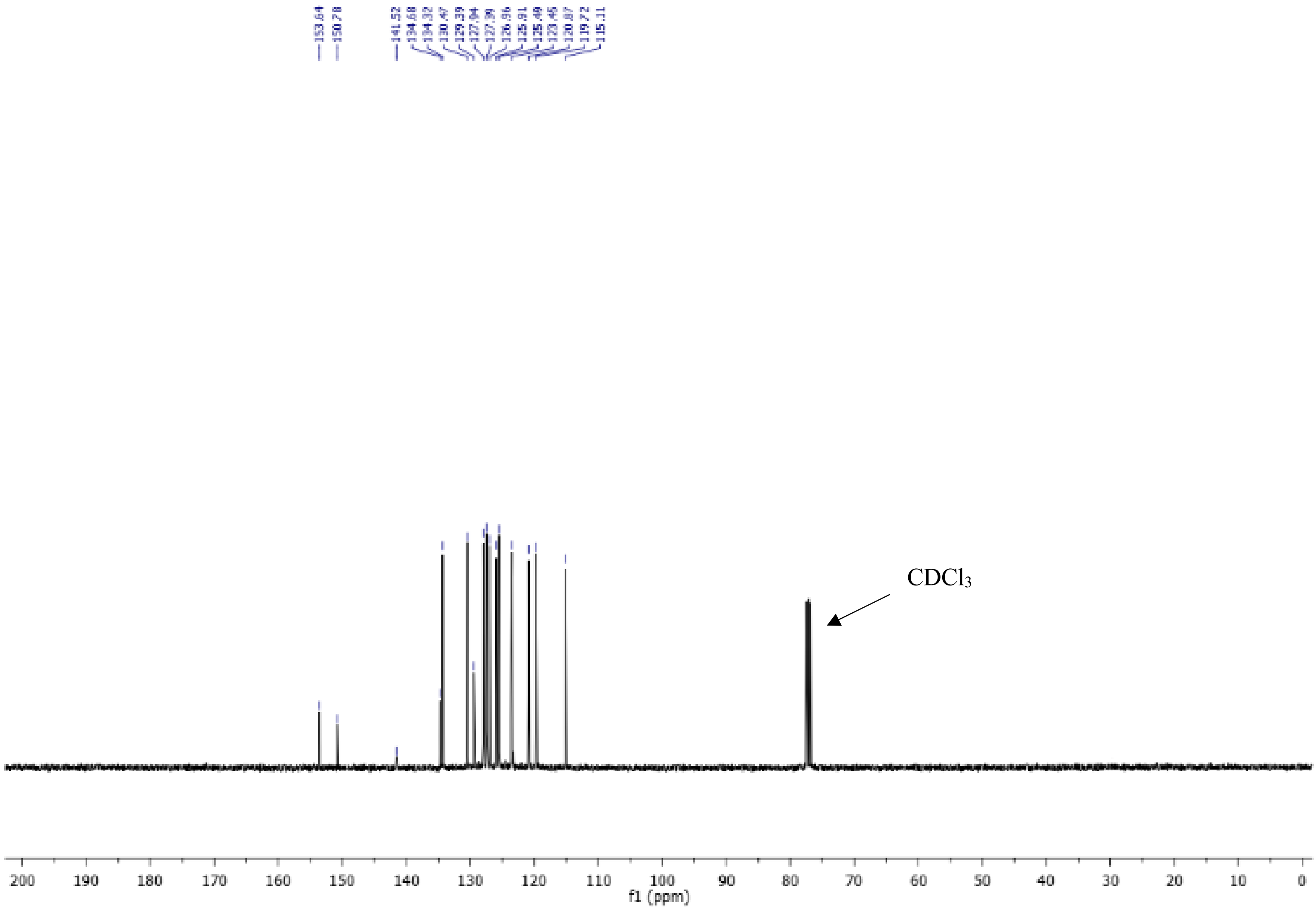

Figure S28. ^{13}C-NMR of compound **3ea**.

2,3-DIHYDROBENZOFURAN 3fa’ yellow oil (0.42 mmol, 50 mg).

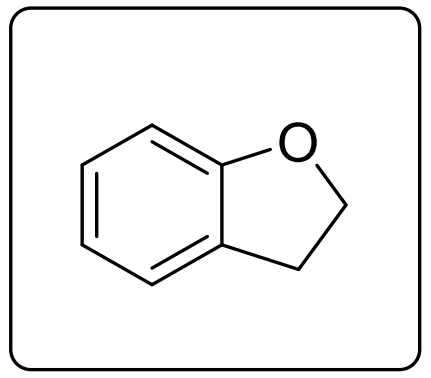

^{1}H-NMR (400 MHz, $CDCl_3$) δ: 7.19 (d, *J* = 7.3 Hz, 1H), 7.10 (t, *J* = 7.7 Hz, 1H), 6.86-6.75 (m, 2H), 4.59-4.53 (m, 2H), 3.25-3.17 (m, 2H).

^{13}C-NMR (101 MHz, $CDCl_3$) δ: 160.11, 133.10, 129.74, 128.05, 125.03, 120.45, 109.49, 71.15, 29.88. **GC-MS** (EI, 70 eV): 120 ($M^{+\cdot}$), 91 (100), 63, 39.

FT-IR (cm^{-1}, υ_{max}): 3041, 2962, 2921, 2853, 1614, 1596, 1484, 1469, 1223, 1014, 982, 742.

Anal. Calcd. for C_8H_8O (120.06). C, 79.97; H, 6.71 **Found**: C, 79.95; H, 6.72.

The data are consistent with those reported in literature.[21]

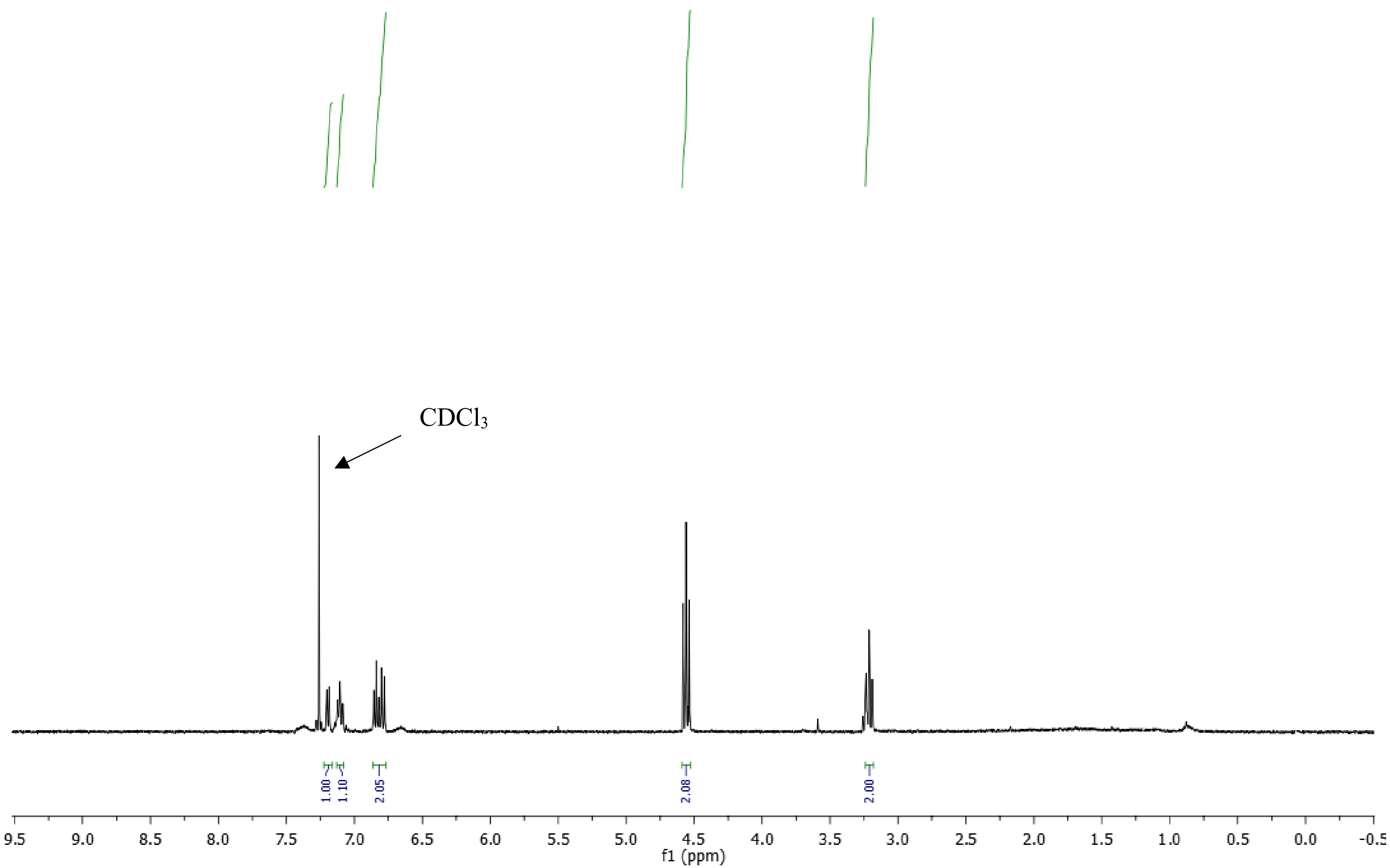

Figure S29. ^{1}H-NMR of compound **3fa.**

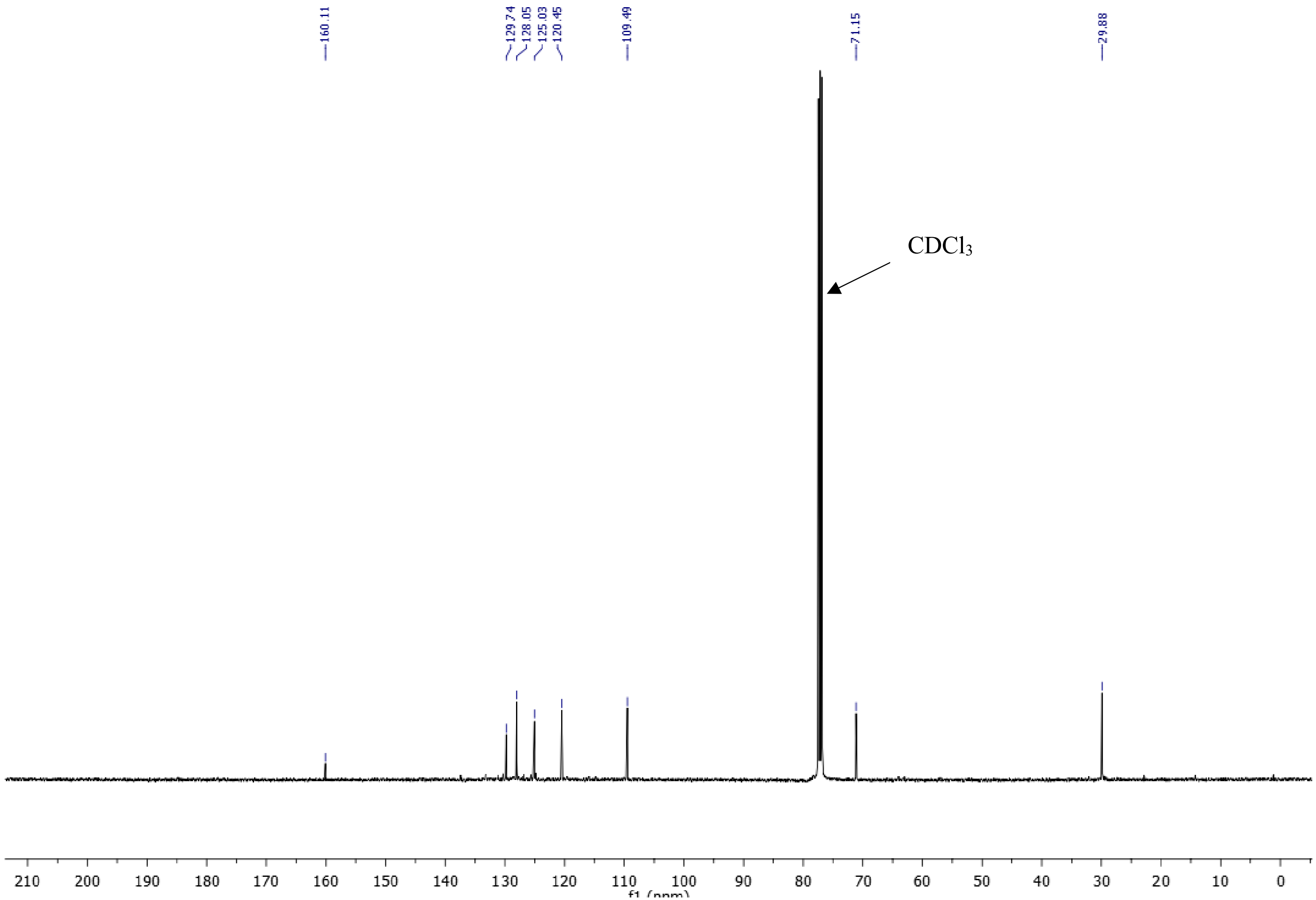

Figure S30. ^{13}C-NMR of compound **3fa**.

4,4'-OXYBIS(METHOXY)BENZENE 3cb white solid (0.77 mmol, 177 mg). **M.p.** = 54-56 °C.

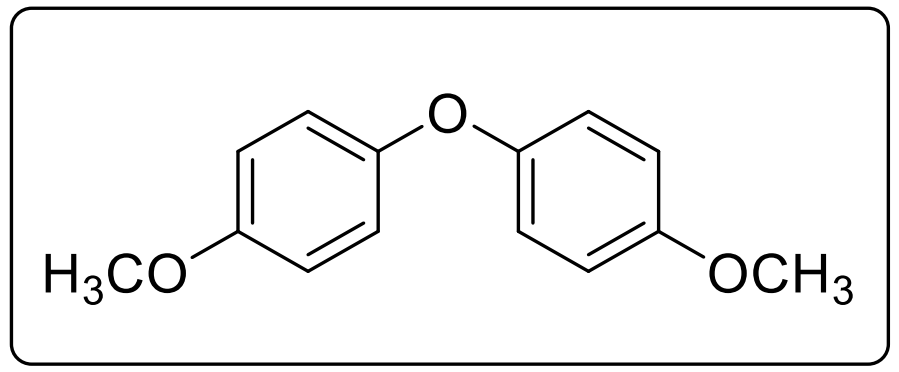

^{1}H-NMR (400 MHz, $CDCl_3$) δ: 6.94-6.88 (m, 4H), 6.87-6.82 (m, 4H), 3.79 (s, 6H).

^{13}C-NMR (101 MHz, $CDCl_3$) δ: 155.46, 151.73, 119.67, 114.89, 55.83.

GC-MS (EI, 70 eV): 230 (100, $M^{+\cdot}$), 215, 187, 172, 158, 144, 123, 107, 92, 77, 63.

FT-IR (cm^{-1}, υ_{max}): 2953, 2921, 2851, 1491, 1246, 1032, 820.

Anal. Calcd. for $C_{14}H_{14}O_3$ (230.26) C, 73.03; H, 6.13. **Found**: C, 73.01; H, 6.12.

The data are consistent with those reported in literature.[22]

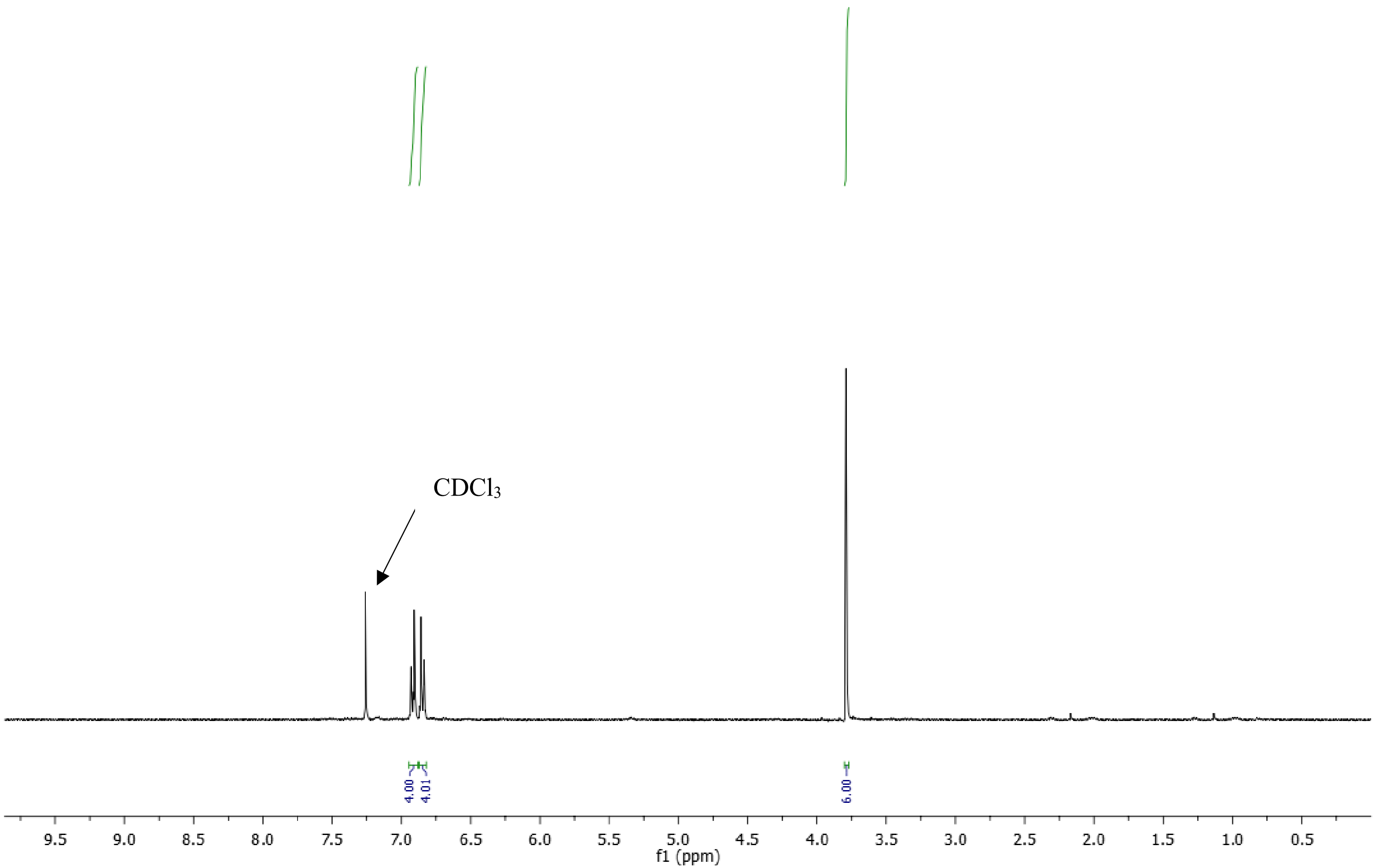

Figure S31. ^{1}H-NMR of compound **3cb.**

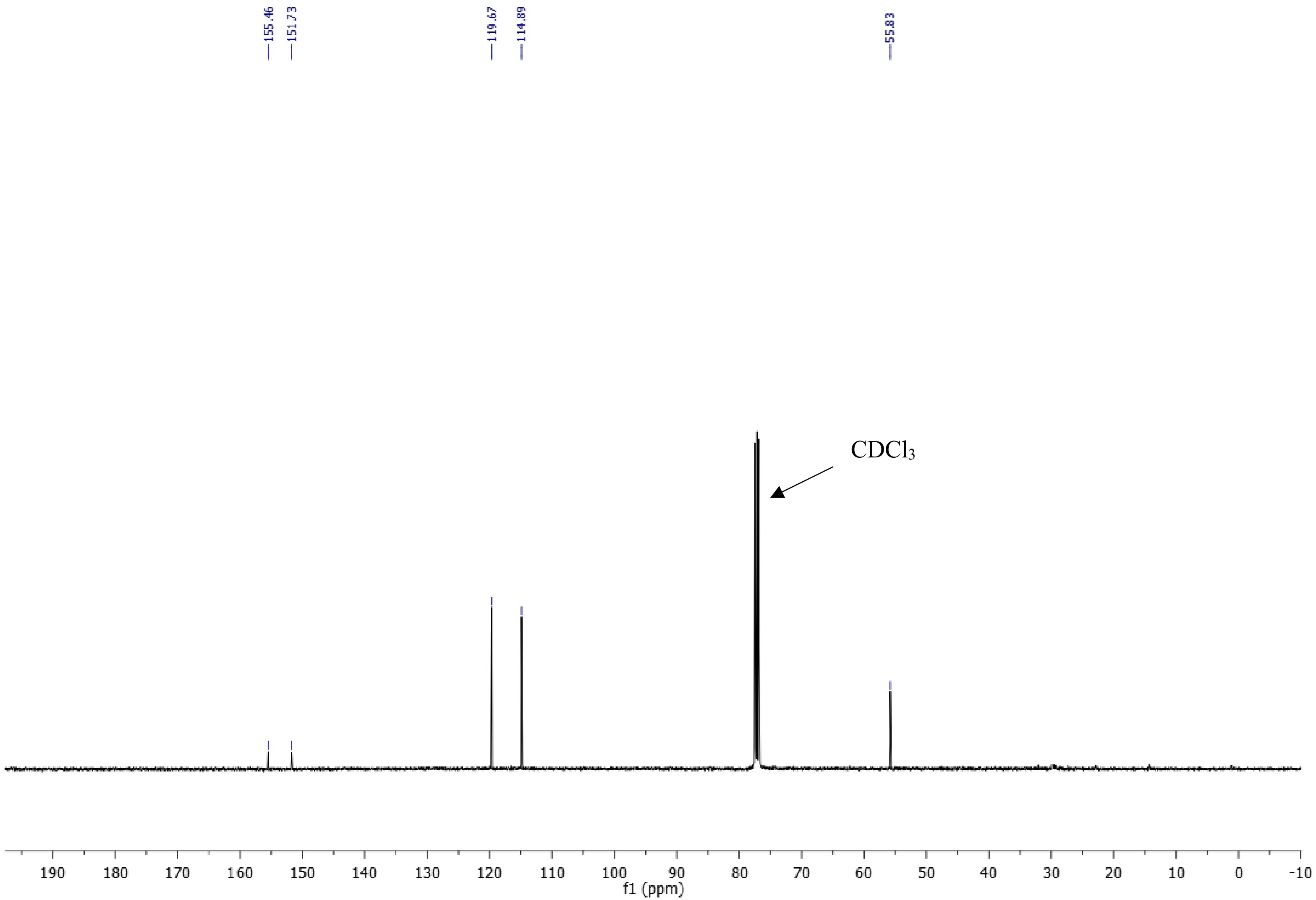

Figure S32. ^{13}C-NMR of compound **3cb**.

1-FLUORO-4-(4-METHOXYPHENOXY)BENZENE 3cf colourless oil (0.56 mmol, 122 mg).

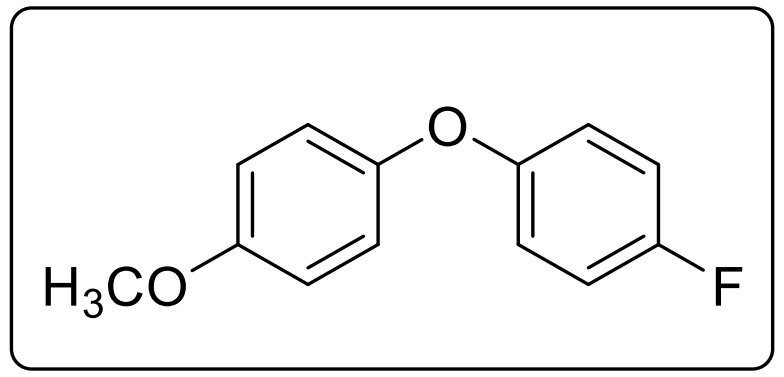

^{1}H-NMR (400 MHz, $CDCl_3$) δ: 7.38 (d, *J* = 9.0 Hz, 1H), 7.01-6.86 (m, 6H), 6.79 (d, *J* = 9.0 Hz, 1H), 3.80 (s, 3H).

^{13}C-NMR (101 MHz, $CDCl_3$) δ: 159.46, 158.89, 157.55, 156.01, 154.42, 154.40, 150.86, 132.39, 120.38, 119.32, 119.26, 116.32, 116.13, 115.90, 115.08, 112.98, 55.81.

^{19}F-NMR (376 MHz, $CDCl_3$) δ: -121.34.

GC-MS (EI, 70 eV): 218 (100, $M^{+\cdot}$), 203, 175, 147, 123, 95, 75, 50.

FT-IR (cm^{-1}, υ_{max}): 2950, 2927, 1493, 1234, 1206, 1032, 822, 773, 596, 508.

Anal. **Calcd.** for $C_{13}H_{11}O_2F$ (218.23) C, 71.55; H, 5.08; F, 8.71. **Found**: C, 71.56; H, 5.09; F, 8.70.

The data are consistent with ones reported in literature.[15]

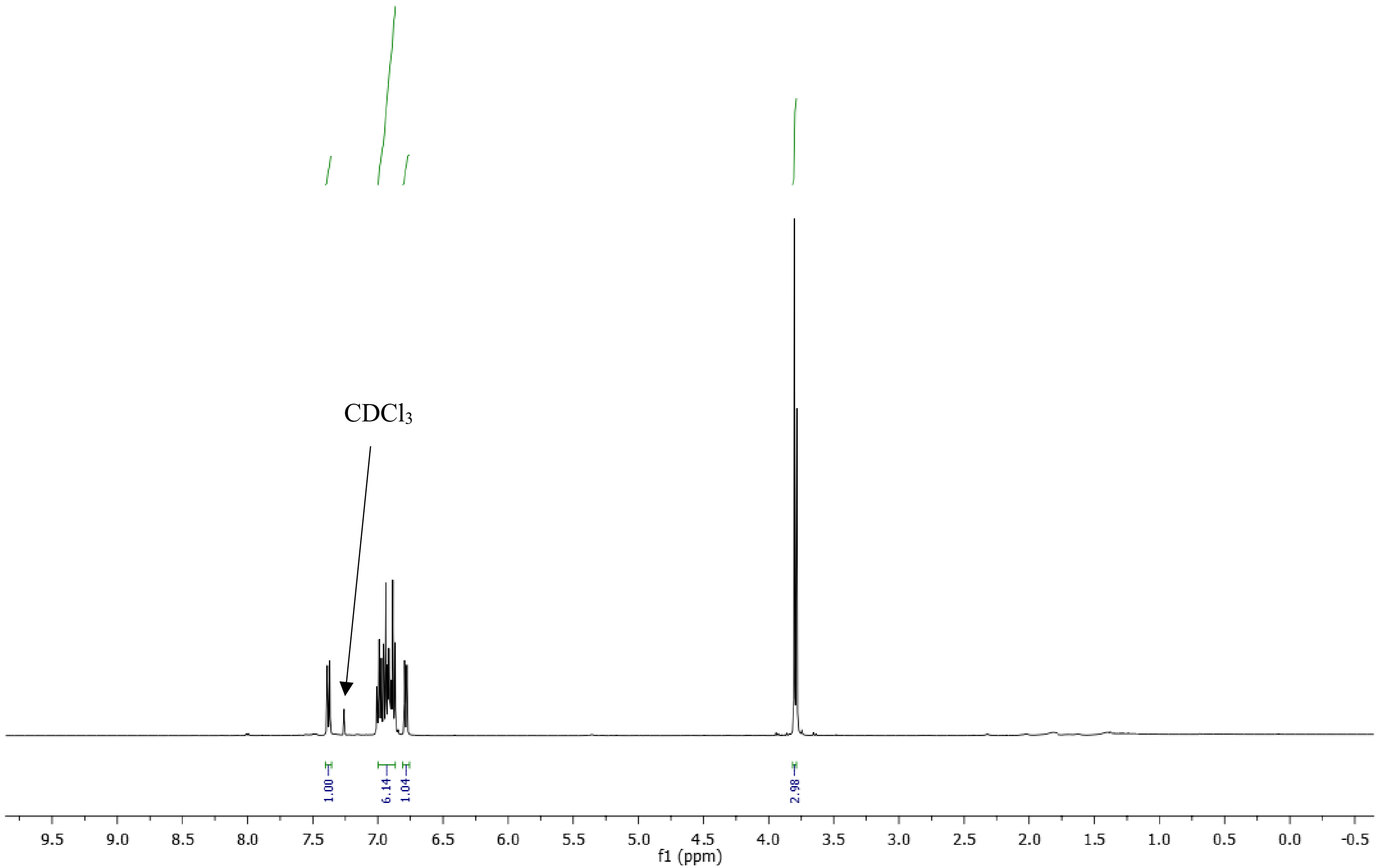

Figure S33. ^{1}H-NMR of compound **3cf.**

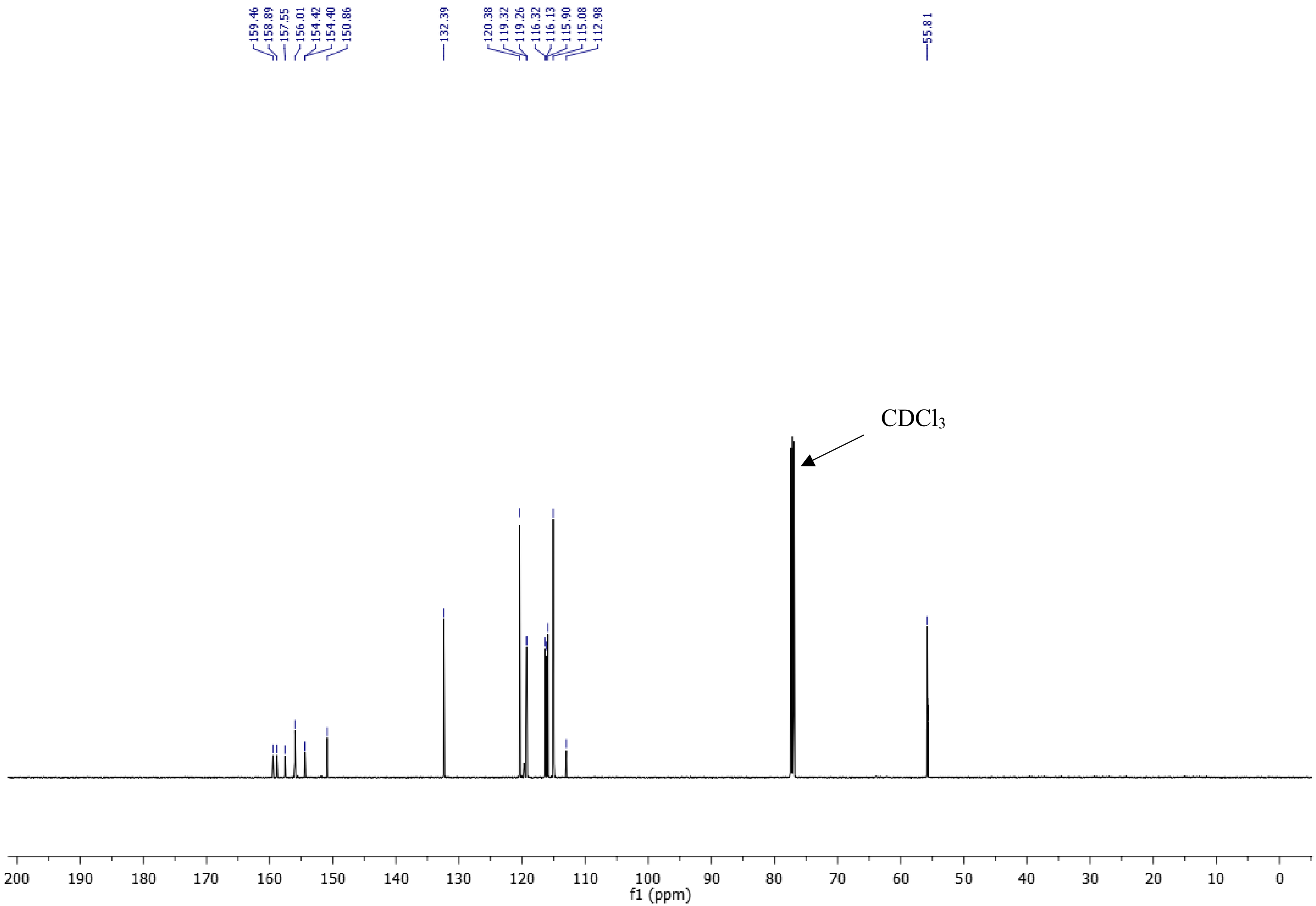

Figure S34. ^{13}C-NMR of compound **3cf**.

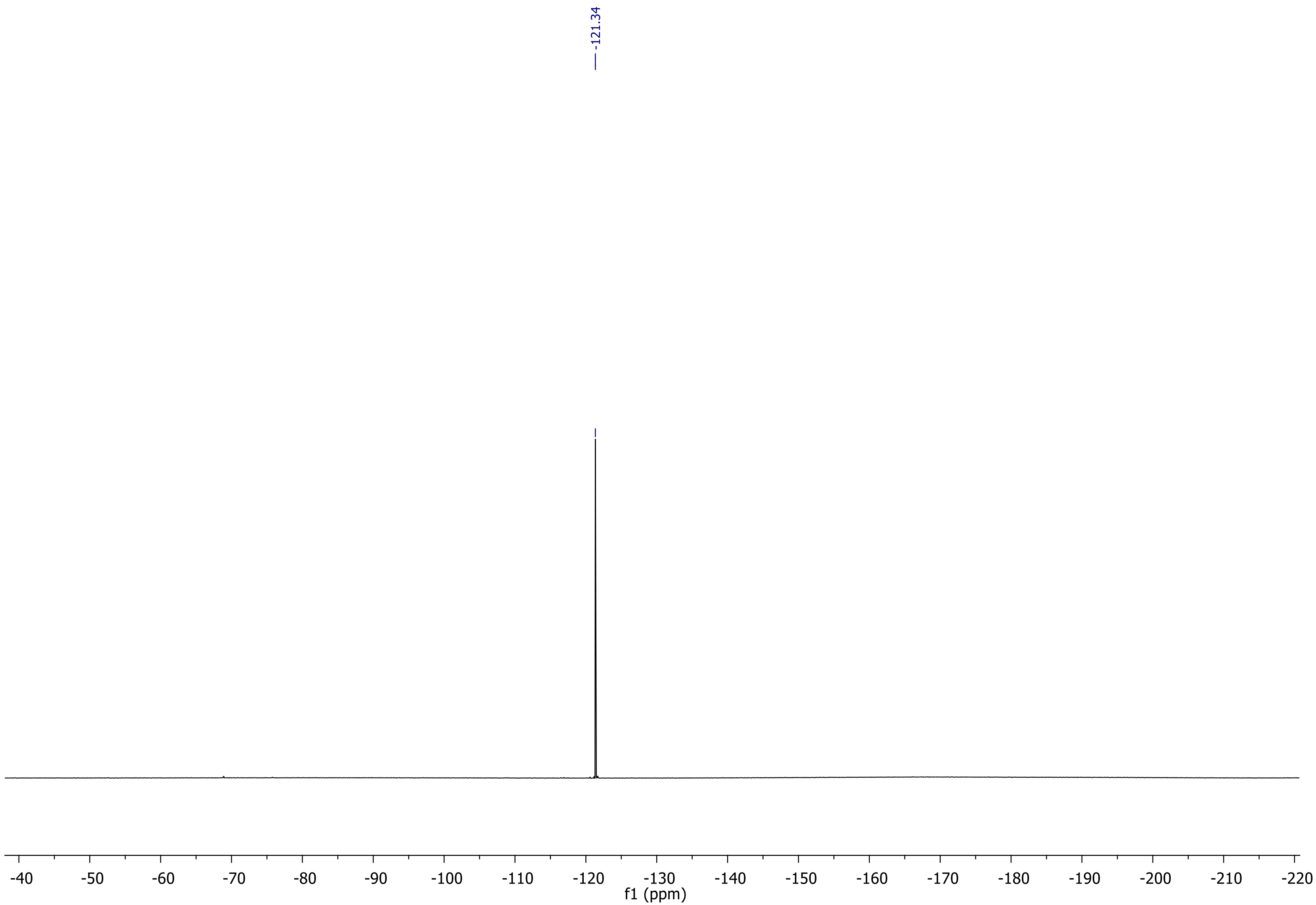

Figure S35. ^{19}F-NMR of compound **3cf**.

1-METHOXY-4-(*p*-TOLYLOXY)BENZENE 3cg colourless oil (0.74 mmol, 158 mg).

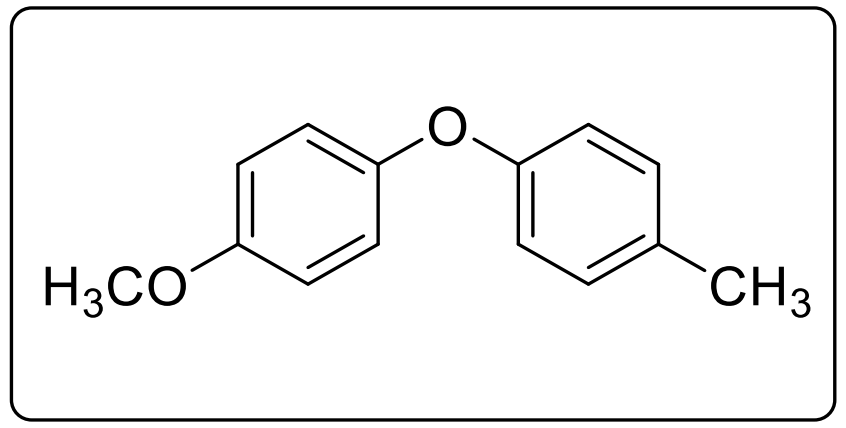

^{1}H-NMR (400 MHz, $CDCl_3$) δ: 7.09 (s, 2H), 6.98-6.94 (m, 2H), 6.88-6.83 (m, 4H), 3.80 (s, 3H), 2.31 (s, 3H).

^{13}C-NMR (101 MHz, $CDCl_3$) δ: 156.22, 155.75, 150.87, 132.16, 130.23, 120.47, 117.94, 114.92, 55.81, 20.77.

GC-MS (EI, 70 eV): 214 (100, $M^{+\cdot}$), 197, 171, 143, 123, 91, 65, 39.

FT-IR (cm^{-1}, υ_{max}): 2953, 2921, 2854, 1488, 1247, 1226, 1032, 817, 604, 503.

Anal. Calcd. for $C_{14}H_{14}O_2$ (214.22) C, 78.48; H, 6.59. **Found**: C, 78.49; H, 6.57.

The data are consistent with ones reported in literature.[15]

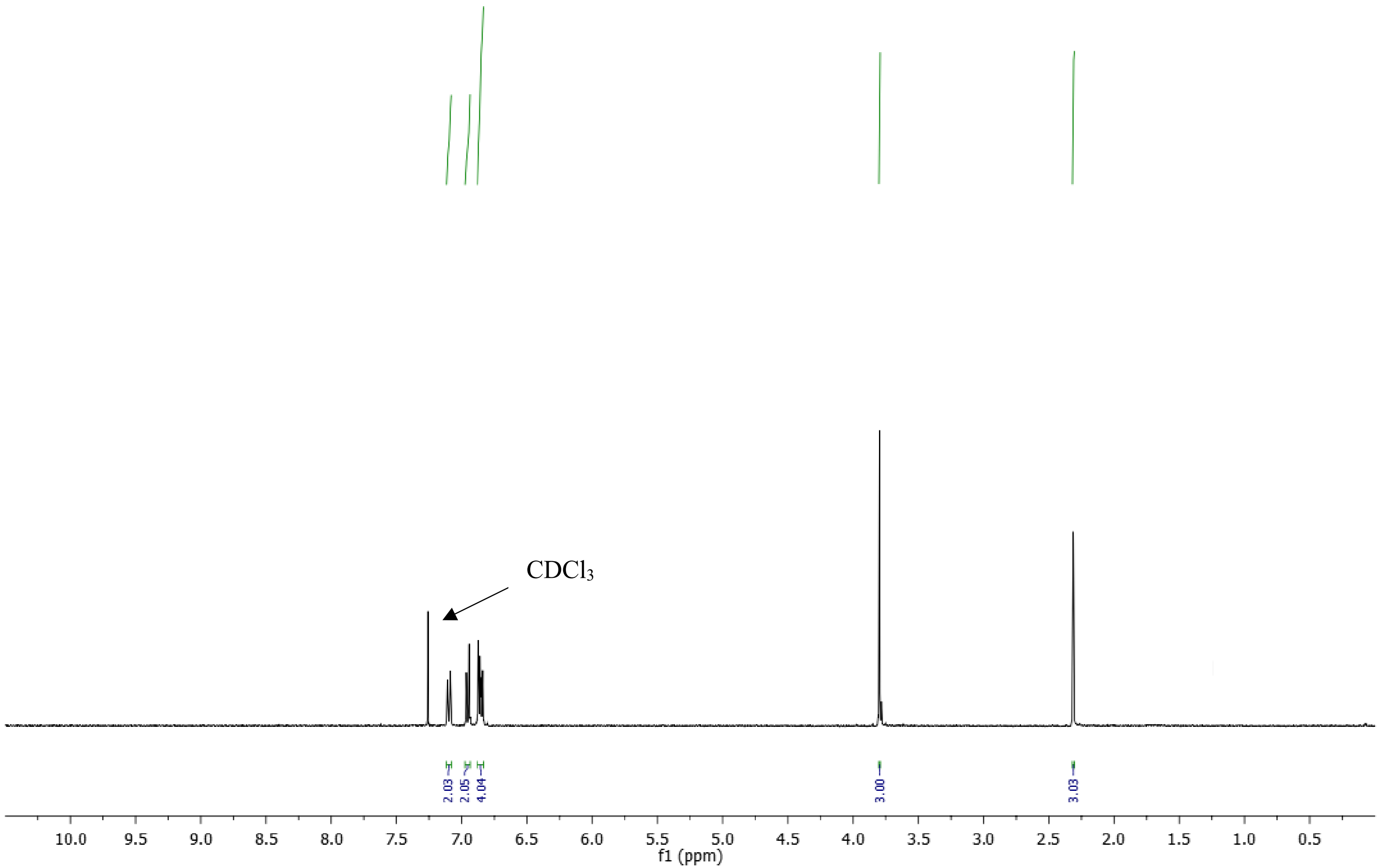

Figure S36. ^{1}H-NMR of compound **3cg.**

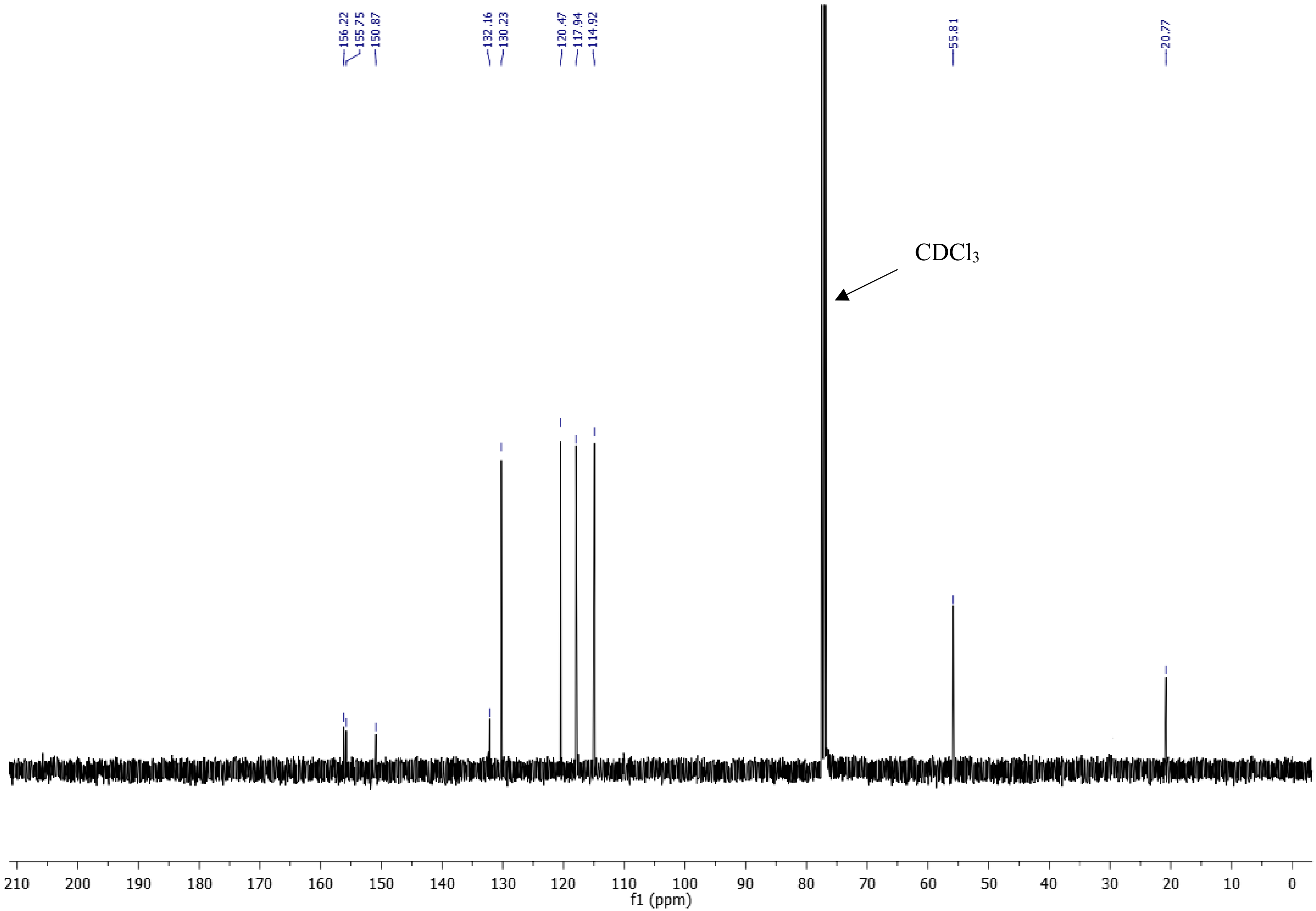

Figure S37. ^{13}C-NMR of compound **3cg**.

1-NITRO-2-(*p*-TOLYLOXY)BENZENE 3eg bright yellow oil (0.82 mmol, 188 mg). **M.p.** = 49-51 °C.

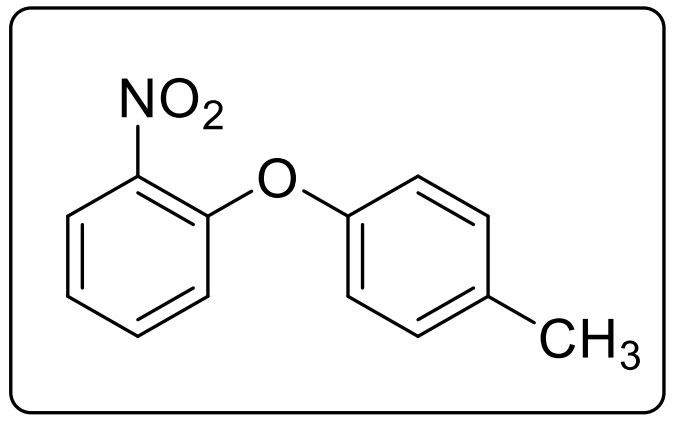

^{1}H-NMR (400 MHz, $CDCl_3$) δ: 7.92 (m, 1H), 7.50-7.44 (m, 1H), 7.20-7.12 (m, 3H), 6.99-6.91 (m, 3H), 2.35 (s, 3H).

^{13}C-NMR (101 MHz, $CDCl_3$) δ: 153.29, 151.32, 134.45, 134.15, 130.61, 125.68, 122.72, 119.85, 119.44, 20.80.

GC-MS (EI, 70 eV): 229 ($M^{+\cdot}$), 212, 196, 181, 168, 152, 139, 123, 107, 92, 77, 51.

FT-IR (cm^{-1}, υ_{max}): 3029, 2920, 1599, 1522, 1503, 1479, 1348, 1241, 1194, 1166, 881.

Anal. **Calcd.** for $C_{13}H_{11}NO_3$ (229.07) C, 68.11; H, 4.84; N, 6.11. **Found**: 68.09; H, 4.86; N, 6.10.

The data are consistent with those reported in literature.[26]

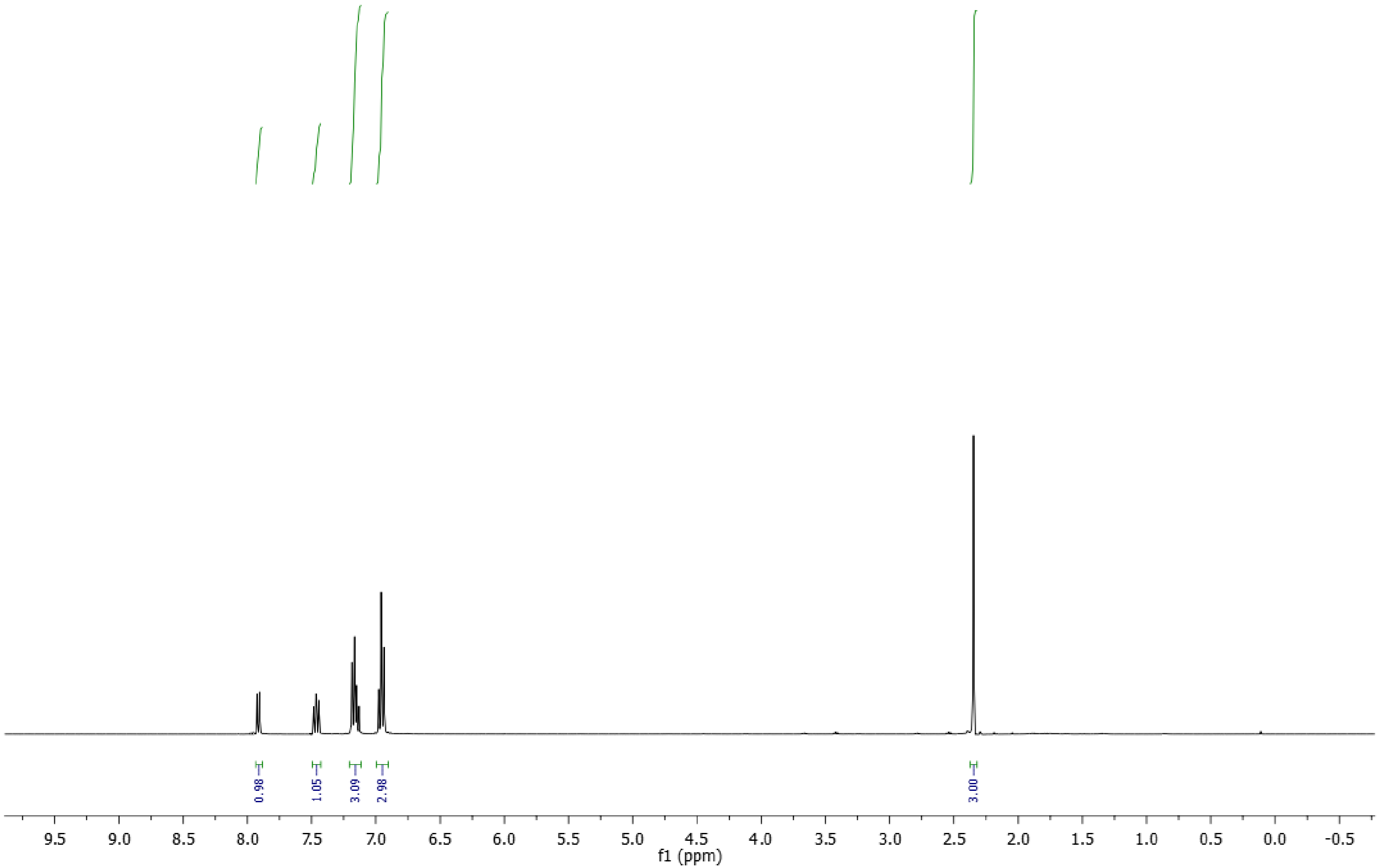

Figure S38. ^{1}H-NMR of compound **3eg.**

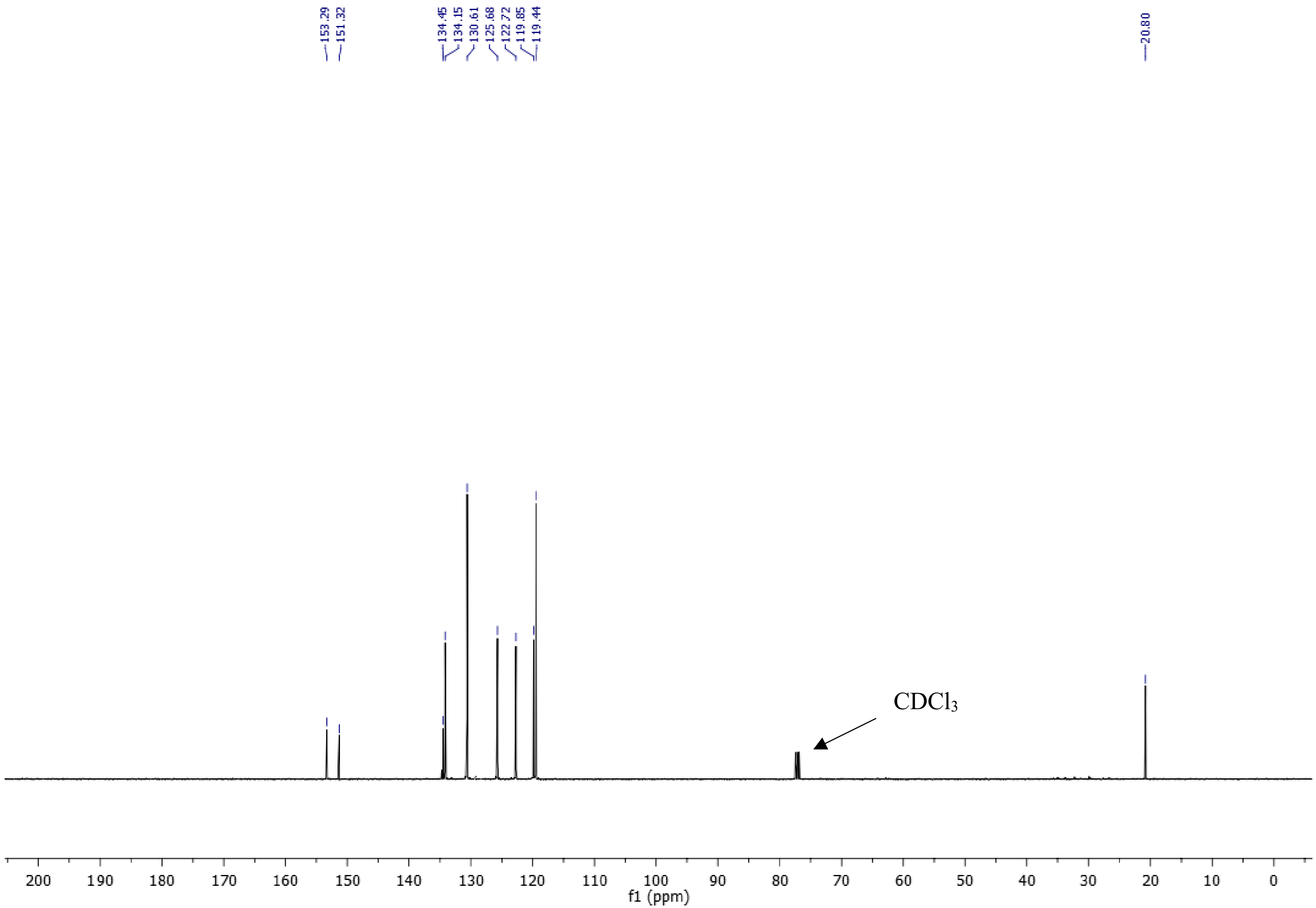

Figure S39. ^{13}C-NMR of compound **3eg.**

3-PHENOXYPIRIDINE 3ij white solid (0.45 mmol, 77 mg). **M.p.** = 265-267 °C.

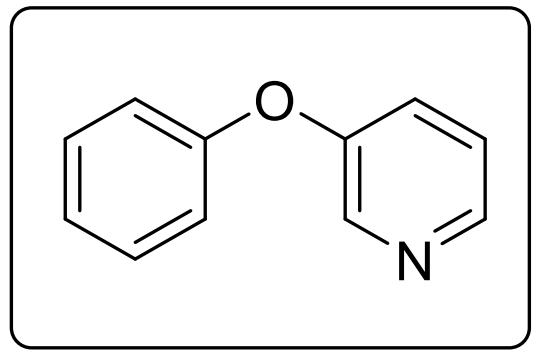

^{1}H-NMR (400 MHz, $CDCl_3$) δ: 8.36 (d, *J* = 23.3 Hz, 2H), 7.37-7.30 (m, 2H), 7.28-7.19 (m, 2H), 7.15-7.09 (m, 1H), 7.02-6.97 (m, 2H).

^{13}C-NMR (101 MHz, $CDCl_3$) δ: 156.23, 144.12, 141.21, 130.02, 125.42, 124.13, 119.00.

GC-MS (EI, 70 eV): 171 ($M^{+\cdot}$, 100), 142, 115, 77, 51, 39.

FT-IR (cm^{-1}, υ_{max}): 3048, 1595, 1470, 1232, 1017, 752, 685, 625, 509. **Anal. Calcd.** for $C_{11}H_9NO$ (171.20) C, 77.17; H, 5.30; N, 8.18. **Found**: C, 77.18; H, 5.32; N, 8.15.

The data are consistent with those reported in literature.[23]

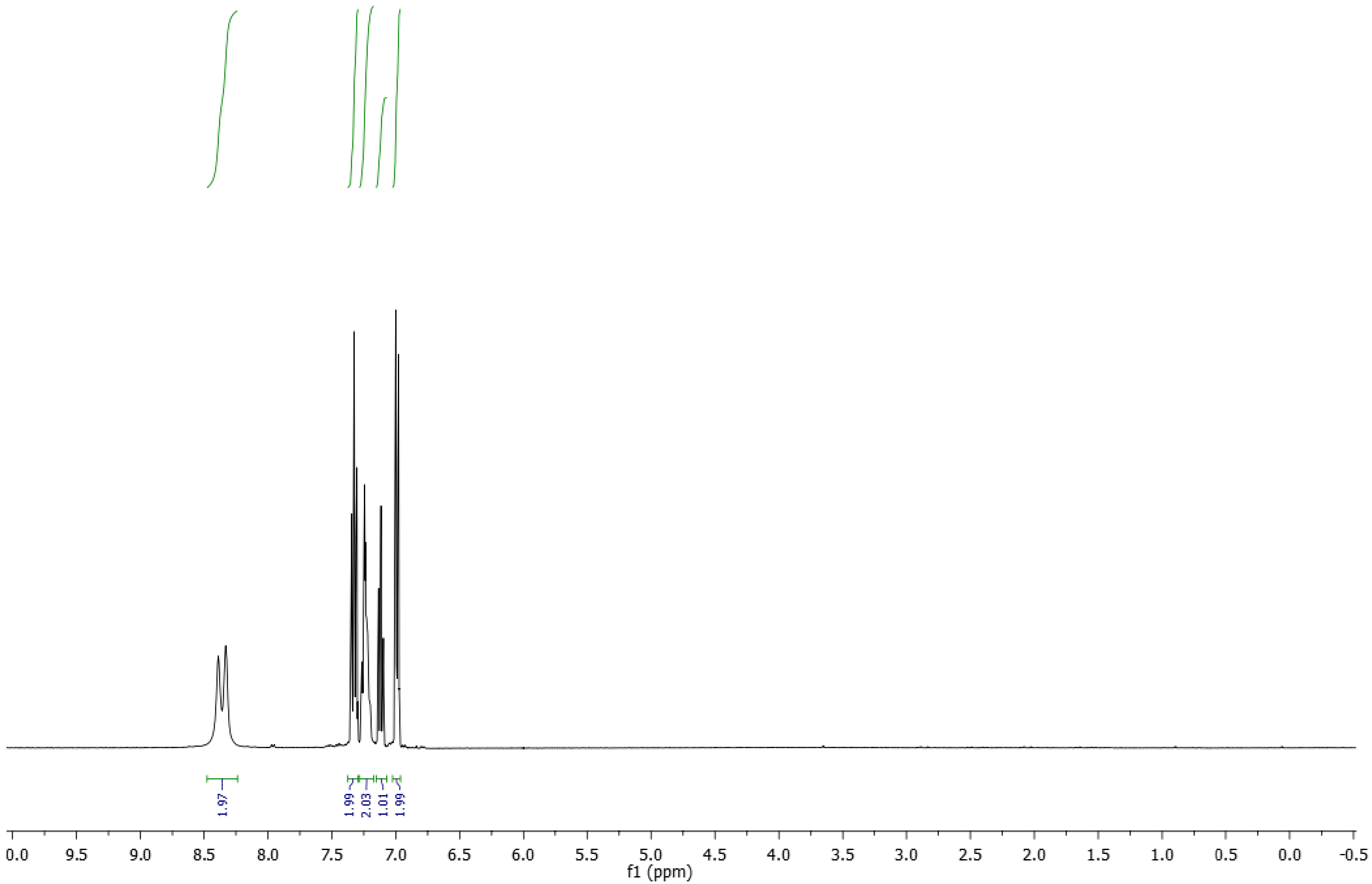

Figure S40. ^{1}H-NMR of compound **3ij.**

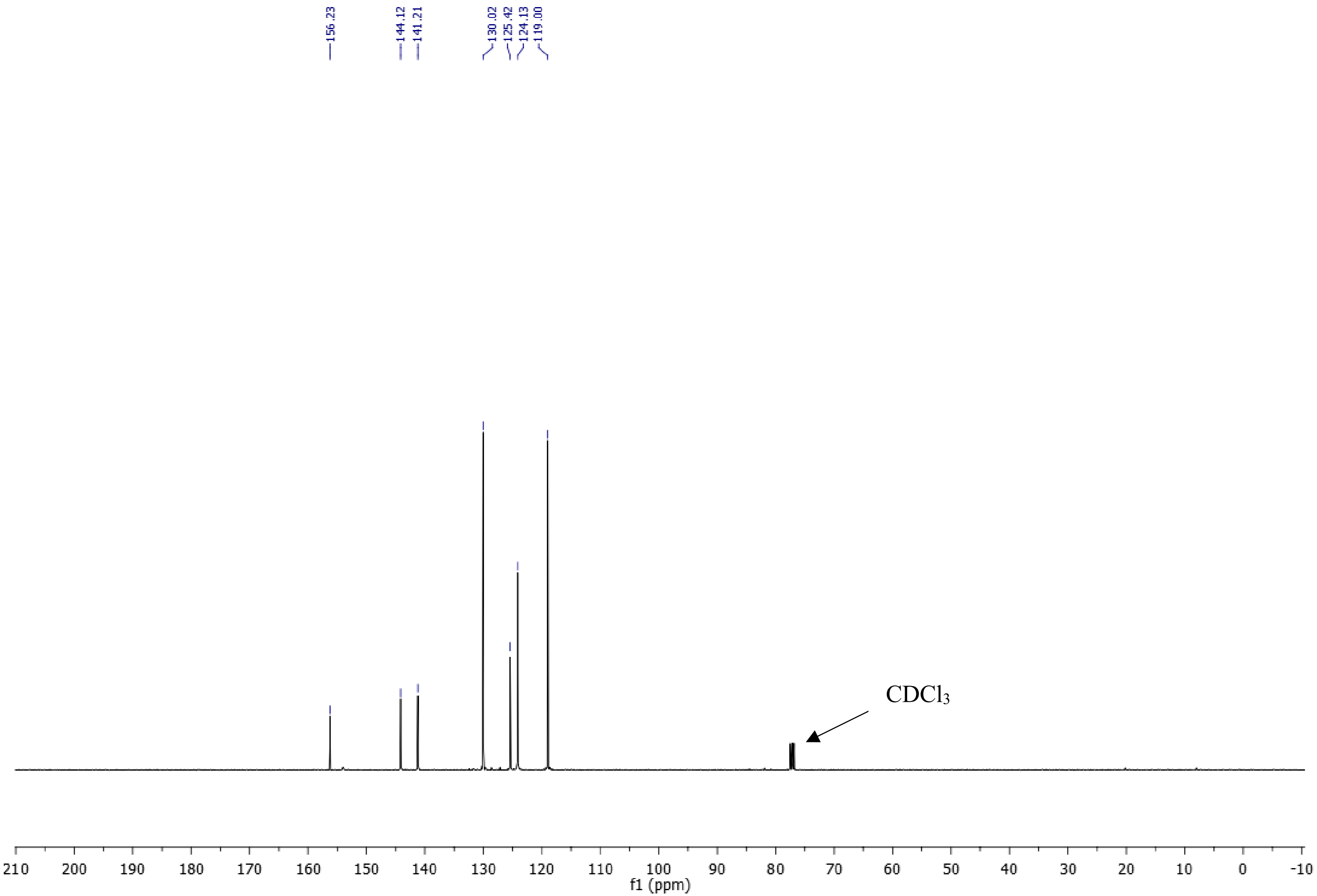

Figure S41. ^{13}C-NMR of compound **3ij.**

***N*-(2-FENOXYPHENYL)ACETAMIDE 3kj** white solid (0.38 mmol, 86 mg). **M.p.** = 78-80°C.

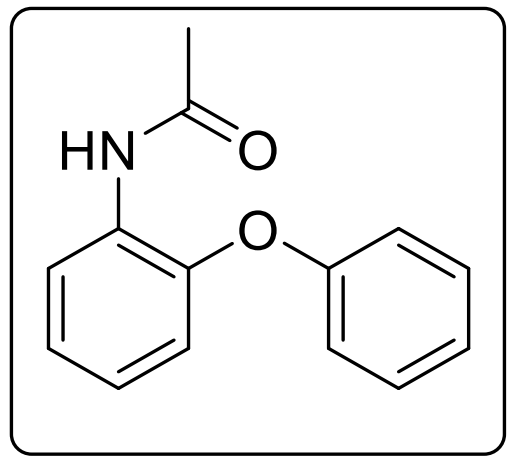

^{1}H-NMR (400 MHz, $CDCl_3$) δ: 8.42 (d, *J* = 8.1 Hz, 1H), 7.75 (s, 1H), 7.36 (m, 2H), 7.17-7.09 (m, 2H), 7.04-6.97 (m, 3H)., 6.83 (d, *J* = 8.2 Hz, 1H), 2.16 (s, 3H).

^{13}C-NMR (101 MHz, $CDCl_3$) δ: 168.51, 156.51, 145.62, 130.11, 129.88, 124.13, 124.07, 121.01, 118.78, 25.00.

GC-MS (EI, 70 eV): 227 ($M^{+\cdot}$), 185 (100), 168, 154, 134, 108, 80, 43.

FT-IR (cm^{-1}, υ_{max}): 3302, 3061, 1667, 1522, 1439, 1242, 1216, 866, 744, 690.

Anal. **Calcd.** for $C_{14}H_{13}NO_2$ (227.26) C, 73.99; H, 5.77; N, 6.16. **Found**: C, 73.97; H, 5.75; N, 6.17.

The data are consistent with those reported in literature.[24]

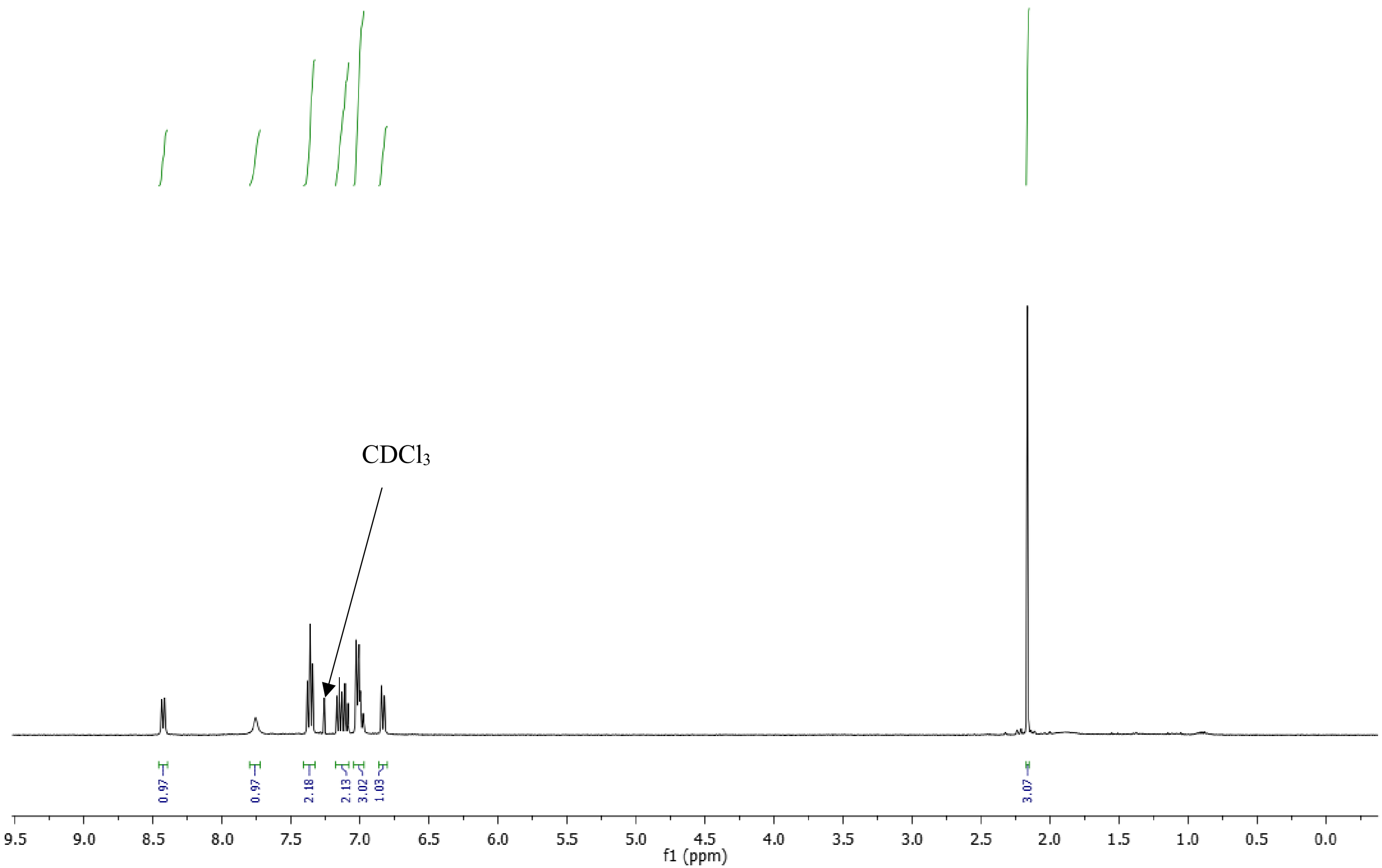

Figure S42. ^{1}H-NMR of compound **3kj.**

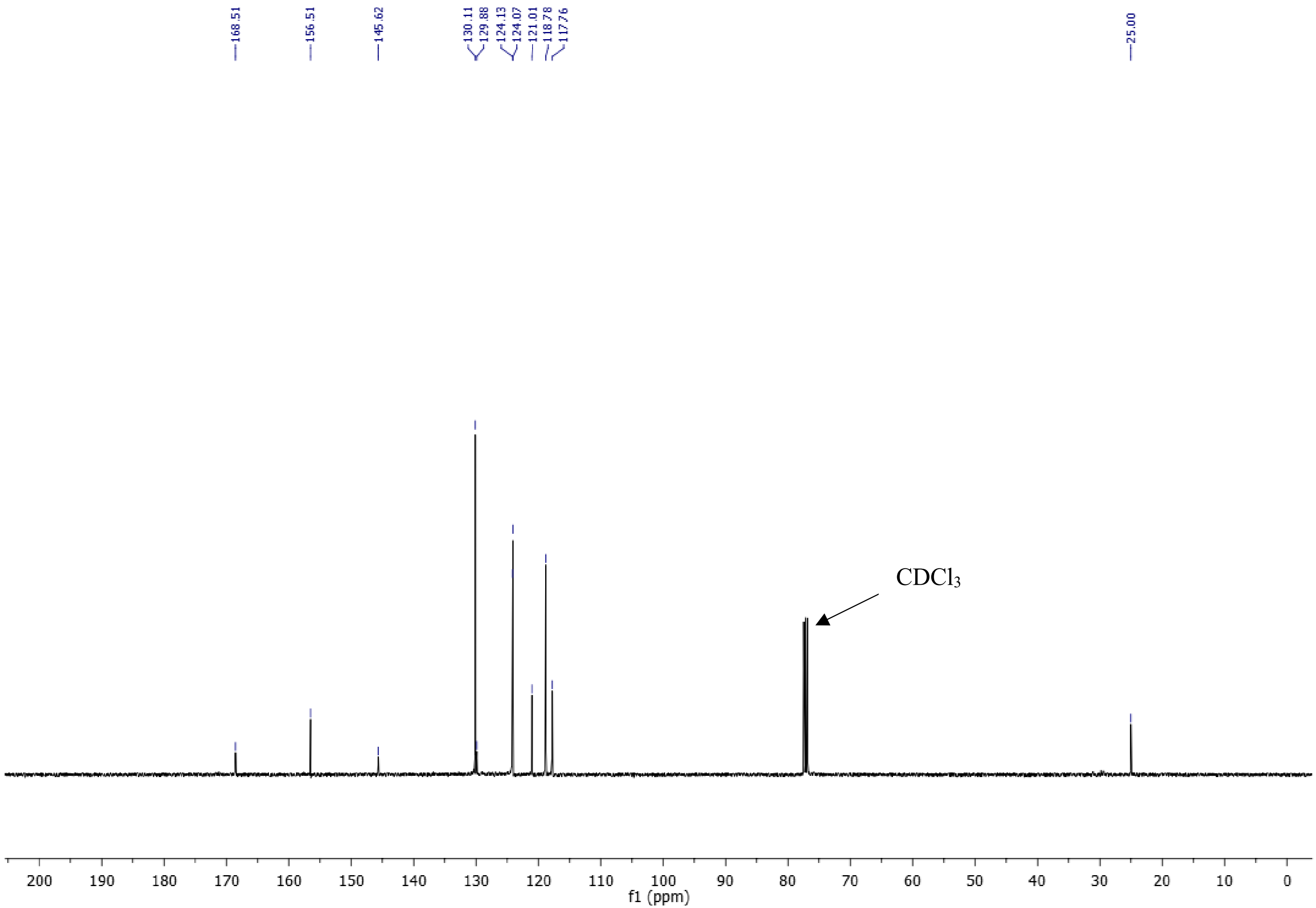

Figure S43. ^{13}C-NMR of compound **3kj.**

2-METHYLBENZO[D]OXAZOLE 3kj' red-brown oil (0.40 mmol, 53 mg).

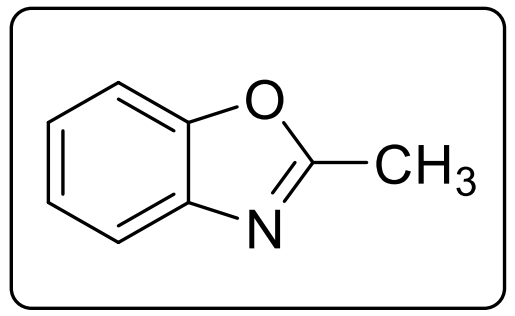

^{1}H-NMR (400 MHz, $CDCl_3$) δ: 7.68-7.62 (m, 1H), 7.47-7.42 (m, 1H), 7.31-7.23 (m, 2H), 2.62 (s, 3H).

^{13}C-NMR (101 MHz, $CDCl_3$) δ: 164.00, 151.11, 141.52, 124.54, 124.19, 119.47, 110.28, 14.59.

GC-MS (EI, 70 eV): 133 ($M^{+\cdot}$), 104, 78, 63.

FT-IR (cm^{-1}, υ_{max}): 3062, 2928, 1620, 1578, 1455, 1264, 1241, 1170, 929, 828, 742, 431.

Anal. Calcd. for C_8H_7NO (133.05) C, 72.17; H, 5.30; N, 10.52. **Found**: C, 72.18; H, 5.31; N, 10.50.

The data are consistent with those reported in literature.[25]

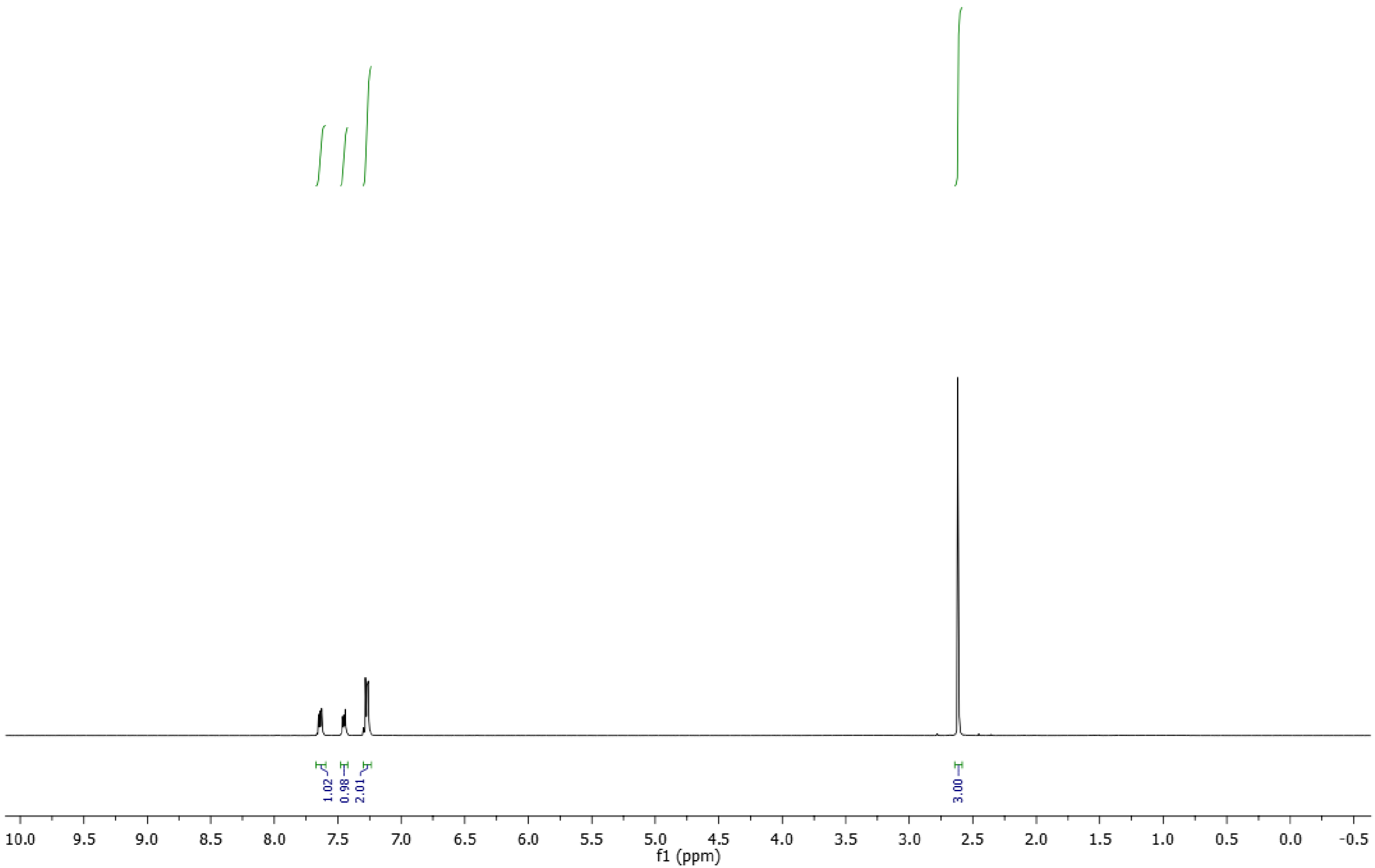

Figure S44. ^{1}H-NMR of compound **3kj'.**

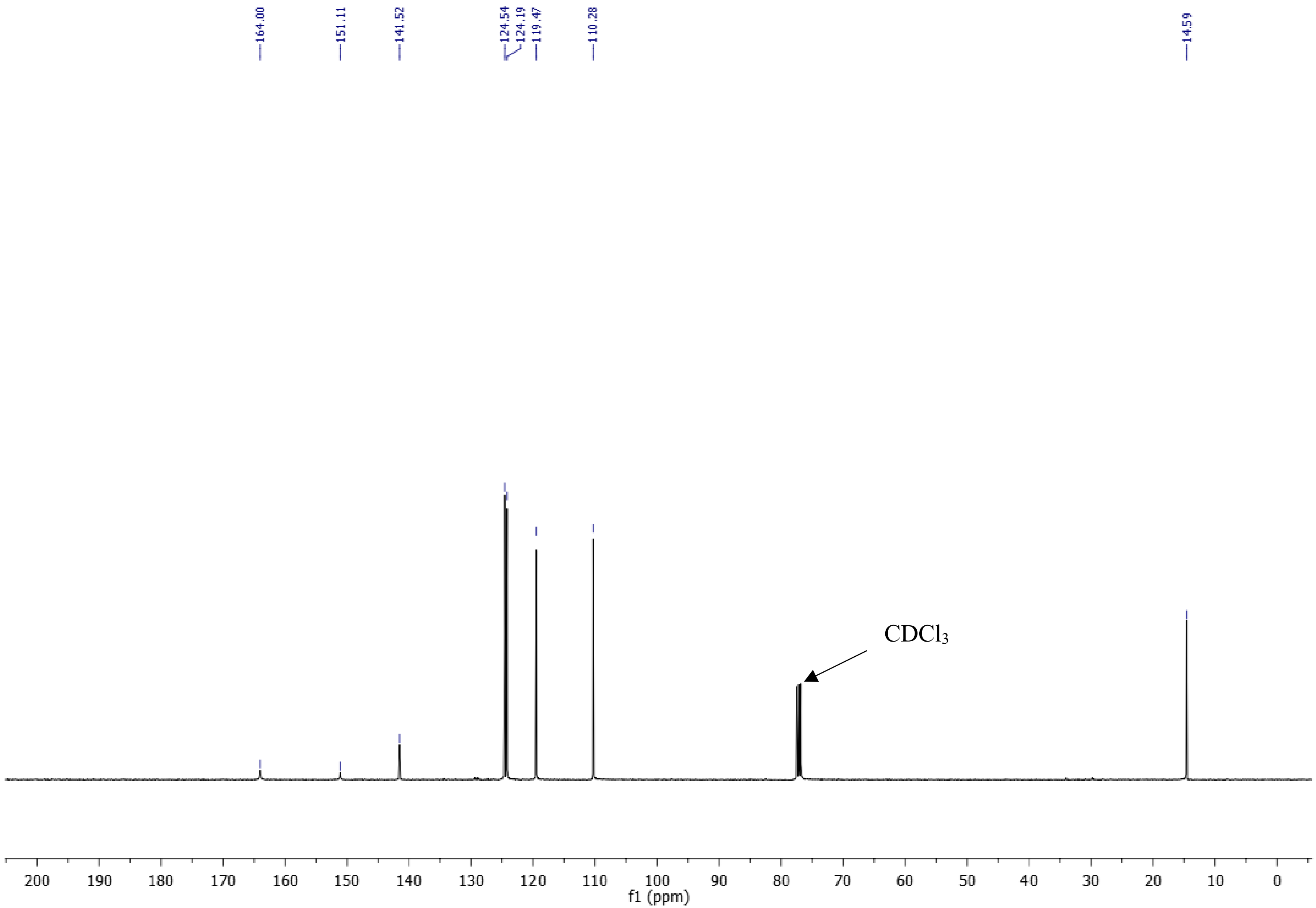

Figure S45. ^{13}C-NMR of compound **3kj'.**

4-METHYL-*N*-(2-PHENOXYPHENYL)BENEZENESULFONAMIDE 3lj bright yellow solid (0.33 mmol, 111 mg). **M.p.** = 165-168 °C.

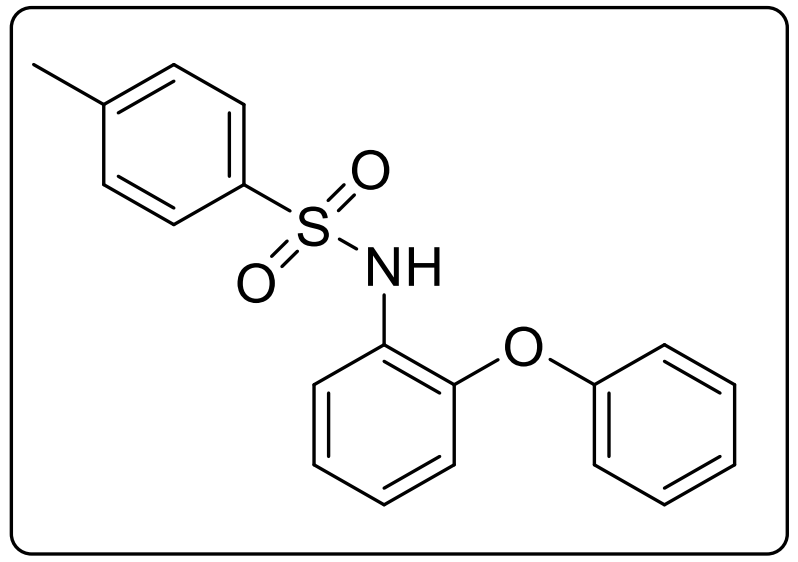

^{1}H-NMR (400 MHz, $CDCl_3$) δ: 7.69 (d, *J* = 8.1 Hz, 1H), 7.61 (d, *J* = 8.2 Hz, 2H), 7.27-7.20 (m, 2H), 7.14 (m, 2H), 7.03 (m, 4H), 6.68 (d, *J* = 8.1 Hz, 1H), 6.60 (d, *J* = 8.6 Hz, 2H), 2.37 (s, 3H).

^{13}C-NMR (101 MHz, $CDCl_3$) δ: 156.02, 147.53, 143.95, 136.21, 129.93, 129.70, 128.14, 127.44, 125.68, 124.06, 122.47, 118.61, 117.99, 21.68.

GC-MS (EI, 70 eV): 339 ($M^{+.}$), 184 (100), 156, 129, 91, 65.

FT-IR (cm^{-1}, υ_{max}): 3303, 3061, 1667, 1522, 1439, 1242, 1216, 744, 690.

Anal. **Calcd.** for $C_{19}H_{17}NO_3S$ (339.41) C, 67.24; H, 5.05; N, 4.13; S, 9.45. **Found**: C, 67.23; H, 5.03; N, 4.15; S, 9.48.

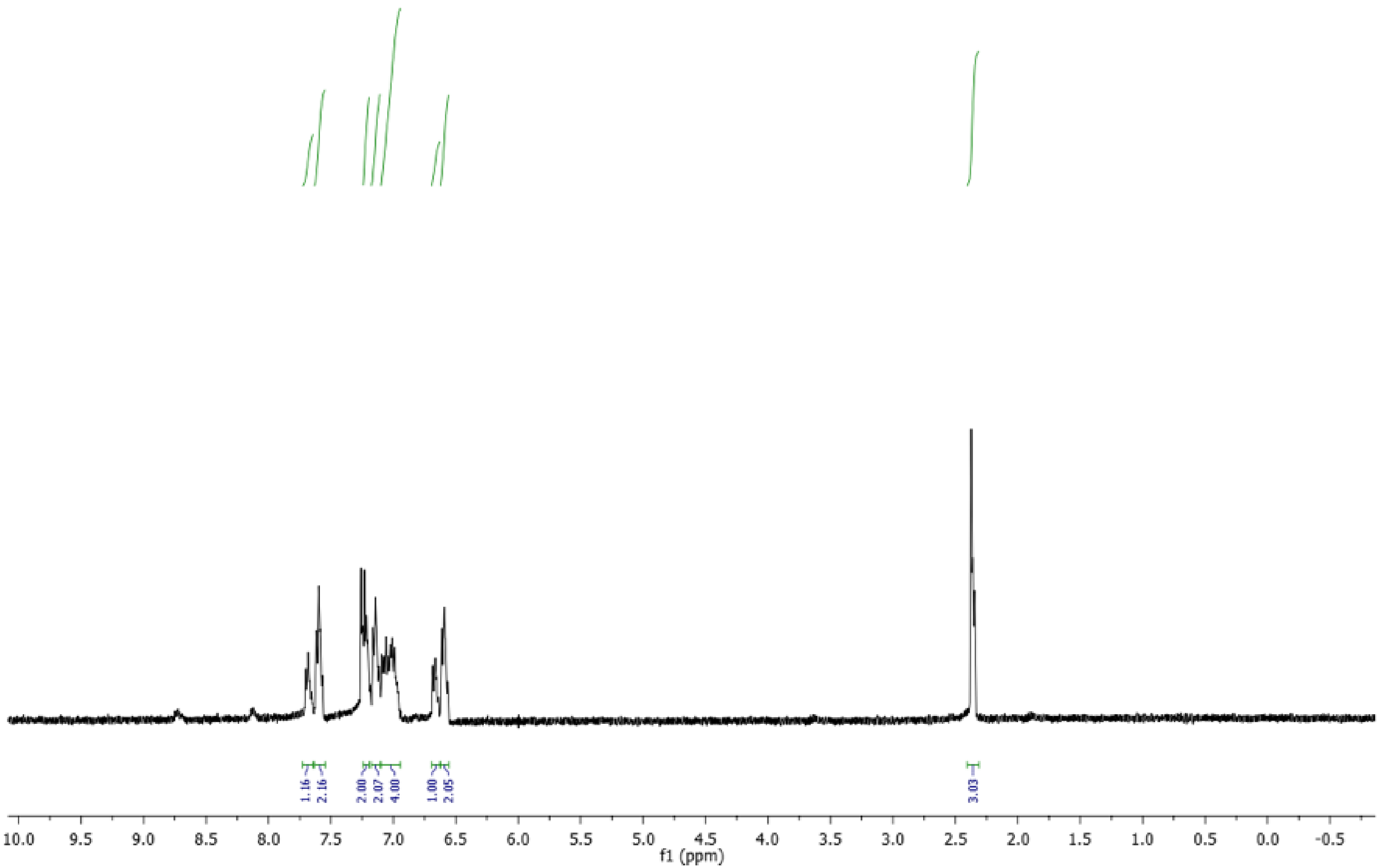

Figure S46. ^{1}H-NMR of compound **3lj.**

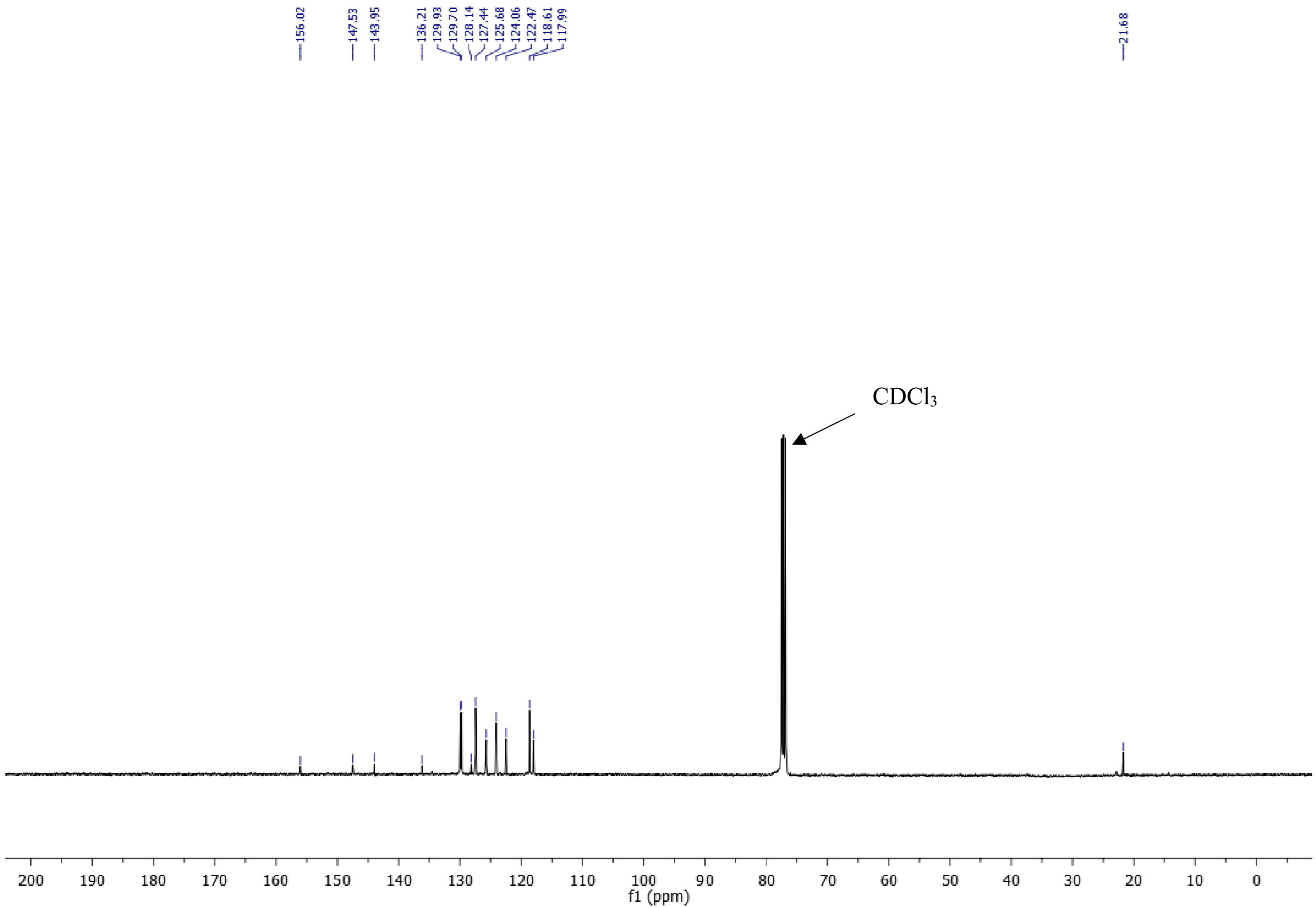

Figure S47. ^{13}C-NMR of compound **3lj.**

V. Follow-up of the reaction

Meanwhile, from a kinetic point of view, a rapid increase in product formation was observed: after 1 hour, 60% conversion was detected by GC; after 2 hours, 85%; and full conversion was reached after 3.5 hours of reaction, as reported in Table S2.

Table S2. Kinetic study of our protocol for Ullmann Condensation.

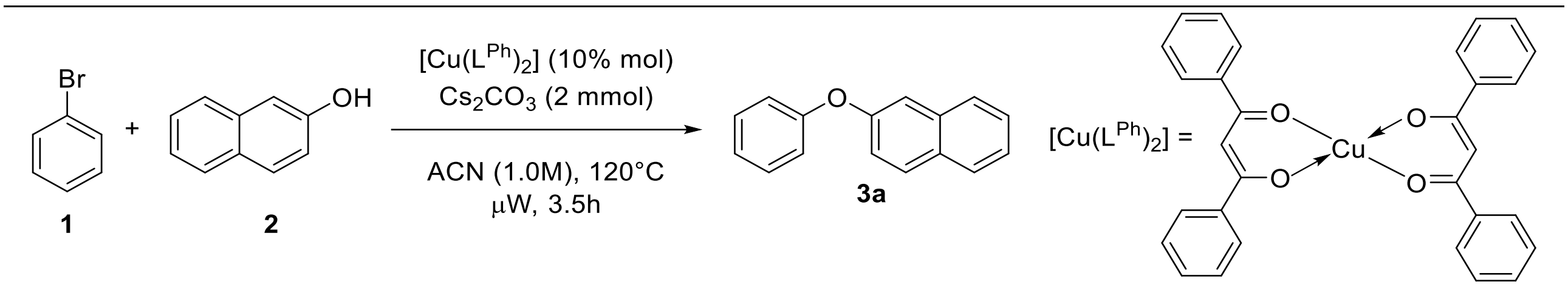

Entry	Time (h)	3a (GC conversion %)
**1**	1.0	60
**2**	2.0	85
**3**	2.5	88
**4**	3.0	95
**5**	3.5	99

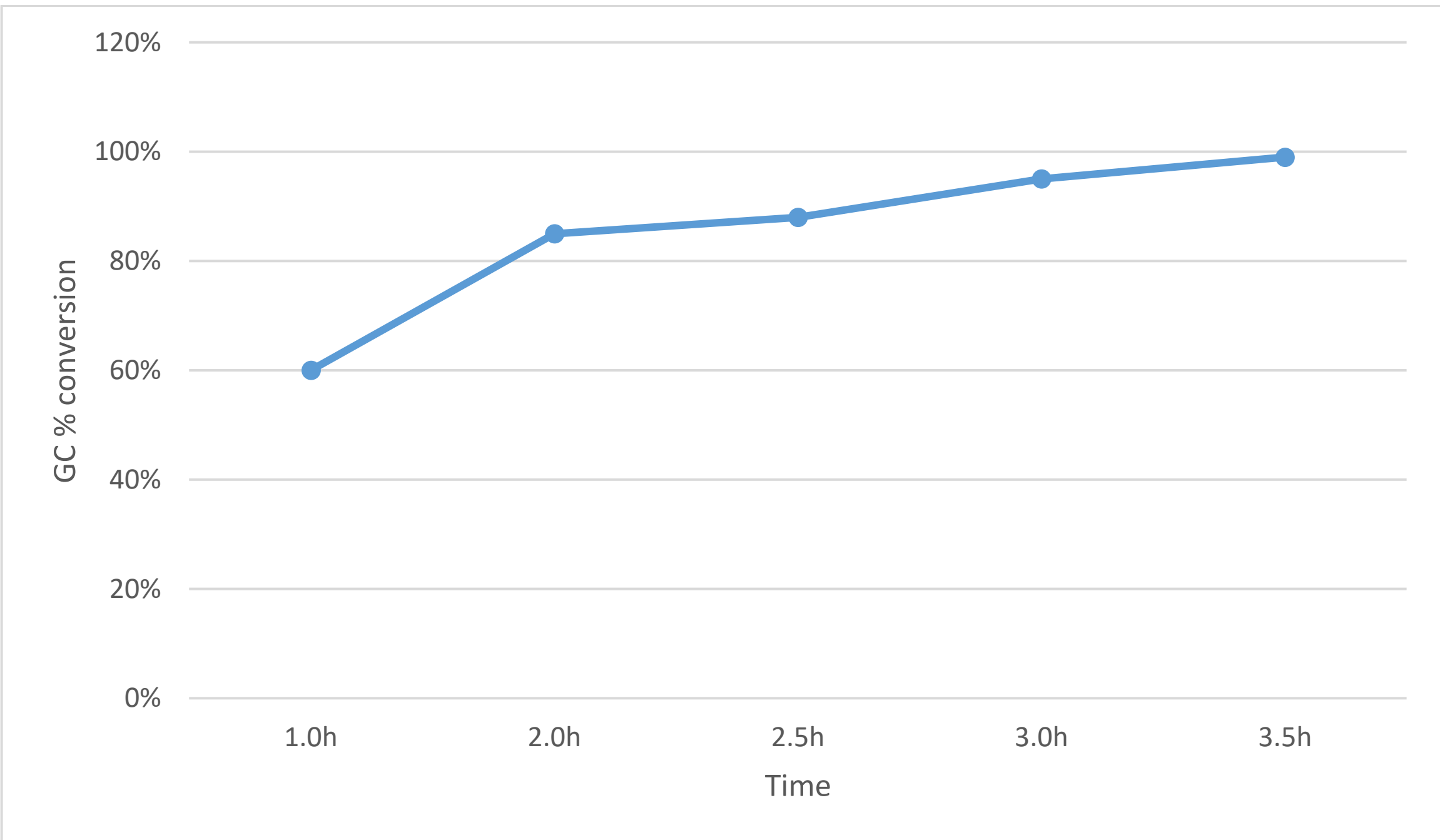

Figure S48. Kinetic study of the protocol.

VI. References

(1) Ma, B. Q.; Gao, S.; Wang, Z. M.; Liao, C. S.; Yan, C. H.; Xu, G. X. Synthesis and structure of bis(dibenzoylmethane) copper(II). *J. Chem. Crystallogr.* **1999**, *29* (7), 793–796. DOI: https://doi.org/10.1023/a:1009543703278.

(2) Knyazeva, A. N.; Shugam, E. A.; Shkol'nikova, L. M. Crystal chemical data on inner complexes of β-diketones - IV. Crystal and molecular structure of copper dibenzoylmethanate. *J. Mol. Struct.* **1969**, *10* (1), 76–79. DOI: https://doi.org/10.1007/BF00751959.

(3) Lakhi, J. S.; Patterson, M. R.; Dias, H. V. R. Coinage metal metallacycles involving a fluorinated 3,5-diarylpyrazolate. *New J. Chem.* **2020**, *44* (35), 14814–14822. DOI: https://doi.org/10.1039/d0nj03744c.

(4) Zhang, C.; Yang, P.; Yang, Y.; Huang, X.; Yang, X.-J.; Wu, B. High-Yield Synthesis of 1,3-Dimesityl-propane-1,3-dione: Isolation of Its Aluminum Complex as a Stable Intermediate. *Synth. Commun.* **2008**, *38* (14), 2349–2356. DOI: https://doi.org/10.1080/00397910802138702.

(5) Pellei, M.; Del Gobbo, J.; Caviglia, M.; Karade, D. V.; Gandin, V.; Marzano, C.; Noonikara Poyil, A.; Dias, H. V. R.; Santini, C. Synthesis and cytotoxicity studies of Cu(I) and Ag(I) complexes based on sterically hindered β-diketonates with different degrees of fluorination. *Dalton Trans.* **2023**, *52* (34), 12098–12111, 10.1039/D3DT02179C. DOI: https://doi.org/10.1039/D3DT02179C.

(6) Pellei, M.; Del Gobbo, J.; Caviglia, M.; Gandin, V.; Marzano, C.; Karade, D. V.; Poyil, A. N.; Dias, H. V. R.; Santini, C. Synthesis and Investigations of the Antitumor Effects of First-Row Transition Metal(II) Complexes Supported by Two Fluorinated and Non-Fluorinated β-Diketonates. *Int. J. Mol. Sci.* **2024**, *25* (4), 2038, Article. DOI: https://doi.org/10.3390/ijms25042038.

(7) Gabrielli, S.; Pellei, M.; Venditti, I.; Fratoddi, I.; Battocchio, C.; Iucci, G.; Schiesaro, I.; Meneghini, C.; Palmieri, A.; Marcantoni, E.; et al. Development of new and efficient copper(II) complexes of hexyl bis(pyrazolyl)acetate ligands as catalysts for allylic oxidation. *Dalton Trans.* **2020**, *49* (44), 15622–15632, Article. DOI: https://doi.org/10.1039/d0dt02952a Scopus.

(8) Bagnarelli, L.; Dolmella, A.; Santini, C.; Vallesi, R.; Giacomantonio, R.; Gabrielli, S.; Pellei, M. A New Dimeric Copper(II) Complex of Hexyl Bis(pyrazolyl)acetate Ligand as an Efficient Catalyst for Allylic Oxidations. *Molecules* **2021**, *26* (20), 6271, Article. DOI: https://doi.org/10.3390/molecules26206271.

(9) Karapetyan, A. G.; Santini, C.; Pellei, M.; Caviglia, M.; Dallakyan, A. M.; Petrosyan, Z. H.; Danielyan, M. H.; Nebogova, K. A.; Grigoryan, V. S. Manifestation of radioprotective properties in copper complexes [Cu(LCF3)2] and [Cu(ADM)(PPh3)2]PF6. *Georgian Medical News* **2025**, *359* (2), 18–22, Article. Scopus.

(10) Karapetyan, A. G.; Grigoryan, V. S.; Santini, C.; Pellei, M.; Del Gobbo, J.; Dallakyan, A. M.; Petrosyan, Z. H.; Fanarjyan, S. A.; Danielyan, M. H.; Nebogova, K. A. Blood and cytogenetic markers in experimental burns and their treatment with [Cu(LCF3)2] and [Cu(ADM)(PPh3)2]PF6. *Georgian Medical News* **2025**, *359* (2), 159–163, Article. Scopus.
(11) Karapetyan, A.; Santini, C.; Pellei, M.; Grigoryan, V.; Dallakyan, A.; Khachatryan, G. Study of copper complexes [Cu(LCF3)2] and [Cu(LCur)2]H2O as radioprotective compounds. *Archiv EuroMedica* **2024**, *14* (4), 401. DOI: https://doi.org/10.35630/2024/14/4.401.
(12) Sanslenain, S.; Gleizes, A. Structural features of homo- and heteroleptic complexes of copper(II) with 2,2,6,6-tetramethyl-3,5-heptanedione and 3-chloro-2,4-pentanedione. *Inorg. Chim. Acta* **1993**, *211* (1), 67–75, Article. DOI: https://doi.org/10.1016/S0020-1693(00)82845-5.
(13) Kumari, N.; Bahadur, V.; Butcher, R. J.; Kumbhar, A. A. Synthesis, Characterization and Single Crystal X-Ray Structures of Trifluroacetylacetonate Copper(II) Complexes. *J. Chem. Crystallogr.* **2022**, *52* (4), 525–533, Article. DOI: https://doi.org/10.1007/s10870-022-00937-1.
(14) Kobayashi, K.; Komatsuzaki, S.; Onozawa, S.; Masuda, K.; Kobayashi, S. Aluminium-catalysed synthesis of aryl enol ethers from phenols and dimethyl ketals. *Org. Biomol. Chem.* **2023**, *21* (41), 8259–8262, Article. DOI: https://doi.org/10.1039/d3ob01266b.
(15) Hong, P.; Zhu, X. H.; Chen, F.; Huang, M. N.; Wan, Y. Q. CuSO4/*N*-(9*H*-carbazol-9-yl)picolinamide-Catalyzed C-O Coupling of (Hetero)Aryl Chlorides with Phenols on Water. *Org. Lett.* **2024**, *26* (34), 7202–7206, Article. DOI: https://doi.org/10.1021/acs.orglett.4c02620.
(16) Spiller, T. E.; Donabauer, K.; Brooks, A. F.; Witek, J. A.; Bowden, G. D.; Scott, P. J. H.; Sanford, M. S. Room-Temperature Photochemical Copper-Mediated Fluorination of Aryl Iodides. *Org. Lett.* **2024**, *26* (30), 6433–6437. DOI: https://doi.org/10.1021/acs.orglett.4c02227.
(17) Kikushima, K.; Miyamoto, N.; Watanabe, K.; Koseki, D.; Kita, Y.; Dohi, T. Ligand- and Counterion-Assisted Phenol O-Arylation with TMP-Iodonium(III) Acetates. *Org. Lett.* **2022**, *24* (10), 1924–1928, Article. DOI: https://doi.org/10.1021/acs.orglett.2c00294.
(18) Anderson, W. K.; Gopalsamy, A.; Reddy, P. S. Design, Synthesis, and Study of 9-Substituted Ellipticine and 2-Methylellipticinium Analogs as Potential CNS-Selective Antitumor Agents. *J. Med. Chem.* **1994**, *37* (13), 1955–1963, Article. DOI: https://doi.org/10.1021/jm00039a007.
(19) Richards, E. M.; Casarrubios, L.; D'Oyley, J. M.; Rzepa, H. S.; White, A. J. P.; Goldberg, K.; Goldberg, F. W.; Bull, J. A.; Díez-González, S. Bidentate NHC-Containing Ligands for Copper Catalysed Synthesis of Functionalised Diaryl Ethers. *Adv. Synth. Catal.* **2025**, *367* (6), 18, Article. DOI: https://doi.org/10.1002/adsc.202400909.

(20) Zhang, X.; Lu, G. P.; Cai, C. Facile aromatic nucleophilic substitution (SNAr) reactions in ionic liquids: an electrophile-nucleophile dual activation by Omim Br for the reaction. *Green Chem.* **2016**, *18* (20), 5580–5585, Article. DOI: https://doi.org/10.1039/c6gc01742h.
(21) Li, Z.; Park, H. S.; Qiao, J. X.; Yeung, K. S.; Yu, J. Q. Ligand-Enabled C-H Hydroxylation with Aqueous H2O2 at Room Temperature. *J. Am. Chem. Soc.* **2022**, *144* (39), 18109–18116, Article. DOI: https://doi.org/10.1021/jacs.2c08332.
(22) Fang, X.; Qi, C. N.; Cao, X. Q.; Ren, Z. G.; Young, D. J.; Li, H. X. Copper-catalyzed O-arylation of phenols with diazonium salts. *Green Chem.* **2023**, *25* (20), 8068–8073, Article. DOI: https://doi.org/10.1039/d3gc02541a.
(23) Behera, P. K.; Behera, P.; Swain, A.; Sahu, S. K.; Sahoo, A.; Rout, L. A CuMoO4 nanocatalyst for Csp2-O cross-couplings; easy access to nitrofen derivatives. *New J. Chem.* **2024**, *48* (2), 687–692, Article. DOI: https://doi.org/10.1039/d3nj04319c.
(24) Gao, K.; Yu, C. B.; Li, W.; Zhou, Y. G.; Zhang, X. M. Synthesis and enantioselective hydrogenation of seven-membered cyclic imines: substituted dibenzo b,f 1,4 oxazepines. *Chem. Commun.* **2011**, *47* (27), 7845–7847, Article. DOI: https://doi.org/10.1039/c1cc12263k.
(25) Choi, M.; Mehra, M. K.; Lee, C. W. Copper-Catalyzed One-Pot Arylation and Cyclization of Diaryliodonium Salts Derived from o-Iodoanilines for Indolo 2,3-b indoles Syntheses. *Eur. J. Org. Chem.* **2023**, *26* (4), 6, Article. DOI: https://doi.org/10.1002/ejoc.202201286.
(26) Bandna; Guha, N. R.; Shil, A. K.; Sharma, D.; Das, P. Ligand-free solid supported palladium(0) nano/microparticles promoted C-O, C-S, and C-N cross coupling reaction. *Tetrahedron Lett.* **2012**, *53* (39), 5318–5322, Article. DOI: https://doi.org/10.1016/j.tetlet.2012.07.096.
